# The Human Cytomegalovirus DNA Polymerase Processivity Factor UL44 Is Modified by SUMO in a DNA-Dependent Manner

**DOI:** 10.1371/journal.pone.0049630

**Published:** 2012-11-15

**Authors:** Elisa Sinigalia, Gualtiero Alvisi, Chiara V. Segré, Beatrice Mercorelli, Giulia Muratore, Michael Winkler, He-Hsuan Hsiao, Henning Urlaub, Alessandro Ripalti, Susanna Chiocca, Giorgio Palù, Arianna Loregian

**Affiliations:** 1 Department of Molecular Medicine, University of Padua, Padua, Italy; 2 Department of Hematology and Medical Oncology Seragnoli, University of Bologna, Bologna, Italy; 3 Department of Infectious Diseases, Molecular Virology, University of Heidelberg, Heidelberg, Germany; 4 European Institute of Oncology, Milan, Italy; 5 Leibniz-Institut für Primatenforschung, Goettingen, Germany; 6 Bioanalytical Mass Spectrometry Group, Max Planck Institute for Biophysical Chemistry, Goettingen, Germany; 7 Bioanalytics, Department of Clinical Chemistry, University Medical Center, Goettingen, Germany; 8 Azienda Ospedaliero-Universitaria di Bologna, Policlinico S. Orsola-Malpighi, Bologna, Italy; Universität Erlangen-Nürnberg, Germany

## Abstract

During the replication of human cytomegalovirus (HCMV) genome, the viral DNA polymerase subunit UL44 plays a key role, as by binding both DNA and the polymerase catalytic subunit it confers processivity to the holoenzyme. However, several lines of evidence suggest that UL44 might have additional roles during virus life cycle. To shed light on this, we searched for cellular partners of UL44 by yeast two-hybrid screenings. Intriguingly, we discovered the interaction of UL44 with Ubc9, an enzyme involved in the covalent conjugation of SUMO (Small Ubiquitin-related MOdifier) to cellular and viral proteins. We found that UL44 can be extensively sumoylated not only in a cell-free system and in transfected cells, but also in HCMV-infected cells, in which about 50% of the protein resulted to be modified at late times post-infection, when viral genome replication is accomplished. Mass spectrometry studies revealed that UL44 possesses multiple SUMO target sites, located throughout the protein. Remarkably, we observed that binding of UL44 to DNA greatly stimulates its sumoylation both *in vitro* and *in vivo*. In addition, we showed that overexpression of SUMO alters the intranuclear distribution of UL44 in HCMV-infected cells, and enhances both virus production and DNA replication, arguing for an important role for sumoylation in HCMV life cycle and UL44 function(s). These data report for the first time the sumoylation of a viral processivity factor and show that there is a functional interplay between the HCMV UL44 protein and the cellular sumoylation system.

## Introduction

Most replicative DNA polymerases include a catalytic subunit, responsible for DNA polymerization, and a processivity factor that holds the catalytic subunit on DNA to allow continuous DNA synthesis. One of the best-studied processivity factors is proliferating cell nuclear antigen (PCNA) of eukaryotic DNA polymerases δ and ε [1]. PCNA, which belongs to the family of so-called “sliding clamps”, has no inherent DNA-binding capacity, but with the aid of clamp loader proteins is assembled onto DNA as a toroidal homotrimer [2]. In addition to DNA replication, PCNA has been implicated in DNA recombination and repair, as well as in DNA methylation, chromatin remodeling, and cell cycle regulation [1]. Consistent with its pleiotropic functions, it interacts with a plethora of proteins [3] and undergoes a number of post-translational modifications, including phosphorylation, acetylation, ubiquitination and sumoylation, which are believed to regulate its subcellular localization, stability and protein binding specificity [4], [5], [6].

The human cytomegalovirus (HCMV) DNA polymerase includes a catalytic subunit, UL54 (the *UL54* gene product), and an accessory, homodimeric subunit, UL44 (the *UL44* gene product), that binds DNA without the aid of clamp loaders [7] yet wraps around DNA akin to PCNA [8]. While UL44 shows no apparent sequence homology with PCNA, there is striking structural similarity between UL44 and PCNA monomers [2],[9]. Similarly to PCNA, UL44 is a phosphoprotein [10]. Intriguingly, the phosphorylation state of UL44 has been shown to regulate its nuclear import rate by controlling its interaction with host cell factors [11], [12], [13]. The best-characterized function of UL44 during HCMV infection is that of binding to UL54 through a region named connector loop [14],[15],[16], stimulating its activity and conferring processivity to the holoenzyme [17], [18]. However, UL44 continues to accumulate to strikingly high levels at late times after infection, when DNA replication is accomplished [19], [20]. Its early-late kinetics of transcription and the high level of expression suggest that UL44 might play additional roles during the viral life cycle.

To investigate this possibility, we conducted yeast two-hybrid (Y2H) screenings to search for cellular partners of UL44. To our surprise, Ubc9, an enzyme involved in the sumoylation process, was identified as a UL44 protein interaction partner. Sumoylation is a post-translational protein modification analogous to ubiquitination. It consists of reversible and covalent conjugation of SUMO (Small Ubiquitin-related MOdifier) to a protein target [21], [22]. In the sumoylation cascade, the C-terminus of SUMO is activated by an activating enzyme (E1), transferred to a conjugating enzyme (E2, that is Ubc9), and linked to a lysine residue of the substrate protein with the aid of a ligase (E3). Mainly, three SUMO paralogs (SUMO-1, -2, -3) have been identified so far [23], [24]. SUMO-2 and SUMO-3 are highly homologous to one another (95% identity) while they differ from SUMO-1 by 50%. Conjugation of SUMO-1 has been shown to play a functional role in a number of biological processes, ranging from nucleocytoplasmic transport to transcription, the maintenance of genome stability, nucleic acid DNA metabolism, cell signaling, and many others [21], whereas the role of SUMO-2/−3 modification is less clear.

Here we report that the association of Ubc9 and UL44 leads to conjugation of SUMO molecules on multiple lysine residues. Both SUMO-1 and SUMO-2/3 were found to be conjugated to UL44. Sumoylation of UL44 was detected not only *in vitro* and in transiently transfected cells but, more importantly, also in HCMV-infected human cells during virus replication. Interestingly, we observed that binding of UL44 to DNA greatly stimulates SUMO conjugation to the protein both *in vitro* and in cells. In addition, we show that overexpression of SUMO-1 alters the intranuclear distribution of UL44 in HCMV-infected cells, and enhances both viral DNA replication and virus production in an Ubc9-dependent manner. These data represent the first report of sumoylation of a viral processivity factor and show that there is a complex interplay between the HCMV UL44 protein and the cellular sumoylation system.

## Materials and Methods

### Plasmids

The Y2H plasmids expressing LexA-UL44 and LexA-Ubc9 were generated by cloning the *UL44* and *Ubc9* coding sequences from pRSET44 (a gift of P. F. Ertl, GlaxoSmithKline, UK) and pACT2-Ubc9 (from G. Gao, Chinese Academy of Sciences, Beijing, China) respectively, in pBTMK, derived from pBTM116 [25]. The pACT-UL44 and pACT2-Ubc9 plasmids, encoding GAD-UL44 and GAD-Ubc9 fusions, respectively, have been described in [26], [27]. The plasmid expressing GAD-UL54 was created by cloning the *UL54* coding sequence from pRSET-Pol (a gift of P. F. Ertl) in pACT2 (Clontech). Plasmid pRSET44 was used to express 6His-UL44 in *Escherichia coli*. Plasmid pRSET-Ubc9 was constructed by cloning the *Ubc9* coding sequence from pACT2-Ubc9 in pRSET (Invitrogen). Plasmid pCDNA3-PB1, used for *in vitro* transcription of the PB1 subunit of influenza A virus RNA polymerase, was described previously [28]. Plasmids pD15-GST and pD15-UL44, which express GST and GST-UL44, respectively, have been described in [29]. Plasmid pTE1E2S1 [30] was provided by H. Saitoh (Kusamoto University, Japan). Plasmids GFP-UL44, pDESTnV5-UL44, and pDESTnV5-UL44ΔNLS have been described previously [11], [31]. Plasmids pDsRed2-Ubc9 [27] and pDsRed2-UL53 [32] were kindly provided by G. Gao (Chinese Academy of Sciences, Beijing, China) and D. Camozzi (University of Bologna, Italy), respectively. Plasmid pCDNA3.1-UL44-FLAG (from M. Marschall, Universitat Erlangen-Numberg, Germany) was used to express C-terminally FLAG-tagged UL44 [33], while plasmid pDEST-nFLAG [34] was used to express N-terminally FLAG-tagged UL44 [35], UL44Δloop [36] and UL44L86A/L87A in mammalian cells. Plasmids pCDNA3-Ubc9, pCDNA3-Ubc9C93S, pCDNA3-HA-SUMO-1, pCDNA3-HA-SUMO-2, and pCDNA3-HA-SUMO-3 used in overexpression experiments in mammalian cells were a gift of R. T. Hay (University of Dundee, UK). The deletion mutants LexA-UL44_1–100_, LexA-UL44_1–200_, LexA-UL44_1–300_, LexA-UL44_1–350_, LexA-UL44_1–390_, and LexA-UL44_1–420_ were generated by PCR amplification of plasmid pBTMK-UL44 with appropriate primers (Table S1). The LexA-UL44_114–433_, LexA-UL44_201–433_, and LexA-UL44_313–433_ constructs were generated by deleting part of the *UL44* coding sequence from pBTMK-UL44 with restriction enzymes. Plasmids pDESTnFLAG-UL44(1–300) and pDESTnFLAG-UL44(313–433) were generated using the Gateway Technology (see Supplementary Material and Methods in Text S1). All other UL44 mutants and the Ubc9C93S mutant were obtained by using the QuikChange mutagenesis kit (Stratagene) with primers containing appropriate nucleotide change(s). More details on plasmid construction and mutagenesis are given in Supplementary Material and Methods in Text S1. The sequences of all primers used in this work are reported in Table S1. All DNA sequences were confirmed by sequencing.

#### Y2H screenings and interaction assays

Growth media and standard methods for manipulating yeast cells were as described [37]. *Saccharomyces cerevisiae* strain L40 was transformed [38] with the bait plasmid pBTMK-UL44 and subsequently with either of two cDNA libraries fused to GAD (see Supplementary Material and Methods in Text S1). Primary transformants were selected for growth on -His-Leu-Trp dropout plates. His^+^ colonies were thereafter analyzed for β-galactosidase activity by filter lift experiments [39]. Double positive clones were subjected to another cycle of screening (for further details see Supplementary Material and Methods in Text S1). cDNA inserts of interactor plasmids were sequenced and analyzed with BLAST (www.ncbi/blastn). To quantify β-gal expression, the method of Breeden and Nasmyth [40] was used.

#### Proteins

*E. coli*-expressed, purified GST and GST- or 6His-tagged UL44 proteins were obtained as previously described [29], with modifications (Supplementary Material and Methods in Text S1). In some preparations, samples were treated with polymin P as described [41] to eliminate residual bacterial nucleic acids. Preparation of UL44 SUMO-modified in *E. coli* was accomplished as described in Supplementary Material and Methods in Text S1.

#### GST-pulldown assays

Assays were performed using GST and GST-UL44 and *in vitro*-translated UL54, PB1, or Ubc9 as previously described [29], with modifications (Supplementary Material and Methods in Text S1). *In vitro* transcription-translation of proteins was performed from the appropriate plasmid by using the TNT T7 coupled transcription-translation system (Promega). The translation products were labeled with [^35^S]methionine (Amersham Pharmacia Biotech).

#### *In vitro* sumoylation assays

The assays to test *in vitro* sumoylation of UL44 by SUMO-1, -2, and -3 were performed using purified wild-type and mutant 6His-UL44 or GST-UL44 fusion proteins and the SUMOlink SUMO-1 kit from Active Motif or the SUMOylation Kit from Enzo Life Science according to the manufacturer’s suggestions. In some experiments, double stranded (ds) DNA (e.g., activated calf thymus DNA from Amersham Pharmacia Biotec or salmon testes DNA from Sigma) or single-stranded (ss) DNA (e.g., single-stranded calf lung DNA, from Crinos, Como, Italy) was added to the reaction mixture at a final concentration of 500 nM.

### Cells and Virus

Human foreskin fibroblasts (HFF; from the American Type Culture Collection [ATCC]), HeLa (from ATCC), eco Phoenix (a generous gift from G. P. Nolan, Stanford, USA; [42]), and Human Embryonic Kidney 293T (HEK 293T; from ATCC) cells were maintained in Dulbecco’s modified Eagle’s medium (DMEM, Life Biotechnologies) supplemented with 10% fetal calf serum (FCS), 100 U/ml penicillin and 100 µg/ml streptomycin (P/S). COS-1 cells (from ATCC) were maintained in DMEM with 5% FCS and P/S. The U373-SUMO-1 cell line, which constitutively expresses FLAG-tagged SUMO-1 [43], and the control U373-Neo cell line, stably transfected with an empty vector carrying a Neomycin resistance marker [44], were kindly provided by G. S. Hayward (Johns Hopkins University School of Medicine, Baltimore, USA) and were maintained in medium containing 0.5 mg/ml Neomycin (G418, Gibco-BRL). HCMV strain AD169 was purchased from ATCC.

### *In vivo* Sumoylation Assays

To analyze UL44 sumoylation in transient expression assays, HeLa or Phoenix cells were transfected for 48 h with appropriate plasmids using the calcium phosphate precipitation method. For analysis of UL44 sumoylation during HCMV replication, HFF, U373-Neo, and U373-SUMO-1 cells were mock-infected or infected with HCMV at a multiplicity of infection (MOI) of 5 PFU/cell. Cells were harvested at different time points after infection and analyzed by western blotting as described below.

### Western Blot and Immunoprecipitation Analysis

Transfected or infected cells were lysed in an appropriate volume of buffer I (5% SDS, 0.15 M Tris-HCl pH 6.8, 30% glycerol) diluted 1∶3 in buffer II (25 mM Tris-HCl pH 8, 50 mM NaCl, 0.5% NP-40, 0.5% sodium deoxycholate, 0.1% SDS) supplemented with Complete protease inhibitors (Roche Molecular Biochemicals) and 5 mM N-ethylmaleimide (NEM). Lysates were then incubated on ice for 20 minutes and boiled at 95°C for 10 min. Proteins were separated by SDS-PAGE, electroblotted onto a polyvinylidene fluoride membrane (Bio-Rad), and analyzed by western blotting with indicated antibodies (details are given in Supplementary Material and Methods in Text S1).

For immunoprecipitation analysis, lysates were diluted 1∶5 in E1A buffer (50 mM Hepes pH 7.5, 250 mM NaCl, 0.1% NP-40) supplemented with Complete protease inhibitors and 5 mM NEM. Immunoprecipitation was performed with 2–3 mg of total lysate using a ratio of 3–7 µg antibody/mg of total proteins (see Supplementary Material and Methods in Text S1 for details on antibodies) and protein A-Sepharose beads. For co-immunoprecipitation analysis, cells were lysed in E1A buffer supplemented with Complete protease inhibitors and 5 mM NEM, and successively co-immunoprecipitations were performed with 1.5–5 mg of total lysate and 50 µl of 50% slurry of anti-FLAG-M2-Agarose beads (Sigma).

### Mass Spectrometric (MS) Determination of Sumoylation Sites of UL44

Mass spectrometric identification of sumoylated lysine residues within UL44 was performed after in-gel-digestion of *E. coli-*expressed and SUMO-modified UL44 with endoproteinase Trypsin. Extracted peptides were analyzed by LC-MSMS on an Orbitrap Velos (ThermoFisherScientific) exactly under the conditions described in Hsiao et al. [45]. Data analysis was performed by the use of software “ChopNSpice” [45] in combination with MASCOT as search engine. See Supplementary Material and Methods in Text S1 for details.

### Immunofluorescence and Confocal Microscopy Analysis

For confocal laser-scanning microscopy (CLSM) analysis, COS-1 were transfected using the Arrest-IN™ (Biosystems) reagent, according to the manufacturer’s recommendations. At 24 h post-transfection, cells were fixed with 4% paraformaldehyde. Cells were imaged using a Leica TCS-SP2 confocal microscope equipped with a 63× oil immersion objective.

For analysis of UL44 intranuclear localization in HCMV-infected U373-SUMO-1 and U373-Neo cell lines, cells were seeded at 2.5 × 10^5^/well on glass coverslips in 6-well plates and allowed to attach. The next day, cells were infected with HCMV AD169 at an MOI of 1 or of 5 PFU/cell. Cells were fixed in 4% paraformaldehyde in PBS for 15 min at room temperature, and then permeabilized with acetone for 2 min at –20°C. After washing extensively with PBS, cells were incubated first with 4% FBS in PBS for 1 h at room temperature and then with a primary mouse monoclonal antibody against UL44 (10-C50, Fitzgerald Industries International) at a dilution of 1∶100 in FBS 4% in PBS for 1 h at 37°C. Cells were then washed extensively with 4% FBS in PBS and incubated with a secondary goat anti-mouse fluorescein-conjugated antibody (Ig-FITC, Chemicon International) at a dilution of 1∶1000 for 1 h at 37°C. Cells were successively washed with PBS and mounted in 70% glycerol in PBS. For better visualization, cells were counterstained with Evans Blue and analyzed also for red fluorescence. CLMS analysis was then performed as described above.

### Quantitative Real-time PCR (qPCR)

To analyze the effects of SUMO-1 overexpression on viral DNA synthesis, U373-Neo and U373-SUMO-1 cells transduced with either shUbc9 or non-silencing lentiviral particles (see below) or non-transduced, were seeded at a density of 5×10^4^ per well in 24-well plates. The next day, cells were infected with HCMV AD169 at an MOI of 1 PFU/cell. At 72 h post-infection (p.i.), cells were collected and total DNA was extracted using the QiAmp DNA Extraction Kit (Qiagen). The levels of viral DNA were then determined by qPCR and normalized to the cellular β-globin gene copies as described [46].

### Virus Yield Assays

To analyze the effects of SUMO-1 overexpression on virus production, virus yield assays were performed as described previously [47], with some modifications. Briefly, U373-Neo and U373-SUMO-1 cells transduced with either shUbc9 or non-silencing lentiviral particles (see below) or non-transduced, 5 × 10^4^ cells per well were seeded in 24-well plates, incubated overnight, and infected with HCMV AD169 at an MOI of 1. At 120 h p.i., cells were subjected to one cycle of freezing and thawing, and titers were determined by transferring 100-µl aliquots from each of the wells to a fresh 96-well monolayer culture of HFF cells followed by 1∶5 serial dilution across the plate. After incubation at 37°C for 7 days, cell monolayers were stained with crystal violet and plaques were counted.

### Lentivirus Production and Ubc9 Knock-down

Lentiviral particles were produced by transient transfection of HEK 293T cells with packaging plasmids helper Δ8.9 (Addgene) and helper Ampho (kindly provided by the Tissue Culture Facility at the IEO, Milan, Italy) and either pTRIPZ-shUBC9 vector (Open Biosystems) or non-silencing pTRIPZ control vector (Open Biosystems) by the calcium phosphate precipitation method. Supernatants were collected at 48 h post-transfection and used for the transduction of U373-SUMO-1 and U373-Neo target cells in the presence of polybrene (8 µg/ml, Sigma). Selection was done in puromycin (0.5 µg/ml, Invitrogen) for two weeks prior to 4-day doxycycline (1 µg/ml, Sigma) induction to obtain *Ubc9* silencing. Ubc9-knocked-down cells were screened for Red Fluorescent Protein expression and used for further experiments.

## Results

### Identification of Human Ubc9 as a Cellular Interaction Partner of UL44

To identify cellular proteins that interact with UL44, Y2H screens were carried out with a bait consisting of full-length UL44 protein (amino acids 1–433) fused to the *E. coli* LexA protein. Control experiments demonstrated that the LexA-UL44 protein did not activate expression of either *HIS3* or *lacZ* reporter gene by itself and, as expected [9], [14], could both interact with UL54 and dimerize (Table 1 and data not shown). Thus, this bait was used to screen two different cellular cDNA libraries fused to *S. cerevisiae* GAL4 activation domain (GAD), one derived from human B lymphocytes [48] and the other from promyelocytic HL-60 cells [49]. A total of 167 or 85 colonies, respectively, were positive for both *HIS3* and *lacZ* reporter genes. Plasmids encoding putative interactors of UL44 were isolated from double-positive clones and retransformed into yeasts expressing LexA-UL44 in order to confirm the interaction. The 28 and 13 positive clones after this retransformation, respectively, were sequenced.

**Table 1:** UL44 interacts with human Ubc9 in yeast two-hybrid assays.

Hybrid^1^		
DNA-binding domain fusion	Activation domain fusion	β-gal expression (Units ± S.D.)^2^
LexA-UL44	/	−(<1)
LexA-UL44	GAD	−(<1)
/	GAD-UL54	−(<1)
LexA	GAD-UL54	±(1043±213)
LexA-UL44	GAD-UL54	+(8244±245)
/	GAD-UL44	−(<1)
LexA	GAD-UL44	−(<1)
LexA-UL44	GAD-UL44	+(7643±397)
LexA-Ubc9	/	−(<1)
LexA-Ubc9	GAD	−(<1)
LexA-Ubc9	GAD-UL44	+(6915±343)
/	GAD-Ubc9	−(<1)
LexA	GAD-Ubc9	−(<1)
LexA-UL44	GAD-Ubc9	+(7012±214)

1 UL44, UL54, and Ubc9 proteins were fused to the C-terminus of LexA protein and/or of GAL4 activation domain (GAD). Fusion proteins were then assayed for interaction by qualitative β-galactosidase (β-gal) filter assays and by quantitative β-gal liquid assays.

2 β-gal expression was scored as follows +, strong blue color detected within 2 h of incubation; ±, blue color detected after more than 2 h of incubation; −, no signal detected after 16–24 h of incubation. Values within parentheses represent β-gal units ± standard deviation (SD) of 3–4 yeast colonies from at least three independent transformations.

In total, from the two screenings we identified 7 cellular UL44-binding proteins; here we report the identification of human Ubc9 as a specific interaction partner of UL44. In particular, 15 out of 28 clones of B lymphocytes library and 6 out of 13 clones of the HL-60 cells library contained the whole *Ubc9* coding sequence, plus 5′ and 3′ untranslated regions which varied in length in different clones. By co-transformation experiments with each individual interactor clone expressing Ubc9 and the LexA vector, it was excluded that Ubc9 could activate the reporter genes in the absence of the bait protein (Table 1). Ubc9 specifically interacted with UL44 in Y2H assays also in the reverse combination, i.e., with Ubc9 fused to LexA and UL44 fused to GAD (Table 1). In addition, in quantitative assays the β-galactosidase activity of yeasts expressing both UL44 and Ubc9 turned out to be comparable to that of yeasts expressing UL44 and UL54 (Table 1), which served as a positive control.

### UL44 Physically Interacts with Ubc9 through N-terminal and C-terminal Regions

**Figure 1:**
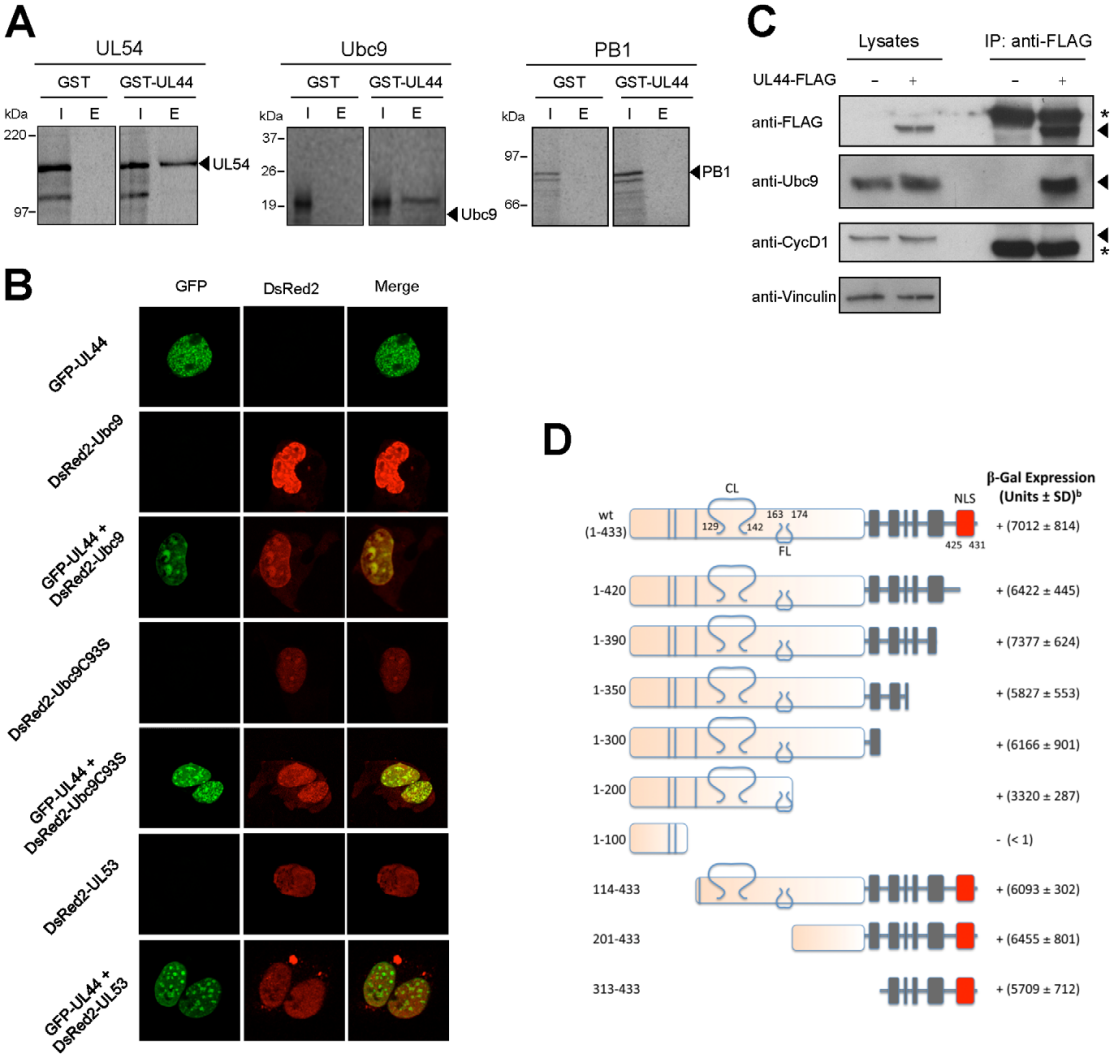
HCMV UL44 interacts with human Ubc9. (**A**) GST-pulldown assays showing interaction of purified GST or GST-UL44 with *in vitro*-expressed, radiolabeled Ubc9 (right panels), HCMV UL54 (central panels, positive control), and influenza A virus PB1 (left panels, negative control). I, input; E, eluted by glutathione. Arrowheads indicate the proteins of interest. (**B**) COS-1 cells were transfected to express the indicated GFP and DsRed2 fusion proteins and imaged by CLSM. Merged images of the green (GFP) and red (DsRed2) channels are shown on the right, with yellow coloration indicative of co-localization. (**C**) Phoenix cells were transfected to express a UL44-FLAG fusion. At 48 h post-transfection, cell lysates were analyzed by western blotting with anti-FLAG, anti-Ubc9, anti-CycD1, and anti-vinculin antibodies. Cell lysates were also incubated with anti-FLAG-M2-Agarose beads and the immunoprecipitated samples were analyzed by western blotting with anti-FLAG, anti-Ubc9, and anti-CycD1 antibodies. Asterisks indicate the IgG heavy chains, while arrowheads indicate the proteins of interest. (**D**) Several UL44 mutants were created by deleting different portions of *UL44* coding sequence and expressed as fusions with LexA. A diagram of the full-length and truncated UL44 proteins, with functional domains indicated, is reported. Numbers refer to remaining amino acid residues of UL44. Blue bars, residues important for dimerization; CL, connector loop; FL, flexible loop; grey boxes, glycine strings; red box, Nuclear Localization Signal. The ability of the mutants to physically interact with Ubc9 was determined by β-gal filter assays and scored as follows: +, strong blue color detected within 2 h of incubation; ±, blue color detected after more than 2 h of incubation; −, no signal detected after 16–24 h of incubation. Values within parentheses represent β-gal units ± standard deviation (SD) of 3–4 yeast colonies from at least three independent transformations, determined by quantitative β-gal assays.

Having identified Ubc9 as a potential interaction partner of UL44 in Y2H screens, we wished to confirm their physical interaction by an independent experimental approach. For this purpose, pulldown assays with a purified GST-UL44 protein and *in vitro*-translated, ^35^S-labeled Ubc9 were performed. As positive and negative controls, we also assayed the interaction between UL44 and *in vitro*-translated UL54 or the PB1 subunit of influenza A virus RNA polymerase. As expected, we could detect the interaction of UL54 [14], [29], but not of PB1, with GST-UL44 (Fig. 1A). Consistent with the Y2H results, Ubc9 specifically associated with GST-UL44, while no interaction with GST was observed (Fig. 1A).

Since our data suggested that UL44 can interact with Ubc9 both *in vitro* and in yeast cells, we sought to examine whether UL44 and Ubc9 could also co-localize in mammalian cells. To this end, aggregates distributed on a diffuse background fluorescence throughout the nucleus (Fig. 1B). As previously reported [50], when expressed alone Ubc9 exhibited a nuclear punctate pattern (Fig. 1B). Upon co-expression of GFP-UL44 and DsRed2-Ubc9, co-localization of UL44 we analyzed the subcellular localization of GFP-UL44 when transiently expressed either alone or in the presence of DsRed2-Ubc9. As a negative control, we also expressed the DsRed2-UL53 fusion protein, which localizes to the cell nucleus but does not interact with UL44 [32]. In GFP-UL44-transfected cells, UL44 localized in a large number of discrete nuclear with Ubc9 was observed (Fig. 1B). UL44 also co-localized with a catalytically impaired Ubc9 mutant (Ubc9C93S) [51], but not with UL53 [32]. In addition, co-immunoprecipitation experiments confirmed that UL44 could physically interact with endogenous Ubc9 in mammalian cells. The specificity of the interaction was confirmed by the inability of UL44 to co-immunoprecipitate with Cyclin D1 (CycD1; Fig. 1C).

To further explore the interaction between UL44 and Ubc9, we sought to map the domain of UL44 that interacts with Ubc9. To this aim, several N- and C-terminal deletion mutants of UL44 fused to LexA were generated and tested for the ability to interact with GAD-Ubc9 by Y2H assays. Control western blot experiments with an anti-LexA antibody evidenced protein bands of the expected molecular mass for all mutants (data not shown). As shown in Fig. 1D, the truncated protein UL44_1–300_, lacking most of the C-terminal disordered region of UL44, exhibited interaction with Ubc9. Further C-terminal truncation of UL44 revealed that a protein fragment corresponding to the first 200 amino acids of UL44 (UL44_1–200_) was still capable of interacting with Ubc9 (Fig. 1D). In contrast, the N-terminal 100 residues of UL44 (UL44_1–100_) exhibited no interaction with Ubc9 (Fig. 1D). Although unfolding of the UL44_1–100_ mutant protein cannot be excluded, these results suggested that this region of UL44 may not contain sequences important and/or sufficient for Ubc9 binding. Therefore, we analyzed the effects of N-terminal truncations. Deletion of the N-terminal 113 residues of UL44 (UL44_114–433_) did not impair the ability of UL44 to bind Ubc9. Similarly, the UL44_201–433_ mutant, lacking the N-terminal 200 amino acids, interacted with Ubc9. Interestingly, a mutant that only expresses the C-terminal 121 residues of UL44 (UL44_313–433_) still retained the ability to bind Ubc9 (Fig. 1D). Control Y2H experiments showed that none of the truncated UL44 proteins was able to activate transcription by itself (data not shown). Mapping studies in mammalian cells expressing UL44 deletion mutants showed that both the UL44_1–300_ and UL44_313–433_ mutants could immunoprecipitate endogenous Ubc9, similarly to wild-type UL44 (Fig. S1). Thus, our results suggest that UL44 contains two domains capable of independently binding to Ubc9, located at the N- and C-terminus of the protein (likely within residues 1–200 and 313–433, respectively).

### Ubc9 Mediates Extensive Sumoylation of UL44

**Figure 2:**
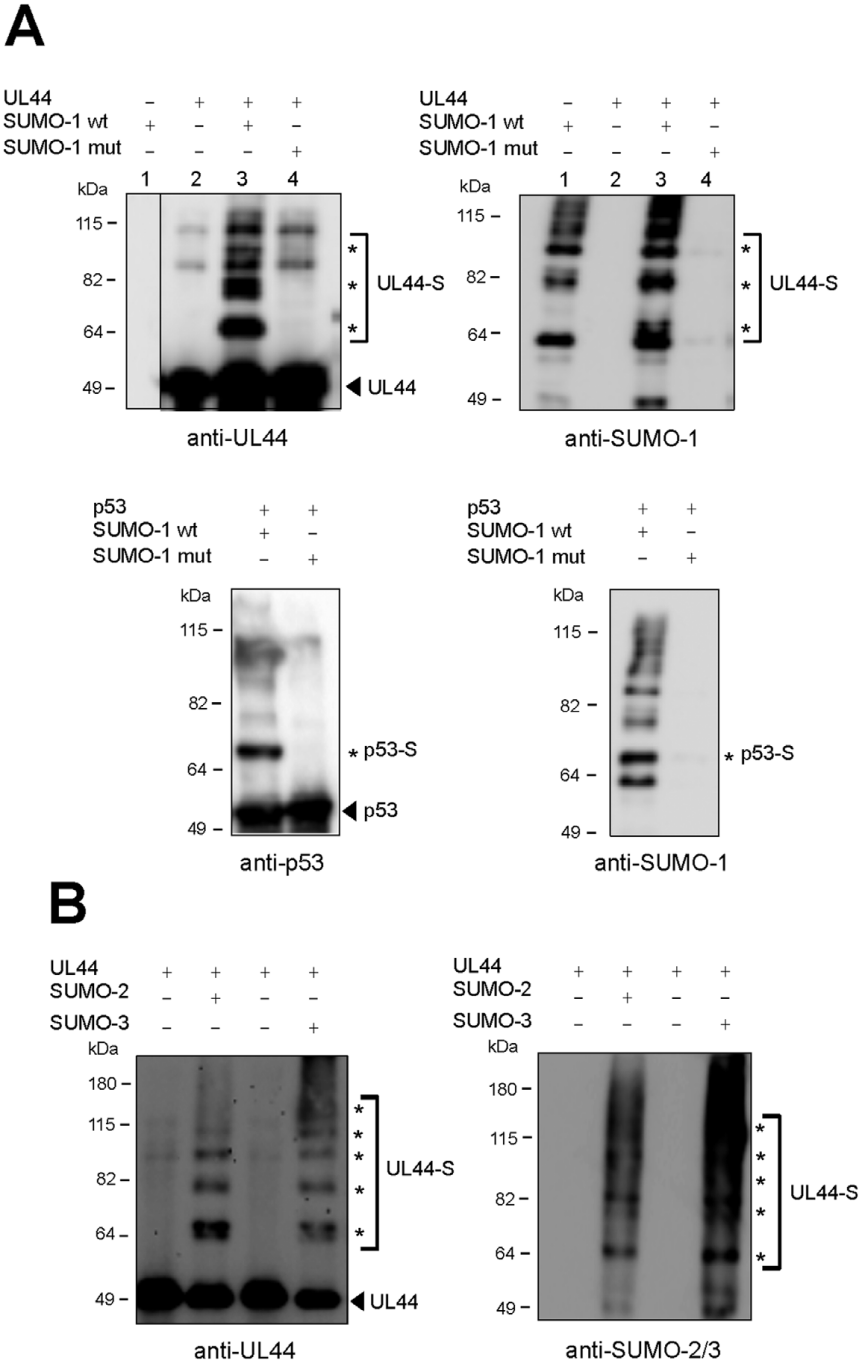
Sumoylation of UL44 *in vitro*. (**A**) To analyze UL44 sumoylation *in vitro*, purified 6His-UL44 was incubated in the absence or the presence of sumoylation enzymes and either wild-type SUMO-1 (SUMO-1 wt) or a mutant form of SUMO-1 (SUMO-1 mut) which cannot be covalently linked to substrates. The reaction products were analyzed by western blotting with anti-UL44 and anti-SUMO-1 antibodies. As a positive control, *in vitro* sumoylation of p53 was also analyzed. (**B**) Purified 6His-UL44 was incubated in the absence or the presence of sumoylation enzymes and either SUMO-2 or SUMO-3 and analyzed by western blotting with anti-UL44 and anti-SUMO-2/−3 antibodies. For all panels, the arrowhead indicates the unmodified form of UL44 or p53 and the asterisks indicate the respective sumoylated forms.

The observation that UL44 interacts with the SUMO-conjugating enzyme Ubc9 prompted us to investigate the possibility that UL44 may be sumoylated. This hypothesis was first tested in a cell-free system by incubating a purified 6His-UL44 fusion protein with purified Aos1/Uba2 (E1), Ubc9 (E2), and SUMO-1. As a control, sumoylation of human p53 was also examined by the same assay. As expected [52], [53], p53 was readily modified to give mainly a single mono-sumoylated product that reacted with both anti-p53 (Fig. 2A, left bottom panel) and anti-SUMO-1 (Fig. 2A, right bottom panel) antibodies (the bands appearing at high molecular weight, which are particularly visible in the anti-SUMO-1 panel, correspond to SUMO-E1 and -E2 enzymes conjugates). In a western blot analysis with an anti-UL44 antibody (Fig. 2A, left top panel), three main slower migrating forms of UL44 were observed (lane 3). The appearance of the ∼65, 80, and 95-kDa forms of UL44 was strictly dependent on the presence of SUMO-1, as the substitution of wild-type SUMO-1 with a mutant form which bears the Gly97-to-Ala change (SUMO-1 mut) and hence cannot be attached to target proteins, eliminated formation of these products (Fig. 2A, left top panel, lane 4). In addition, SUMO-1 modification was abolished if either SUMO-1, Aos1/Uba2, or Ubc9 was omitted from the reaction (not shown). A western blot analysis with an anti-SUMO-1 antibody confirmed that the slower migrating UL44 bands contained SUMO-1 (Fig. 2A, right top panel). Taken together, the above results established that UL44 is a substrate for *in vitro* SUMO-1 conjugation.

Having shown that UL44 can be modified by SUMO-1, we wondered whether it could be conjugated also to SUMO-2 and SUMO-3. Thus, the *in vitro* sumoylation system was applied to purified 6His-UL44 in the presence of sumoylation enzyme components and activated forms of SUMO-2 or SUMO-3. In these assays, the RanGTPase-activating protein RanGAP1 [54] was used as a positive control and as previously observed [55], was modified in the presence of SUMO-2 and SUMO-3 (data not shown). As shown in Fig. 2B, UL44 was also modified by either of the two SUMO peptides.

### UL44 is Sumoylated in the Mammalian Cell Nucleus

**Figure 3:**
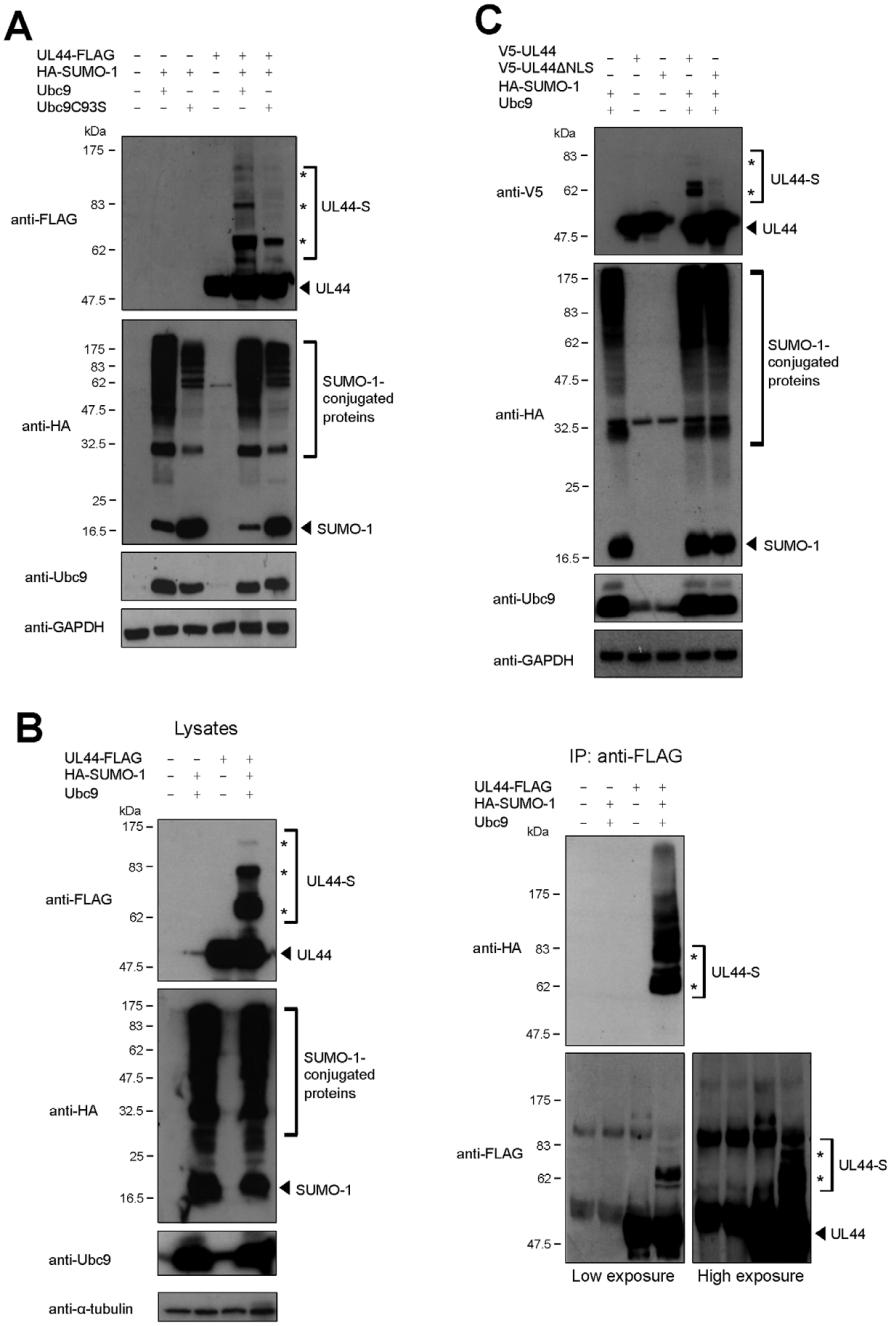
Sumoylation of UL44 in mammalian cells. (**A**) Phoenix cells were transfected to express the indicated proteins and analyzed by western blotting with anti-FLAG, anti-HA, anti-Ubc9, and anti-GAPDH antibodies. (**B**) Cell lysates were incubated with the anti-FLAG antibody and the input proteins (left panels) or the immunoprecipitated samples (right panels) were analyzed by western blot with the indicated antibodies. (**C**) Sumoylation of a UL44 mutant lacking the NLS was analyzed as in (A). For all panels, the arrowhead indicates the unmodified form of UL44 or free SUMO-1 and the asterisks indicate the sumoylated forms.

To verify whether UL44 could also be sumoylated in mammalian cells, we expressed UL44-FLAG and HA-SUMO-1 fusion proteins, in the presence of either wild-type Ubc9 or the catalytically impaired mutant Ubc9C93S in Phoenix cells and analyzed cell lysates by western blotting with anti-FLAG, anti-HA, and anti-Ubc9 antibodies. As expected, several bands corresponding to SUMO-1-conjugated proteins that reacted with an anti-HA antibody were detected upon co-expression of wild-type Ubc9, while less SUMO-1 conjugation was observed in the presence of the Ubc9C93S mutant (Fig. 3A). This was not due to differences in expression of the two Ubc9 variants, as evidenced by western blot analysis with the anti-Ubc9 antibody (Fig. 3A). When UL44-FLAG was expressed in the absence of HA-SUMO-1 and Ubc9, a single band with an apparent molecular mass of ∼50 kDa was detected using the anti-FLAG antibody. In contrast, slower migrating bands similar to those observed in *in vitro* sumoylated products (Fig. 2A) were observed upon co-expression of UL44-FLAG with HA-SUMO-1 and wild-type Ubc9 (Fig. 3A). These bands were significantly reduced in the presence of the Ubc9C93S mutant (Fig. 3A), demonstrating that they were dependent on the catalytic activity of Ubc9, with the residual slower migrating bands due to the activity of wild-type endogenous Ubc9.

To confirm that the observed products were indeed sumoylated forms of UL44, we expressed UL44-FLAG either in the absence or in the presence of HA-SUMO-1 and Ubc9, immunoprecipitated UL44-FLAG using an anti-FLAG antibody and analyzed the immunoprecipitated proteins by western blot. As expected, three slower migrating bands were detected by the anti-FLAG antibody from lysates of cells expressing UL44-FLAG in the presence of both HA-SUMO-1 and Ubc9 (Fig. 3B, left panel). The ∼50-kDa non-sumoylated form of UL44 could be immunoprecipitated both from cells expressing UL44-FLAG alone and from cells expressing UL44-FLAG together with HA-SUMO-1 and Ubc9, but not from cells expressing only HA-SUMO-1 and Ubc9. Slower migrating forms of UL44 could be detected by both the anti-FLAG and anti-HA antibody in the immunoprecipitation reaction of cells expressing UL44-FLAG in the presence of HA-SUMO and Ubc9, but not in immunoprecipitates obtained from cells expressing only UL44-FLAG (Fig. 3B, right panel). Similar results were obtained in HeLa cells (Fig. S2). Altogether, these results demonstrate that Ubc9 can mediate the conjugation of SUMO-1 to UL44 in mammalian cells. Moreover, sumoylation of UL44 in mammalian cells by both SUMO-2 and SUMO-3 could also be detected (Fig. S3).

Since sumoylation mainly occurs in the nucleus of mammalian cells [56] and UL44 is translocated to the host cell nucleus during HCMV infection [11], we decided to investigate whether nuclear localization might be a prerequisite for conjugation of SUMO-1 to UL44. We therefore analyzed the ability of UL44βΔNLS, a derivative of UL44 bearing point mutations within the nuclear localization signal (NLS, ^425^PNTKKQK^431^) which prevent UL44 nuclear accumulation [11], to be modified by SUMO-1. Interestingly, mutations of UL44 NLS impaired the sumoylation of a V5-UL44 fusion protein (V5-UL44ΔNLS, Fig. 3C), suggesting that SUMO-1 modification of UL44 most likely occurs into the nucleus or during nuclear import.

### Mapping of Sumoylation Sites in UL44

We next sought to identify the SUMO-1 acceptor sites of UL44. A prediction analysis with the SUMOplot program (Abgent) identified seven residues in UL44 with a certain probability to be sumoylated: K73, K172, K224, K339, K371, K410, and K431. To test whether one or more of these lysines could be a SUMO-1 acceptor site, each of the candidate residues was conservatively mutated to arginine, both individually and in combination, and the mutant proteins were tested for *in vitro* sumoylation. None of the single point mutants exhibited a consistently altered SUMO-1 modification pattern as compared to wild-type UL44 (Fig. S4A). Mutants carrying two or three K/R substitutions also showed a SUMO-1 modification pattern identical to that of wild-type UL44 (Fig. S4A and data not shown). Similar results were obtained when *in vitro* sumoylation reactions with SUMO-2/−3 were performed (data not shown). We decided to also test the ability of these K/R mutants to be modified by SUMO-1 in mammalian cells. Consistent with the *in vitro* data, none of the tested mutations significantly affected the ability of UL44 to undergo SUMO-1 conjugation (Fig. S4B and data not shown).

These results suggested that UL44 might possess multiple lysine residues that could alternatively serve as SUMO-1 acceptors, as reported for other proteins [57], [58], and/or that UL44 might be sumoylated on lysine residues other than those predicted by SUMOplot. UL44 contains 31 lysines, most of which are solvent-exposed in the crystal structure [9] and therefore potentially accessible to SUMO molecules, which makes it difficult to identify the target lysines by mutational approaches. Therefore, we attempted to map the sites of UL44 where SUMO-1 is conjugated by mass spectrometry analysis. To this end, we expressed UL44 in an *E. coli* expression/modification system that produces SUMO-conjugated proteins [30]. The 6His-tagged UL44 construct was co-expressed in *E. coli* with the pTE1E2S1 plasmid, which contains a linear fusion of genes for E1 and E2 enzymes and SUMO-1 under the control of an IPTG-inducible promoter [30]. To confirm sumoylation of UL44 with this system, UL44 was purified from the bacterial cultures expressing UL44 alone or in combination with the SUMO conjugation system and analyzed by western blotting with both the anti-UL44 (Fig. S5, left panel) and the anti-SUMO-1 (Fig. S5, right panel) antibody. Shifted bands with an apparent molecular mass of ∼65, 80, and 95 kDa, similar to those detected in *in vitro* reactions (Fig. 2A) and in cells (Fig. 3), were observed in UL44 purified from bacteria cotransformed with pTE1E2S1, but not in UL44 purified from bacteria expressing only UL44 (Fig. S5). Then, to identify lysine acceptor sites by mass spectrometry, sumoylated UL44 was separated by SDS-PAGE, in-gel-digested with trypsin and peptides were analyzed by LC-MSMS. Upon application of the software “ChopNSpice” for database search, we identified 16 sumoylation sites in UL44, including the predicted sites K172, K339, K371, K410, and K431. Table 2 summarizes the UL44 peptides that have been found to be sumoylated. The corresponding MS spectra are shown in Fig. S6.

**Table 2:** Sumoylation sites in UL44 as identified by mass spectrometry.

Exp. m/z	ChargeState	Exp. M.W.	Cal. M.W.	Delta	PeptideScore	Sequence	Position
1278.5924	2	2555.1703	2555.1697	0.0006	107.18	^4^ **K**TR^6^	K4
1400.6624	2	2799.3103	2799.316	−0.0057	96.35	^17^L**K**PYK^21^	K18
1277.6009	2	2553.1873	2553.1904	−0.0032	113.11	^166^V**K**R^168^	K167
1320.6197	2	2639.2249	2639.2272	−0.0023	114.38	^169^NV**K**K^172^	K171
1436.379	3	4306.1151	4306.1137	0.0014	95.68	^172^ **K**APCPTGTVQILVHAGPPAIK^192^	K172(*)
1114.8519	3	3341.5338	3341.518	0.0158	74.35	^209^VSFHGV**K**NMR^218^	K215
1217.2088	3	3648.6046	3648.605	−0.0004	79.21	^275^GDPFD**K**NYVGNSGK^288^	K280
1078.4899	3	3232.4478	3232.4466	0.0012	73.01	^281^NYVGNSG**K**SR^290^	K288
1354.1093	2	2706.204	2706.2079	−0.0039	111.75	^339^ **K**HDR^342^	K339(*)
1283.8971	3	3848.6696	3848.6741	−0.0046	61.49	^352^ **K**MSSGGGGGDHDHGLSSK^369^	K352
1033.1386	3	3096.3939	3096.3869	0.0069	52.16	^370^E**K**YEQHK^376^	K371(*)
1250.5829	3	3748.7269	3748.7301	−0.0033	56.68	^372^YEQH**K**ITSYLTSK^384^	K376
1407.3035	3	4218.8886	4218.9136	−0.025	64.86	^377^ITSYLTS**K**GGSGGGGGGGGGGLDR^400^	K384
1550.4359	4	6197.7145	6197.7244	−0.0098	85.24	^401^NSGNYFNDA**K**EESDSEDSVTFEFVPNTK^428^	K410(*)
1278.0902	2	2554.1658	2554.1745	−0.0087	106.9	^429^ **K**QK^431^	K429
1093.4843	3	3277.431	3277.4279	0.0031	74.38	^429^KQ**K**CGTEEF^437^	K431(*)

Tryptic peptides derived from UL44 conjugated to SUMO-1. The table lists the measured *m/z* values, the experimentally determined (Exp.) molecular weights (M.W.) in Da, the calculated (Cal.) M.W. in Da as determined by database search, and the mass difference between calculated and experimental M.W. in Da. The amino acid sequences are listed with their positions within UL44 protein. Underlined bold amino acid indicates the SUMO-1-conjugated lysine in UL44, that is also listed in the far right column. Amino acids are in one-letter code. Asterisks indicate that the specific sumoylation site was predicted with the SUMOplot software.

These results indicated that UL44 possesses multiple SUMO target lysines that are located throughout the protein, in accordance with the observation that Ubc9 could bind to both N- and C-terminal portions of UL44 (Fig. 1D and Fig. S1). Mutagenesis of several of these lysines in combination caused a strong decrease of UL44 expression (data not shown), likely due to protein misfolding and/or instability, making impossible to analyze the sumoylation state of the mutated protein and to compare it to that of wild-type UL44.

#### UL44 sumoylation is stimulated by binding to DNA

**Figure 4:**
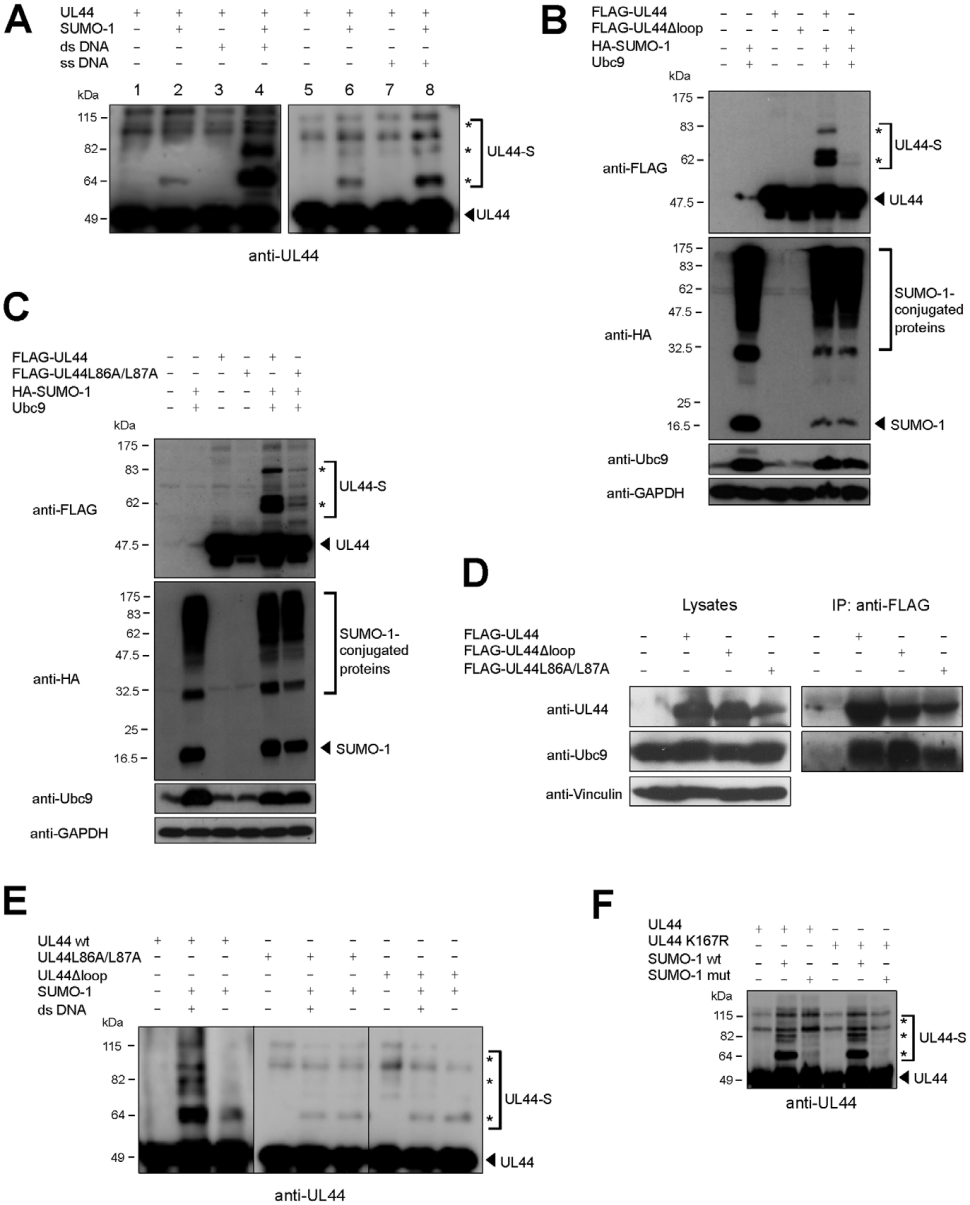
UL44 sumoylation is stimulated by DNA. (**A**) Purified 6His-UL44 protein was incubated with sumoylation proteins in the absence or presence of dsDNA (left panel) or ssDNA (right panel). Samples were analyzed by western blotting with an anti-UL44 antibody. (**B, C**) Phoenix cells were transfected to express wild-type UL44 or the FLAG-UL44Δloop or FLAG-UL44L86A/L87A mutant, which are defective for DNA binding. At 48 h post-transfection, cell lysates were analyzed by western blotting with anti-FLAG, anti-HA, anti-Ubc9, and anti-GAPDH antibodies. (**D**) Phoenix cells were transfected to express the indicated proteins. At 48 h post-transfection, cell lysates were analyzed by western blotting with anti-UL44, anti-Ubc9, and anti-vinculin antibodies (left panel). Cell lysates were incubated with anti-FLAG-M2-Agarose beads and the immunoprecipitated samples were analyzed by western blotting with anti-UL44 and anti-Ubc9 antibodies (right panel). (**E**) The sumoylation *in vitro* of wild-type 6His-UL44 and mutant 6His-UL44Δloop and 6His-UL44L86A/L87A proteins was carried out as in (A) and analyzed by western blotting with an anti-UL44 antibody. (**F**) The sumoylation *in vitro* of a UL44 mutant bearing the K167R substitution in the flexible loop of UL44 involved in DNA binding was carried out in the presence of DNA and compared to that of wild-type UL44. For all panels, the arrowhead indicates the unmodified form of UL44 or free SUMO-1 and the asterisks indicate the sumoylated forms.

As mentioned above, UL44 possesses a structural fold similar to that of the eukaryotic processivity factor PCNA [9]. In addition, like UL44, PCNA is SUMO-conjugated and its sumoylation involves both a consensus and a non-consensus site [4]. Since it has been shown that PCNA needs DNA to be sumoylated efficiently [59], we wished to investigate whether UL44 might behave similarly and its sumoylation could be stimulated by the presence of DNA. Thus, we performed *in vitro* sumoylation experiments in the absence and in the presence of DNA using as a substrate a purified 6His-UL44 fusion protein treated with polymin P to eliminate residual bacterial nucleic acids. In a western blot analysis with an anti-UL44 antibody (Fig. 4A, left panel), only a faint band corresponding to mono-sumoylated UL44 was observed in the absence of DNA (lane 2). Upon addition of dsDNA (e.g., activated calf thymus DNA) to the reaction mixture, a marked increase of the mono-sumoylated product and the appearance of bi- and tri-sumoylated forms of UL44 were observed (Fig. 4A, lane 4 of left panel). A western blot analysis with an anti-SUMO-1 antibody confirmed that these bands indeed contained SUMO-1 (data not shown). Similar results were obtained when GST-UL44 was used as a substrate (Fig. S7). Thus, like PCNA sumoylation, UL44 sumoylation is strongly stimulated by the presence of DNA.

To investigate whether the nature of DNA could influence the stimulation of UL44 sumoylation, we also performed *in vitro* sumoylation reactions in the presence of different DNA substrates. Similar stimulation levels were obtained when different dsDNAs were added, regardless of their sequence (data not shown), in keeping with previous observations that UL44 binds DNA in a sequence-independent manner [7]. In contrast, consistently less stimulation of UL44 sumoylation was observed in the presence of ssDNA (Fig. 4A, right panel), for which UL44 has been shown to possess an apparent affinity lower than for dsDNA [7]. This suggested that UL44 sumoylation could depend on binding to DNA. To test this hypothesis, we analyzed the ability of two UL44 mutants, FLAG-UL44Δloop and FLAG-UL44L86A/L87A, which are defective for DNA binding [36], to be modified by SUMO-1 in mammalian cells. FLAG-UL44Δloop contains three point mutations within a UL44 flexible loop (^163^HTRVKRNVKKAP^174^) involved in UL44-DNA interaction [8], [9], and is therefore impaired in DNA binding [36]. FLAG-UL44L86A/L87A carries two point mutations preventing dimerization of UL44 and strongly impairing the UL44-DNA interaction both *in vitro* and *in vivo* [9], [36], [60]. Both FLAG-UL44Δloop (Fig. 4B) and FLAG-UL44L86A/L87A (Fig. 4C), when co-expressed with HA-SUMO-1 and Ubc9, exhibited strongly reduced sumoylation levels when compared to FLAG-UL44. Importantly, co-immunoprecipitation experiments demonstrated that the reduced sumoylation of FLAG-UL44Δloop and FLAG-UL44L86A/L87A was not due to an impairment of binding of the mutant proteins to Ubc9, since the two mutants precipitated with endogenous Ubc9 at levels comparable to those of the wild-type protein (Fig. 4D). In addition, the presence of DNA did not stimulate the sumoylation of the UL44Δloop or UL44L86A/L87A mutants in *in vitro* reactions (Fig. 4E). Finally, since the UL44Δloop mutant contains a substitution (K167N) involving a potential SUMO target lysine, we wished to exclude the possibility that the reduced sumoylation levels of FLAG-UL44Δloop might be due to alteration of a putative SUMO acceptor site rather than an impairment of DNA binding. To this end, the K167 residue was conservatively mutated to arginine and the mutant protein was tested for *in vitro* sumoylation in the presence of DNA. The K167R mutant showed a SUMO-1 modification pattern identical to that of wild-type UL44 (Fig. 4F).

Altogether, these results suggest that UL44 is preferentially modified by SUMO-1 when it is bound to DNA as a dimer.

### UL44 is Sumoylated during the HCMV Replicative Cycle

**Figure 5:**
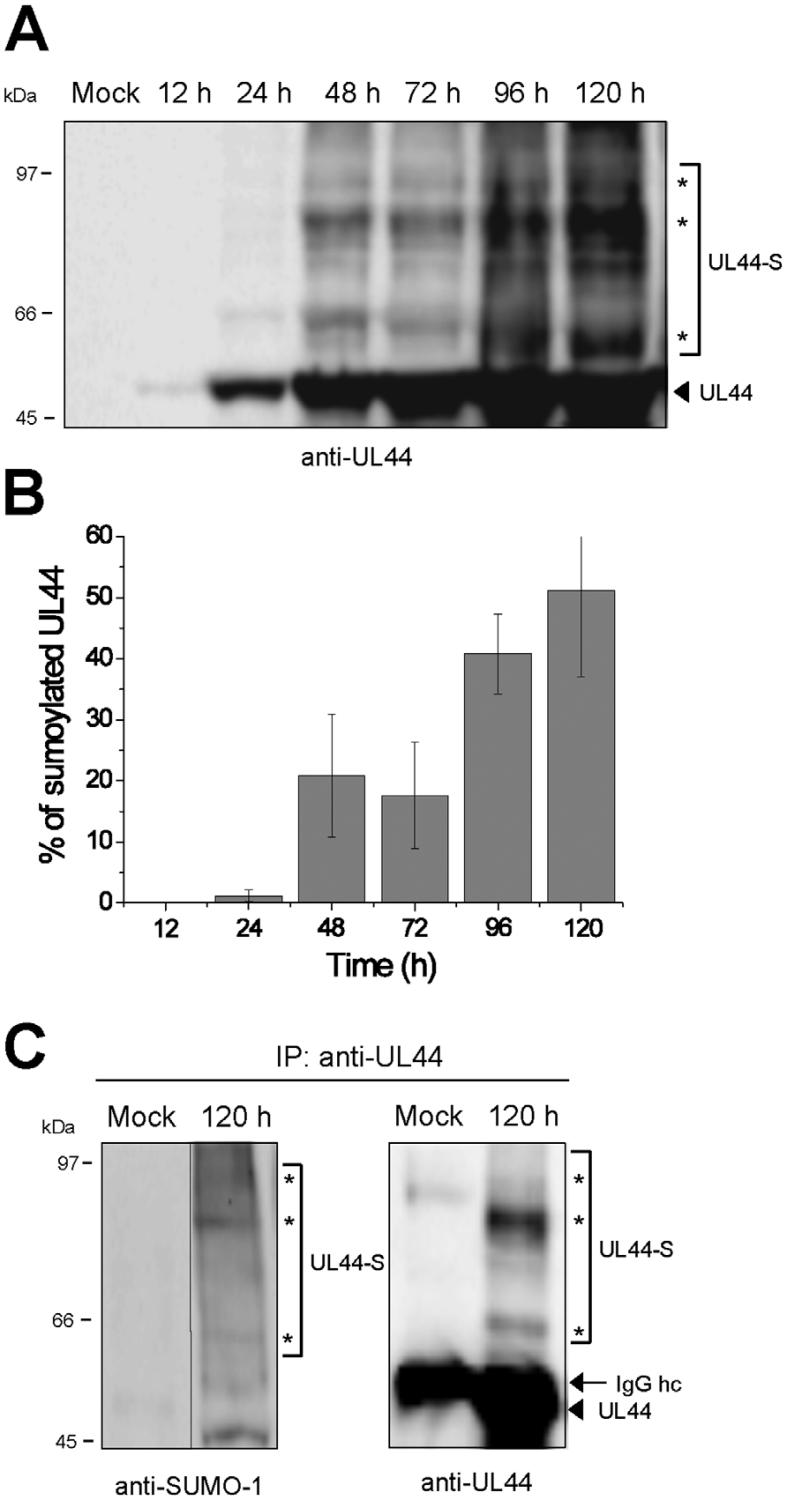
Sumoylation of UL44 in HCMV-infected cells. (**A**) HFFs were either mock-infected or infected with HCMV for the indicated times. Cell lysates were analyzed by western blotting with an anti-UL44 antibody. (**B**) Blots were analyzed by densitometry and the percentage of sumoylated UL44 bands relative to that of unmodified UL44 at each time p.i. was plotted versus the p.i. time point. Data represent the means ± standard deviations (error bars) of values from three independent experiments such as that shown in (A). (**C**) Lysates from either mock-infected or HCMV-infected HFF cells were prepared at 120 h p.i. and immunoprecipitated with an anti-UL44 antibody. Immunoprecipitates were analyzed by western blotting with anti-SUMO-1 (left panel) and anti-UL44 (right panel) antibodies. For all panels, the arrowhead indicates the unmodified form of UL44, the arrow indicates the immunoglobulin G heavy chain (IgG hc) and the asterisks indicate the sumoylated UL44 forms.

Having demonstrated that UL44 is sumoylated by Ubc9 *in vitro* and in transfected cells, we sought to investigate whether a similar modification also occurs naturally in HCMV-infected cells. Protein lysates of HFFs infected with HCMV and collected at different times post-infection (p.i.) were analyzed by western blotting with an anti-UL44 antibody. Two main bands of 65 and 80 kDa were observed above the primary UL44 band of 50 kDa during the whole time course of lytic infection, being detectable already at 24 h p.i. (Fig. 5A). A third band of ∼95 kDa was also visible from 48 h p.i. These subforms were similar in electrophoretic mobility to the UL44 bands covalently modified by SUMO-1 in transfected cells (Fig. 3A). A densitometric analysis of the protein bands (Fig. 5B) revealed that the relative amount of the sumoylated forms increased during the course of HCMV infection, becoming ∼50% of total UL44 protein at 120 h p.i.

To confirm that the slower migrating forms indeed represent UL44 molecules conjugated to SUMO-1, lysates from HCMV-infected cells at 120 h p.i. or from mock-infected cells were immunoprecipitated with an antibody against UL44. Subsequently, the immunocomplexes were analyzed by western blotting with an anti-SUMO-1 antibody. Three main bands of the expected molecular mass (∼65, 80, and 95 kDa) were recognized in the anti-UL44 immunoprecipitate (Fig. 5C, left panel). Finally, the same immunocomplexes were analyzed by western blotting with the anti-UL44 antibody to demonstrate that the SUMO-1 cross-reactive proteins were indeed modified UL44 forms (Fig. 5C, right panel).

Thus, these results clearly indicate that UL44 is covalently modified by SUMO-1 in HCMV-infected cells.

### Overexpression of SUMO-1 Alters the Intranuclear Distribution of UL44 during HCMV Replication and Promotes Virus Replication

**Figure 6:**
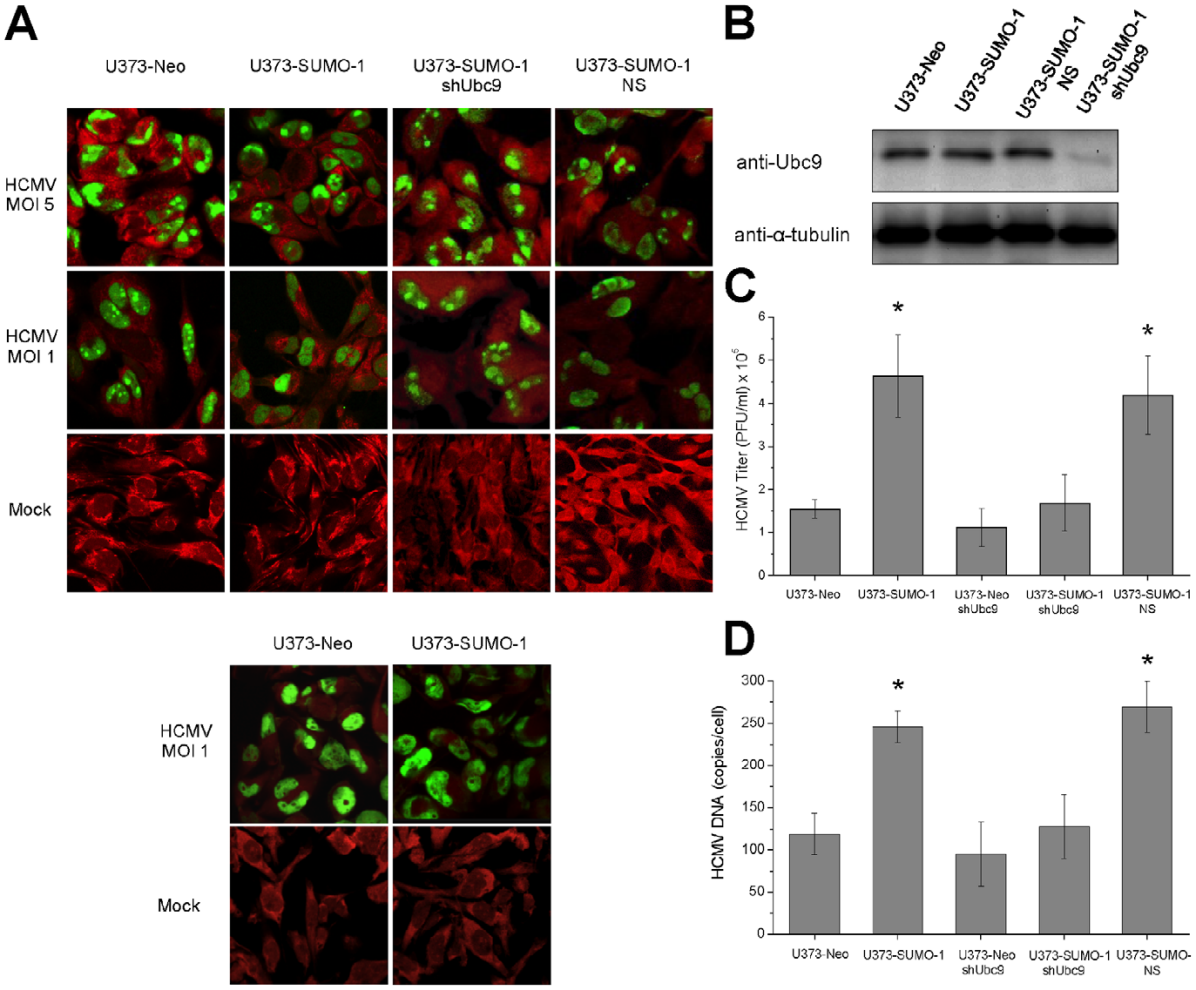
Overexpression of SUMO-1 modifies the subnuclear localization of UL44 and promotes virus replication in HCMV-infected cells. (A) U373 cells that constitutively overexpress SUMO-1 (U373-SUMO-1), control U373-Neo cells, and U373-SUMO-1 cells transduced with lentiviral particles expressing either a *Ubc9*-silencing shRNA (U373-SUMO-1 shUbc9) or a non-silencing shRNA sequence (U373-SUMO-1 NS) were mock-infected or infected with HCMV at an MOI of 5 or 1 PFU/cell. At 72 h p.i., cells were fixed and stained with a primary antibody against UL44 (upper panels) or against UL57 (lower panels), and successively with a secondary fluorescein-conjugated antibody (green) which contained Evans Blue to counterstain cells (red). Cell samples were then analyzed by CLSM. (B) Western blot analysis of Ubc9 expression in Ubc9-knocked-down and control cells. Cell lysates were prepared from the indicated cell lines and subjected to SDS-PAGE. Filter was then probed with anti-Ubc9 and anti-α-tubulin MAbs. (C) The indicated cell lines were infected with HCMV at an MOI of 1 and viral DNA levels were measured at 72 h p.i. by quantitative real-time PCR. (D) The indicated cell lines were infected with HCMV at an MOI of 1 and the titers of infectious virus progeny produced at 120 h p.i. were determined by plaque assays. In both (C) and (D), the data shown represent the means ± standard deviations (error bars) of two independent experiments performed in duplicate or triplicate. The asterisks denote a statistically significant difference (*P<*0.05) between the values relative to U373-SUMO-1 and U373-SUMO-1 NS and the values relative to control U373-Neo cells.

Considering the difficulties in expressing a UL44 mutant completely impaired in sumoylation, whose activities could be compared to that of wild-type UL44, to gain some insight on the functional role of UL44 sumoylation in the context of HCMV replication we sought to undertake a different approach. We overexpressed SUMO-1 in virus-infected cells and analyzed the effects on the intracellular distribution of UL44, as the targeting to specific subcellular domains is one of most common biological effect exerted by the conjugation of SUMO to a substrate protein. It has been previously shown that during HCMV replication UL44 localizes to large globular intranuclear structures that correspond to viral DNA replication compartments [61], [62], [63]. A U373-MG cell line that constitutively overexpresses FLAG-SUMO-1 and control U373-Neo cells were mock-infected or infected with HCMV at an MOI of 1 or of 5 PFU/cell and the intracellular localization of UL44 was successively analyzed by indirect immunofluorescence with an anti-UL44 antibody. Control western blotting experiments (Fig. S8A) confirmed that UL44 is sumoylated in the HCMV-infected U373 cells. In fact, slower migrating bands of the expected molecular mass and similar to the UL44 sumoylated forms observed in infected HFFs (Fig. 5A) were detected. Furthermore, as expected, they increased upon SUMO-1 overexpression. In immunofluorescence assays, the nuclei of mock-infected cells were oval-shaped with no anti-UL44 staining (Fig. 6A, upper panels). Control HCMV-infected U373-Neo cells showed deformed nuclei, many of which exhibited a kidney shape (Fig. 6A, upper panels). Indeed, it has been observed that infection by HCMV causes this kind of distortions in nuclear shape [64], [65]. Moreover, UL44 showed a globular fluorescent pattern consistent with previously described viral replication compartments in HCMV-infected cells [61], [66]. In contrast, in HCMV-infected U373-SUMO-1 cells UL44 staining was more distributed throughout the nucleus and, especially at the lower MOI (MOI 1), also failed to coalesce into any recognizable globular structures (Fig. 6A, upper panels). Therefore, overexpression of SUMO-1 during HCMV replication appears to alter the intranuclear distribution of UL44, likely leading to significantly decreased localization of UL44 in viral DNA replication compartments. Importantly, the intranuclear distribution of another HCMV protein localizing to the replication compartments, i.e. UL57, the single-stranded DNA (ssDNA)-binding protein [61], [67], appeared not to change upon SUMO-1 overexpression (Fig. 6A, lower panels).

To investigate whether sumoylation is indeed involved in the altered intranuclear distribution of UL44, an RNAi approach was employed to suppress the sumoylation system of the cells by silencing Ubc9 since Ubc9 is the only unique and essential enzyme in the SUMO-conjugating pathway [21]. U373-Neo and U373-SUMO-1 cells were transduced with a lentivirus expressing shUbc9, followed by selection with puromycin and induction with doxycyline to establish Ubc9-knocked-down cell lines (U373-Neo shUbc9 and U373-SUMO-1 shUbc9, respectively). As a control, U373-SUMO-1 cells were also transduced with a lentivirus expressing a non-silencing shRNA sequence (U373-SUMO-1 NS). As shown in Figure 6B, Ubc9 was almost completely silenced in cells infected with the shUbc9 lentivirus (U373-SUMO-1 shUbc9) as compared to the cells transduced with the control lentivirus (U373-SUMO-1 NS) and to non-transduced cells (U373-Neo and U373-SUMO-1). To examine the effect of Ubc9 knock-down on intranuclear distribution of UL44, these cell lines were infected with HCMV. As shown in Fig. 6A (upper panels), upon doxycycline induction, the nuclear UL44 staining in Ubc9-knocked-down U373-SUMO-1 cells was similar to that of U373 cells not overexpressing SUMO-1 (U373-Neo and U373-Neo shUbc9; Fig. 6A, upper panels, and Fig. S8B). In contrast, the U373-SUMO-1 cells transduced with the non-silencing lentivirus (U373-SUMO-1 NS, Fig. 6A, upper panels) retained an altered intranuclear distribution of UL44 similar to that observed in the non-transduced cells (U373-SUMO-1) or in the shUbc9-transduced U373-SUMO-1 cells with no doxycycline induction (data not shown). Altogether, these results established that the altered intranuclear distribution of UL44 in HCMV-infected cells upon SUMO-1 overexpression depends on Ubc9-mediated sumoylation.

These observations raised the question whether the altered intranuclear distribution of UL44 observed upon SUMO-1 overexpression affected the viral replication efficiency. To examine the effects of SUMO-1 overexpression on viral DNA replication, U373-Neo and U373-SUMO-1 cells were infected with HCMV at an MOI of 1 and viral DNA levels were measured by quantitative real-time PCR. Viral DNA production from U373-SUMO-1 cells was ∼two-fold higher than that from the control cells (U373-Neo and U373-Neo shUbc9; Fig. 6C). This increase was not observed in Ubc9-knocked down cells (U373-SUMO-1 shUbc9), while the U373-SUMO-1 cells transduced with the non-silencing lentivirus (U373-SUMO-1 NS) exhibited augmented viral DNA levels like the non-transduced U373-SUMO-1 cells (Fig. 6C).

We also examined the effects of SUMO-1 overexpression on virus yield. The titers of viral particles produced from non-transduced U373-SUMO-1 and from transduced U373-SUMO-1 shUbc9 and U373-SUMO-1 NS cells after infection with HCMV at an MOI of 1 were determined and compared to those produced from infected U373-Neo and U373-Neo shUbc9 control cells. A 2-3-fold increase in viral progeny titers was observed in U373-SUMO-1 and U373-SUMO-1 NS with respect to U373-Neo, while the U373-SUMO-1 shUbc9 cells exhibited yields of infectious virus similar to those of the U373-Neo and U373-Neo shUbc9 cells (Fig. 6D).

Thus, the altered intranuclear distribution of UL44 upon SUMO-1 overexpression appears not to compromise HCMV replication, but conversely, SUMO-1 overexpression causes a positive effect on virus production.

## Discussion

In this study we report that UL44, a viral ortholog of PCNA, is sumoylated on multiple lysines by the cellular factor Ubc9. Importantly, a consistent portion of UL44 is SUMO-modified in HCMV-infected human cells, resulting in ∼50% of the protein being modified at late times during virus replication. From a structural point and functional of view, UL44 and PCNA share some remarkable similarities and some differences. Monomers of UL44 and PCNA are structurally very similar, despite having extremely different primary sequences [2], [9]. However, while PCNA forms toroidal-homotrimers, UL44 binds to dsDNA as a head-to-head homodimer [7], [9]. In addition, PCNA must be loaded onto DNA in an ATP-dependent process by so-called clamp loaders [68]; in contrast, UL44 directly binds DNA without the need for ATP hydrolysis or accessory proteins [7], [14], [18].

Similarities and differences also emerge from the comparison of the sumoylation processes of UL44 and PCNA. The most striking similarity is the DNA-dependence of such post-translational modification: in the case of PCNA, clamp loading rather than the mere presence of DNA was shown to be important for stimulation, implying a change in the properties of PCNA upon loading that enhances its capacity to be sumoylated [59]. This could also be the case for UL44: in fact, point mutations preventing DNA binding [8], [9], [36], [60] strongly impaired UL44 sumoylation in cells (Fig. 4C) and abolished the ability of dsDNA to stimulate UL44 sumoylation *in vitro* (Fig. 4E). Furthermore, the ability of DNA to stimulate UL44 sumoylation appears to correlate with the efficiency of the UL44-DNA interaction. In fact, addition of dsDNA -for which UL44 has a ∼3- to 8-fold higher affinity than for ssDNA [7]- caused a much stronger increase of UL44 sumoylation as compared to ssDNA (Fig. 4A). In this context, it is important to note that point mutations impairing DNA binding and sumoylation in cells did not compromise the UL44-Ubc9 interaction (Fig. 4D), suggesting that binding to DNA does not promote UL44 sumoylation by facilitating its binding to Ubc9.

Another similarity between UL44 and PCNA sumoylation is that both proteins are modified on canonical (K127 for PCNA, and K410 for UL44) and non-canonical residues (K164 for PCNA, and all other sumoylation sites for UL44, see Table 2). However, while PCNA is exclusively sumoylated at the N-terminus, both N- and C-terminal residues of UL44 can be modified. In addition, according to our MS-analysis UL44 can be alternatively sumoylated at 16 different sites, while K127 and K164 appear to be the only target sites in PCNA. This flexibility of UL44 in terms of sumoylation target sites, which is reminiscent of the ones described for Daxx and the small hepatitis delta antigen [57], [58], is arguably the main difference from PCNA. This makes it extremely difficult to study the physiological importance of UL44 sumoylation, also because mutation of several of these lysines caused protein instability. Currently it is therefore impossible to test if, like in the case of budding yeast PCNA, SUMO-modification of UL44 also plays a role in DNA repair/recombination [69].

In terms of functional effects, sumoylation is known to regulate protein activity and/or intracellular location [21], [22]. As for the latter, the targeting to specific subcellular domains is one of the best-characterized biological effects exerted by the conjugation of SUMO to a substrate protein. This effect is exemplified by the targeting of cellular protein RanGAP1 to the cytosolic side of the nuclear pore complex upon sumoylation [54], [70]. As a first, preliminary attempt to characterize the role of UL44’s sumoylation, here we show that overexpression of SUMO-1 in the context of HCMV replication alters the intranuclear distribution of UL44 as it appears to result in a more diffuse pattern and in decreased localization of UL44 in viral DNA replication compartments (Fig. 6A), suggesting that sumoylation of UL44 may retarget the protein to other nuclear site(s). Importantly, Ubc9 knock-down studies confirmed that sumoylation is responsible for such altered intranuclear pattern of UL44. The observation that SUMO-1 overexpression causes a positive effect on HCMV replication suggests that sumoylation of UL44 could be important for its function(s) in the context of the virus life cycle, although effects on HCMV replication mediated by sumoylation of other viral or cellular proteins cannot be excluded. However, at the moment understanding the molecular details of how SUMO alters the intranuclear distribution of UL44 is rather difficult. Most likely, sumoylation does not affect the functions of UL44 as a DNA polymerase processivity factor - the only role currently well established for UL44. In fact, several reports have shown that the UL44 protein expressed in *E. coli*, which is non-sumoylated, is capable of performing all known biochemical activities related to this role (e.g., [7], [14]). In addition, our observation that UL44 sumoylation peaks at late times during virus replication, once the viral DNA replication has been accomplished (Fig. 5), suggests that SUMO-conjugation might enable UL44 to fulfill some role(s) in HCMV replication other than that of conferring processivity to the viral DNA polymerase. A role of UL44 in late gene expression has been previously suggested [71], [72]; however, no conclusive demonstration has been provided yet.

Intriguingly, higher molecular mass forms of the Epstein-Barr virus DNA polymerase processivity factor BMRF1 compatible with sumoylated products have been observed [73], suggesting that such post-translational modification could be a general feature of the DNA polymerase accessory subunit in herpesviruses. In addition, sumoylation of Vaccinia Virus G8R protein has been recently predicted on the basis of structural similarities to PCNA [74], but not yet experimentally demonstrated. Thus, our findings could stimulate further studies on sumoylation of DNA polymerase subunits in other herpesviruses or, more in general, in other viral systems.

## Supporting Information

Figure S1 **Both N-terminal and C-terminal regions of UL44 bind endogenous Ubc9 in mammalian cells.** Phoenix cells were transfected to express full-length UL44 or truncated UL44 mutants. (**A**) At 48 h post-transfection, cell lysates were analyzed by western blotting with anti-FLAG, anti-Ubc9, and anti-GAPDH antibodies. (**B**) Cell lysates were incubated with anti-FLAG-M2-Agarose beads and the immunoprecipitated samples were analyzed by western blotting with anti-FLAG and anti-Ubc9 antibodies.
(TIF)

Figure S2 **UL44 is sumoylated in HeLa cells.** HeLa cells were transfected to express the indicated proteins. (**A**) At 48 h post-transfection, cell lysates were analyzed by western blotting with anti-FLAG, anti-HA, anti-Ubc9, and anti-α-tubulin antibodies. (**B**) Cell lysates were incubated with the anti-FLAG antibody and the immunoprecipitated samples were analyzed by western blotting with anti-HA and anti-FLAG antibodies. For all panels, the arrowhead indicates the unmodified form of UL44 or free SUMO-1 and the asterisks indicate the sumoylated forms.
(TIF)

Figure S3 **Sumoylation of UL44 by SUMO-2/−3 in mammalian cells.** Phoenix cells were transfected to express the indicated proteins. At 48 h post-transfection, cell lysates were analyzed by western blotting with anti-UL44, anti-HA, anti-Ubc9, and anti-vinculin antibodies (left panel). Cell lysates were incubated with anti-FLAG-M2-Agarose beads and the immunoprecipitated samples were analyzed by western blotting with anti-HA and anti-FLAG antibodies (right panel). For all panels, the arrowhead indicates the unmodified form of UL44 or free SUMO-2/−3 and the asterisks indicate the UL44 sumoylated forms.
(TIF)

Figure S4 **Mutational analysis of predicted SUMO-1 target sites of UL44.** **(A)** Lysine substitution mutants of UL44 were produced and analyzed for sumoylation *in vitro*. (**B**) Phoenix cells were transfected to express the indicated proteins and analyzed by western blotting with anti-FLAG, anti-HA, anti-Ubc9, and anti-GAPDH antibodies. For all panels, the arrowhead indicates the unmodified form of UL44 or free SUMO-1 and the asterisks indicate the sumoylated forms.
(TIF)

Figure S5 **Sumoylation of UL44 in** ***E. coli*** **.** The pRSET44 plasmid, encoding 6His-tagged UL44, was introduced into *E. coli* together with the pTE1E2S1 plasmid, which expresses E1 and E2 sumoylation enzymes and SUMO-1. As a control, bacteria were also transformed only with pRSET44. The 6His-tagged UL44 was purified from bacterial cultures expressing UL44 alone or in combination with the SUMO conjugation system and analyzed by western blotting with anti-UL44 and anti-SUMO-1 antibodies.
(TIF)

Figure S6 **Mass spectrometry analysis of sumoylation sites of UL44.** MSMS analysis of tryptic peptides conjugated to a tryptic peptide of SUMO-1 (ELGMEEEDVIEVYQEQTGG or IADNHTPKELGMEEEDVIEVYQEQTGG, 1 tryptic miscleavage) derived from *E. coli*-expressed, sumoylated UL44. Sequence of the conjugate is listed in each table above the spectra. Y-type and b-type fragment ions are assigned in the spectra. The table below each spectrum summarizes the database search. Highlighted are the *m/z* values that match the fragment ions obtained from *in silico* fragmentation of the conjugate. Conjugated lysine residues to SUMO-1 are highlighted in red and the position in UL44 is listed. Modifications, measured (observed) *m/z* values, the actual mass (in Da), the charge state, and the mass error (ppm, parts per million) are listed as well in the table above each spectrum. The different colors represent the various measured and calculated fragment ions of each conjugate in the spectrum and table underneath, respectively. The question marks in some of the spectra indicate fragment ions that do not match to any calculated y- and b-type ions of the conjugate.
(PDF)

Figure S7 **Sumoylation** ***in vitro*** **of a GST-UL44 fusion is stimulated by DNA.** *E. coli-*expressed, purified GST-tagged UL44 was incubated with purified sumoylation enzymes in the absence or presence of SUMO-1 and/or DNA. The samples were analyzed by western blotting with an anti-UL44 antibody.
(TIF)

Figure S8 **UL44 is sumoylated in HCMV-infected U373 cells.** (**A**) U373-Neo and U373-SUMO-1 cells were either mock-infected or infected with HCMV at MOI of 5 PFU/cell for 72 h. Cell lysates were then analyzed by western blotting with an anti-UL44 antibody. The arrowhead indicates the unmodified form of UL44 and the asterisks indicate the sumoylated UL44 forms. (**B**) Control U373-Neo cells, and U373-Neo cells transduced with lentiviral particles expressing either a *Ubc9*-silencing shRNA (U373-Neo shUbc9) or a non-silencing shRNA sequence (U373-Neo NS) were mock-infected or infected with HCMV at an MOI of 5 or 1 PFU/cell. At 72 h p.i., cells were fixed and stained with a primary antibody against UL44 and successively with a secondary fluorescein-conjugated antibody (green) which contained Evans Blue to counterstain cells (red). Cell samples were then analyzed by CLSM.
(TIF)

Table S1 Oligonucleotides used in this work for cloning and mutagenesis.
(DOC)

Text S1 Supplementary Material and Methods and Supplementary References.
(DOC)
