# Supplementary figures and images for: The Human Cytomegalovirus DNA Polymerase Processivity Factor UL44 Is Modified by SUMO in a DNA-Dependent Manner
Source: PLoS One. 2012 Nov 15;7(11):e49630. doi: 10.1371/journal.pone.0049630 (PMC3499415; doi:10.1371/journal.pone.0049630)

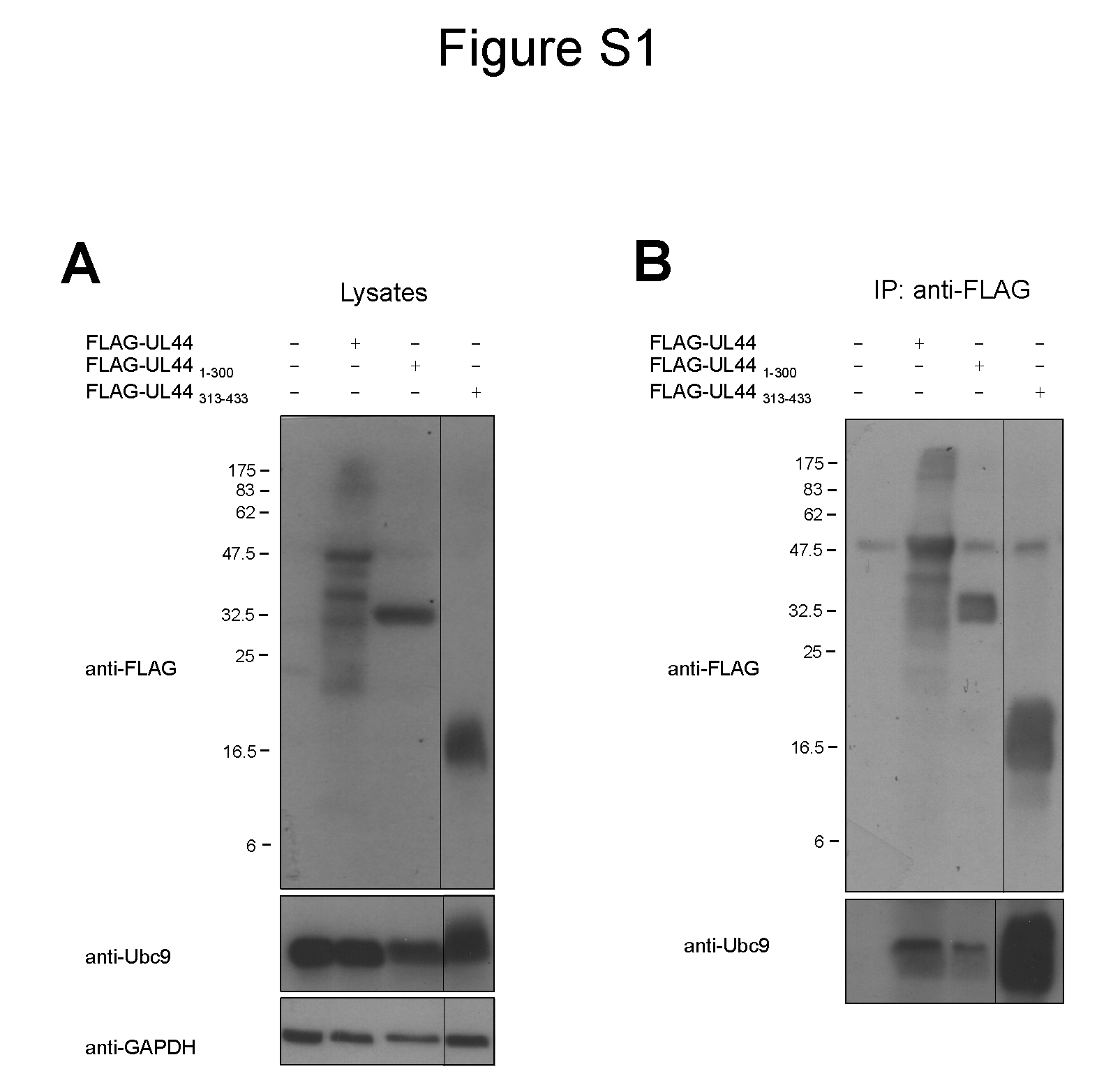

Supplement: Figure S1 — Both N-terminal and C-terminal regions of UL44 bind endogenous Ubc9 in mammalian cells. Phoenix cells were transfected to express full-length UL44 or truncated UL44 mutants. (A) At 48 h post-transfection, cell lysates were analyzed by western blotting with anti-FLAG, anti-Ubc9, and anti-GAPDH antibodies. (B) Cell lysates were incubated with anti-FLAG-M2-Agarose beads and the immunoprecipitated samples were analyzed by western blotting with anti-FLAG and anti-Ubc9 antibodies. (TIF) [file pone.0049630.s001.tif]

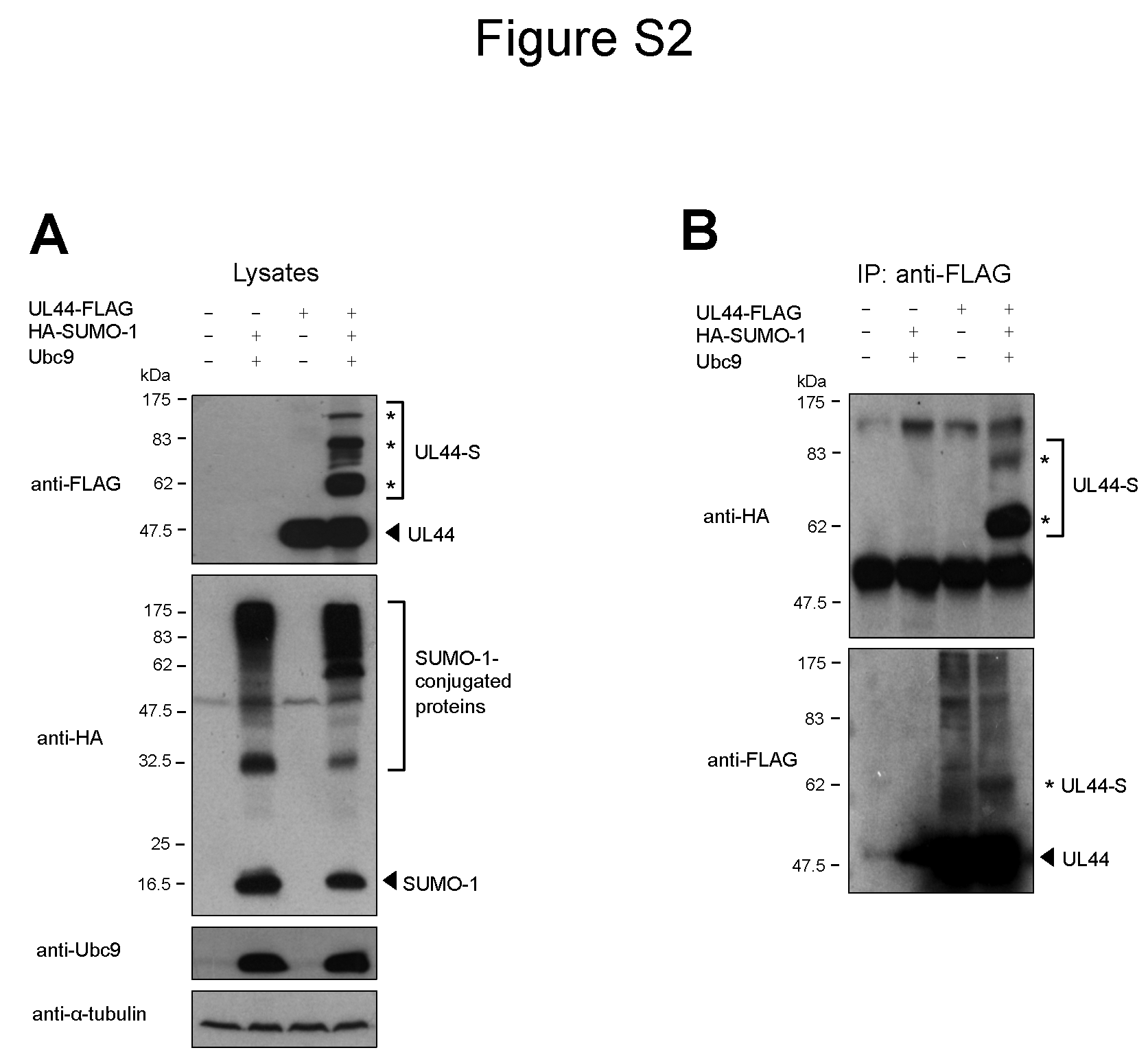

Supplement: Figure S2 — UL44 is sumoylated in HeLa cells. HeLa cells were transfected to express the indicated proteins. (A) At 48 h post-transfection, cell lysates were analyzed by western blotting with anti-FLAG, anti-HA, anti-Ubc9, and anti-α-tubulin antibodies. (B) Cell lysates were incubated with the anti-FLAG antibody and the immunoprecipitated samples were analyzed by western blotting with anti-HA and anti-FLAG antibodies. For all panels, the arrowhead indicates the unmodified form of UL44 or free SUMO-1 and the asterisks indicate the sumoylated forms. (TIF) [file pone.0049630.s002.tif]

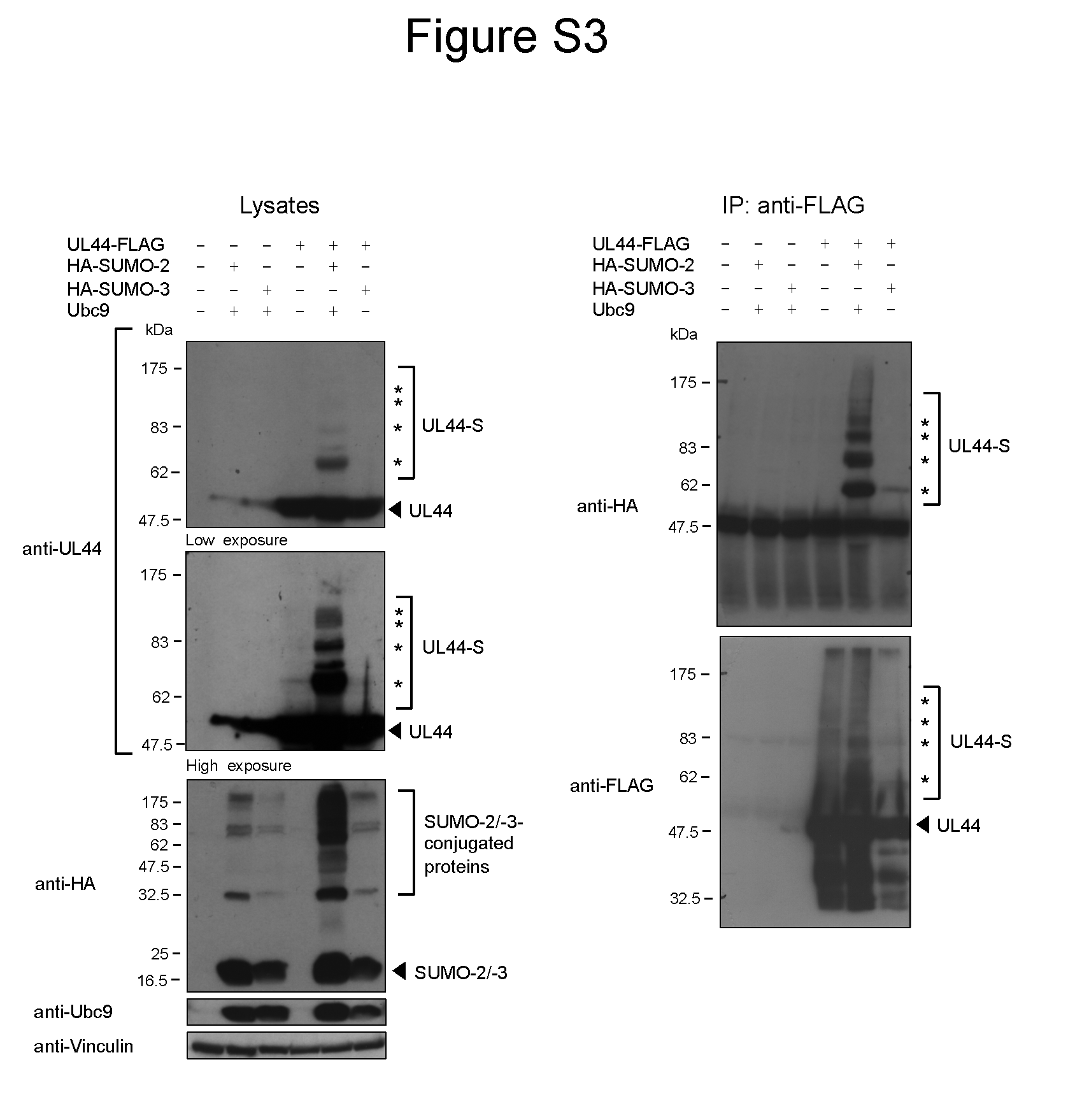

Supplement: Figure S3 — Sumoylation of UL44 by SUMO-2/−3 in mammalian cells. Phoenix cells were transfected to express the indicated proteins. At 48 h post-transfection, cell lysates were analyzed by western blotting with anti-UL44, anti-HA, anti-Ubc9, and anti-vinculin antibodies (left panel). Cell lysates were incubated with anti-FLAG-M2-Agarose beads and the immunoprecipitated samples were analyzed by western blotting with anti-HA and anti-FLAG antibodies (right panel). For all panels, the arrowhead indicates the unmodified form of UL44 or free SUMO-2/−3 and the asterisks indicate the UL44 sumoylated forms. (TIF) [file pone.0049630.s003.tif]

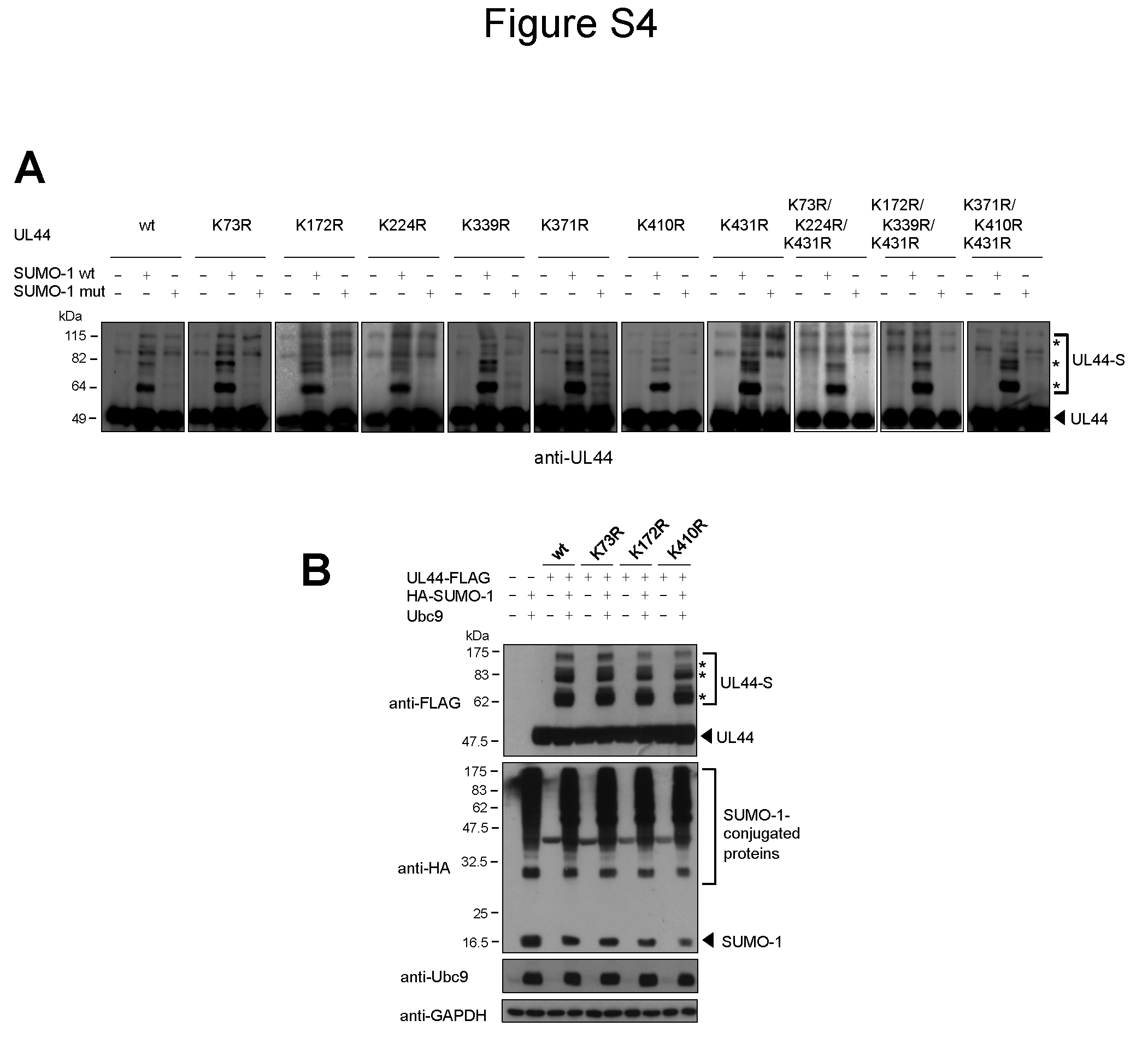

Supplement: Figure S4 — Mutational analysis of predicted SUMO-1 target sites of UL44. (A) Lysine substitution mutants of UL44 were produced and analyzed for sumoylation in vitro. (B) Phoenix cells were transfected to express the indicated proteins and analyzed by western blotting with anti-FLAG, anti-HA, anti-Ubc9, and anti-GAPDH antibodies. For all panels, the arrowhead indicates the unmodified form of UL44 or free SUMO-1 and the asterisks indicate the sumoylated forms. (TIF) [file pone.0049630.s004.tif]

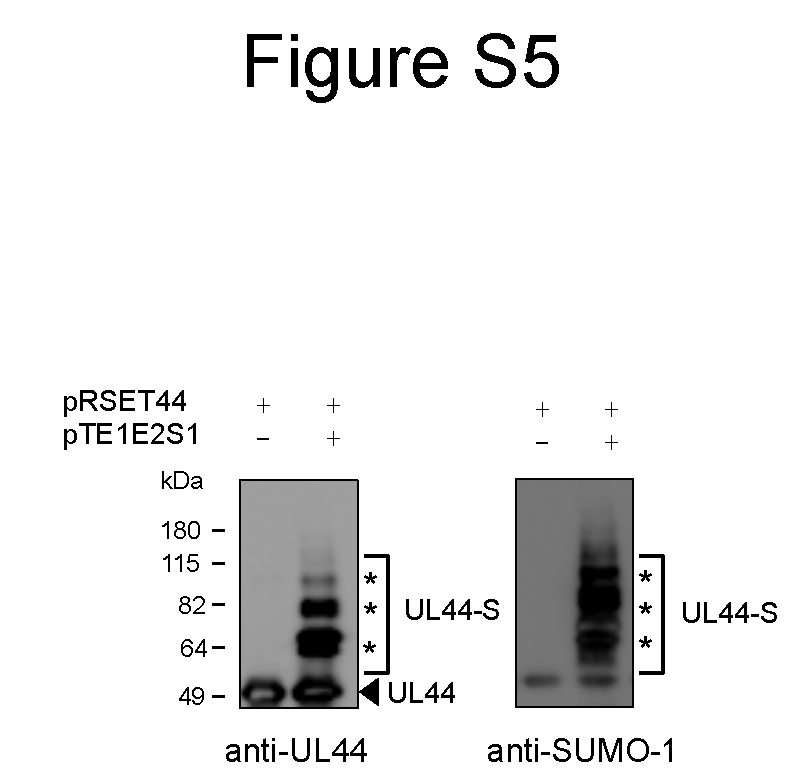

Supplement: Figure S5 — Sumoylation of UL44 in E. coli . The pRSET44 plasmid, encoding 6His-tagged UL44, was introduced into E. coli together with the pTE1E2S1 plasmid, which expresses E1 and E2 sumoylation enzymes and SUMO-1. As a control, bacteria were also transformed only with pRSET44. The 6His-tagged UL44 was purified from bacterial cultures expressing UL44 alone or in combination with the SUMO conjugation system and analyzed by western blotting with anti-UL44 and anti-SUMO-1 antibodies. (TIF) [file pone.0049630.s005.tif]

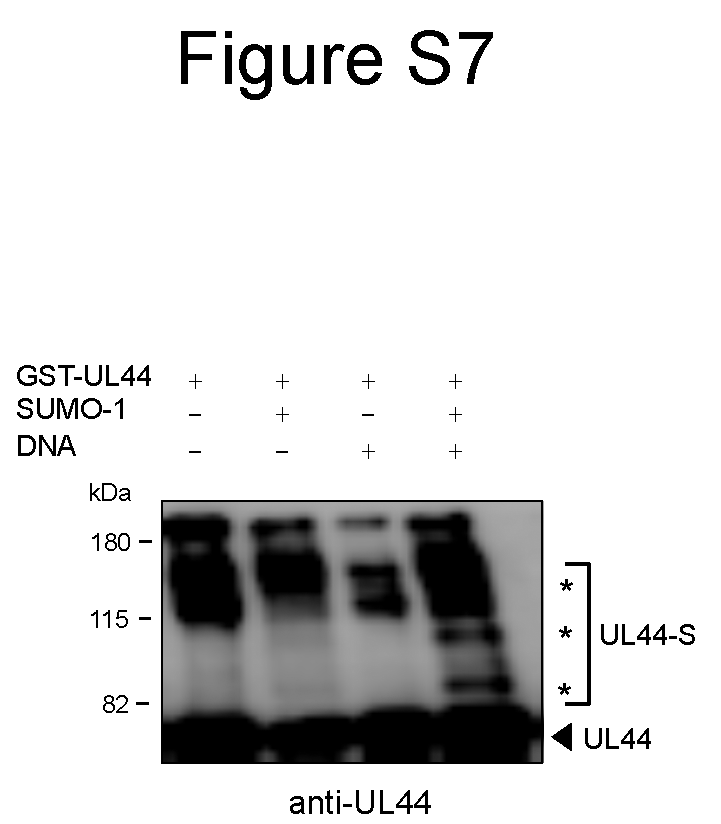

Supplement: Figure S7 — Sumoylation in vitro of a GST-UL44 fusion is stimulated by DNA. E. coli-expressed, purified GST-tagged UL44 was incubated with purified sumoylation enzymes in the absence or presence of SUMO-1 and/or DNA. The samples were analyzed by western blotting with an anti-UL44 antibody. (TIF) [file pone.0049630.s007.tif]

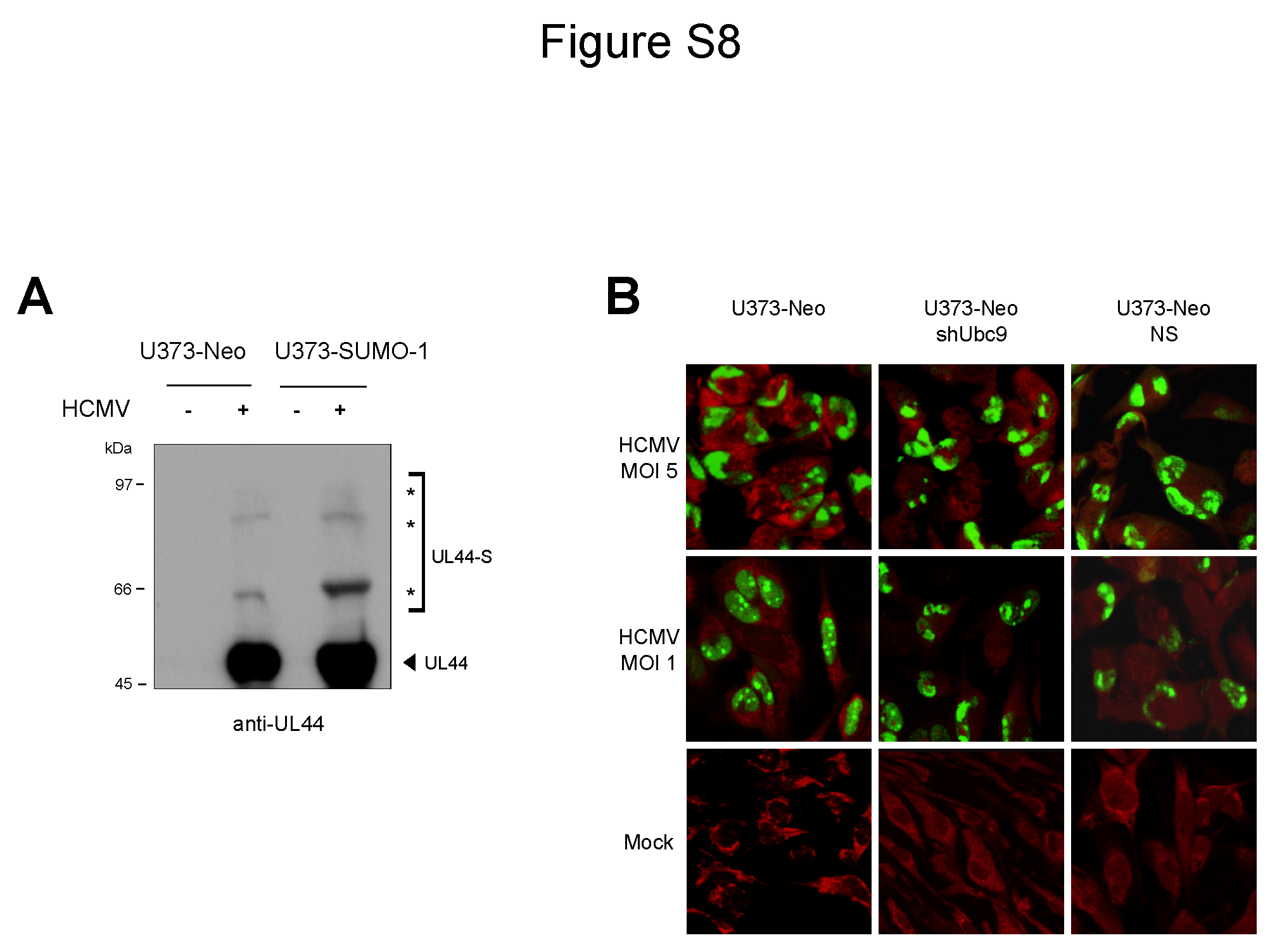

Supplement: Figure S8 — UL44 is sumoylated in HCMV-infected U373 cells. (A) U373-Neo and U373-SUMO-1 cells were either mock-infected or infected with HCMV at MOI of 5 PFU/cell for 72 h. Cell lysates were then analyzed by western blotting with an anti-UL44 antibody. The arrowhead indicates the unmodified form of UL44 and the asterisks indicate the sumoylated UL44 forms. (B) Control U373-Neo cells, and U373-Neo cells transduced with lentiviral particles expressing either a Ubc9-silencing shRNA (U373-Neo shUbc9) or a non-silencing shRNA sequence (U373-Neo NS) were mock-infected or infected with HCMV at an MOI of 5 or 1 PFU/cell. At 72 h p.i., cells were fixed and stained with a primary antibody against UL44 and successively with a secondary fluorescein-conjugated antibody (green) which contained Evans Blue to counterstain cells (red). Cell samples were then analyzed by CLSM. (TIF) [file pone.0049630.s008.tif]
