# Supplementary material for: The Human Cytomegalovirus DNA Polymerase Processivity Factor UL44 Is Modified by SUMO in a DNA-Dependent Manner
Source: PLoS One. 2012 Nov 15;7(11):e49630. doi: 10.1371/journal.pone.0049630 (PMC3499415; doi:10.1371/journal.pone.0049630)
Supplement: Figure S6 — Mass spectrometry analysis of sumoylation sites of UL44. MSMS analysis of tryptic peptides conjugated to a tryptic peptide of SUMO-1 (ELGMEEEDVIEVYQEQTGG or IADNHTPKELGMEEEDVIEVYQEQTGG, 1 tryptic miscleavage) derived from E. coli-expressed, sumoylated UL44. Sequence of the conjugate is listed in each table above the spectra. Y-type and b-type fragment ions are assigned in the spectra. The table below each spectrum summarizes the database search. Highlighted are the m/z values that match the fragment ions obtained from in silico fragmentation of the conjugate. Conjugated lysine residues to SUMO-1 are highlighted in red and the position in UL44 is listed. Modifications, measured (observed) m/z values, the actual mass (in Da), the charge state, and the mass error (ppm, parts per million) are listed as well in the table above each spectrum. The different colors represent the various measured and calculated fragment ions of each conjugate in the spectrum and table underneath, respectively. The question marks in some of the spectra indicate fragment ions that do not match to any calculated y- and b-type ions of the conjugate. (PDF) [file pone.0049630.s006.pdf]

Figure S6

Sequence	Mascot Score	Modification	Observed (m/z)	Actual Mass	Charge	Error (ppm)	Position
(-)ELGmEEEDVIEVYQEQTGG-**K**TR(-)	107.18	Oxidation (M)	1278.5924	2555.1702	2	0.08	K4

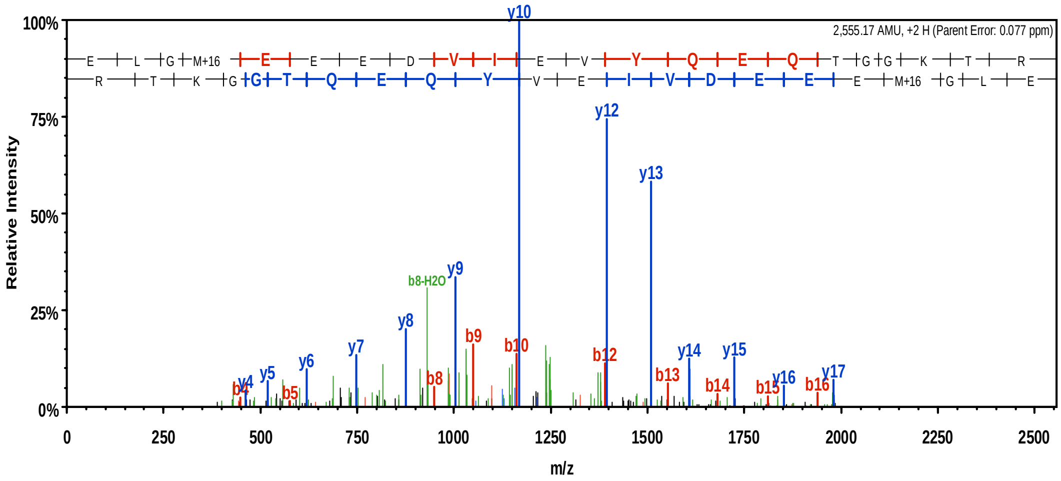

B	B Ions	B+2H	B-NH3	B-H2O	AA	Y Ions	Y+2H	Y-NH3	Y-H2O	Y
1	130.0			112.0	E	2,556.2	1,278.6	2,539.2	2,538.2	22
2	243.1			225.1	L	2,427.1	1,214.1	2,410.1	2,409.1	21
3	300.2			282.1	G	2,314.1	1,157.5	2,297.0	2,296.0	20
4	447.2			429.2	M+16	2,257.0	1,129.0	2,240.0	2,239.0	19
5	576.2			558.2	E	2,110.0	1,055.5	2,093.0	2,092.0	18
6	705.3	353.1		687.3	E	1,981.0	991.0	1,963.9	1,962.9	17
7	834.3	417.7		816.3	E	1,851.9	926.5	1,834.9	1,833.9	16
8	949.3	475.2		931.3	D	1,722.9	861.9	1,705.8	1,704.9	15
9	1,048.4	524.7		1,030.4	V	1,607.8	804.4	1,590.8	1,589.8	14
10	1,161.5	581.3		1,143.5	I	1,508.8	754.9	1,491.7	1,490.8	13
11	1,290.5	645.8		1,272.5	E	1,395.7	698.3	1,378.7	1,377.7	12
12	1,389.6	695.3		1,371.6	V	1,266.6	633.8	1,249.6	1,248.6	11
13	1,552.7	776.8		1,534.7	Y	1,167.6	584.3	1,150.5	1,149.6	10
14	1,680.7	840.9	1,663.7	1,662.7	Q	1,004.5	502.8	987.5	986.5	9
15	1,809.8	905.4	1,792.7	1,791.8	E	876.5	438.7	859.4	858.4	8
16	1,937.8	969.4	1,920.8	1,919.8	Q	747.4	374.2	730.4	729.4	7
17	2,038.9	1,019.9	2,021.9	2,020.9	T	619.4	310.2	602.3	601.3	6
18	2,095.9	1,048.5	2,078.9	2,077.9	G	518.3	259.7	501.3	500.3	5
19	2,152.9	1,077.0	2,135.9	2,134.9	G	461.3	231.1	444.3	443.3	4
20	2,281.0	1,141.0	2,264.0	2,263.0	K	404.3	202.6	387.2	386.3	3
21	2,382.1	1,191.5	2,365.0	2,364.1	T	276.2		259.1	258.2	2
22	2,556.2	1,278.6	2,539.2	2,538.2	R	175.1		158.1		1

Sequence	Mascot Score	Modification	Observed (m/z)	Actual Mass	Charge	Error (ppm)	Position
(-)ELGmEEEDVIEVYQEQTGG-LKPYK(-)	96.35	Oxidation (M)	1400.6625	2799.3103	2	-2.20	K18

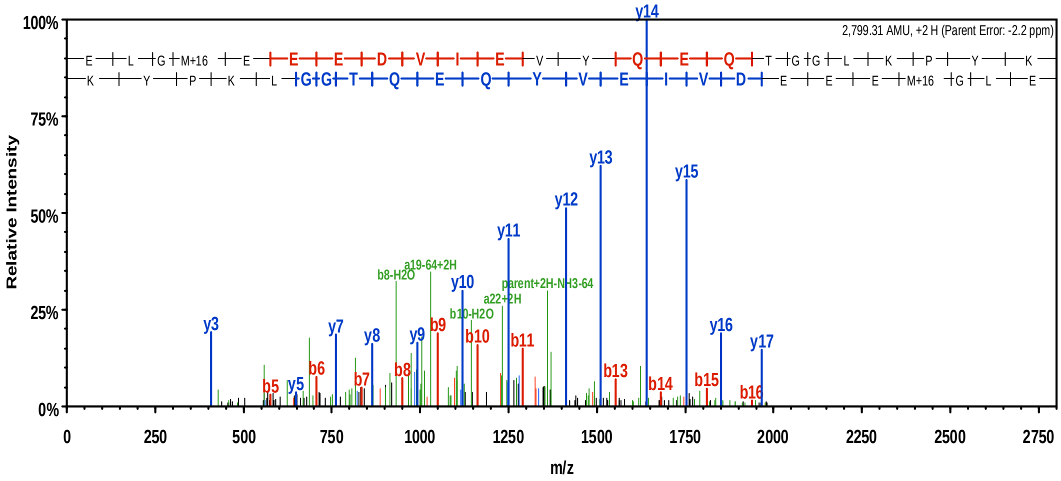

B	B Ions	B+2H	B-NH3	B-H2O	AA	Y Ions	Y+2H	Y-NH3	Y-H2O	Y
1	130.0			112.0	E	2,800.3	1,400.7	2,783.3	2,782.3	24
2	243.1			225.1	L	2,671.3	1,336.1	2,654.3	2,653.3	23
3	300.2			282.1	G	2,558.2	1,279.6	2,541.2	2,540.2	22
4	447.2			429.2	M+16	2,501.2	1,251.1	2,484.1	2,483.2	21
5	576.2			558.2	E	2,354.1	1,177.6	2,337.1	2,336.1	20
6	705.3	353.1		687.3	E	2,225.1	1,113.1	2,208.1	2,207.1	19
7	834.3	417.7		816.3	E	2,096.1	1,048.5	2,079.0	2,078.0	18
8	949.3	475.2		931.3	D	1,967.0	984.0	1,950.0	1,949.0	17
9	1,048.4	524.7		1,030.4	V	1,852.0	926.5	1,835.0	1,834.0	16
10	1,161.5	581.3		1,143.5	I	1,752.9	877.0	1,735.9	1,734.9	15
11	1,290.5	645.8		1,272.5	E	1,639.8	820.4	1,622.8	1,621.8	14
12	1,389.6	695.3		1,371.6	V	1,510.8	755.9	1,493.8	1,492.8	13
13	1,552.7	776.8		1,534.7	Y	1,411.7	706.4	1,394.7	1,393.7	12
14	1,680.7	840.9	1,663.7	1,662.7	Q	1,248.7	624.8	1,231.6	1,230.6	11
15	1,809.8	905.4	1,792.7	1,791.8	E	1,120.6	560.8	1,103.6	1,102.6	10
16	1,937.8	969.4	1,920.8	1,919.8	Q	991.6	496.3	974.5	973.5	9
17	2,038.9	1,019.9	2,021.9	2,020.9	T	863.5	432.3	846.5	845.5	8
18	2,095.9	1,048.5	2,078.9	2,077.9	G	762.5	381.7	745.4		7
19	2,152.9	1,077.0	2,135.9	2,134.9	G	705.4	353.2	688.4		6
20	2,266.0	1,133.5	2,249.0	2,248.0	L	648.4	324.7	631.4		5
21	2,394.1	1,197.6	2,377.1	2,376.1	K	535.3	268.2	518.3		4
22	2,491.2	1,246.1	2,474.1	2,473.1	P	407.2		390.2		3
23	2,654.2	1,327.6	2,637.2	2,636.2	Y	310.2		293.1		2
24	2,800.3	1,400.7	2,783.3	2,782.3	K	147.1		130.1		1

Sequence	Mascot Score	Modification	Observed (m/z)	Actual Mass	Charge	Error (ppm)	Position
(-)ELGmEEEDVIEVYQEQTGG-V**K**R(-)	113.11	Oxidation (M)	1277.601	2553.1873	2	-1.37	K167

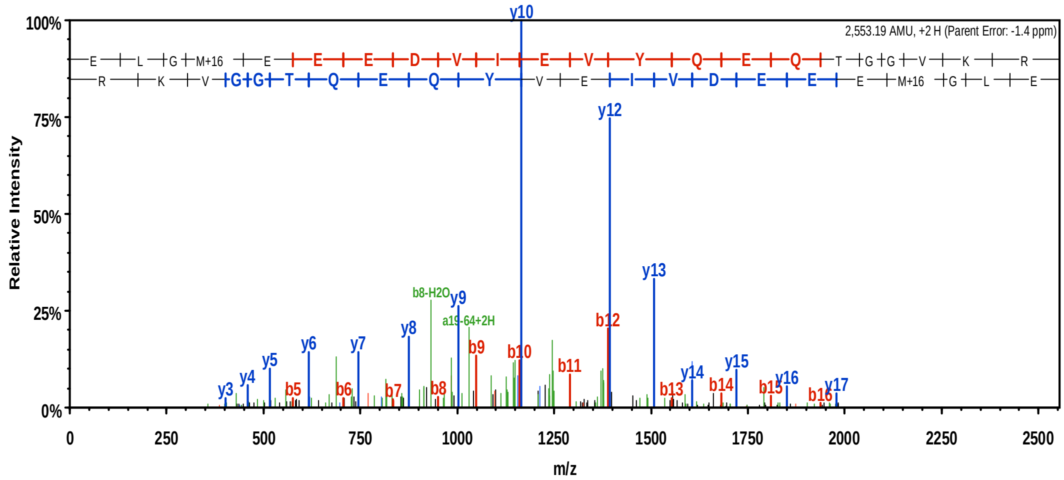

B	B Ions	B+2H	B-NH3	B-H2O	AA	Y Ions	Y+2H	Y-NH3	Y-H2O	Y
1	130.0			112.0	E	2,554.2	1,277.6	2,537.2	2,536.2	22
2	243.1			225.1	L	2,425.2	1,213.1	2,408.1	2,407.1	21
3	300.2			282.1	G	2,312.1	1,156.5	2,295.0	2,294.1	20
4	447.2			429.2	M+16	2,255.0	1,128.0	2,238.0	2,237.0	19
5	576.2			558.2	E	2,108.0	1,054.5	2,091.0	2,090.0	18
6	705.3	353.1		687.3	E	1,979.0	990.0	1,961.9	1,961.0	17
7	834.3	417.7		816.3	E	1,849.9	925.5	1,832.9	1,831.9	16
8	949.3	475.2		931.3	D	1,720.9	860.9	1,703.9	1,702.9	15
9	1,048.4	524.7		1,030.4	V	1,605.9	803.4	1,588.8	1,587.8	14
10	1,161.5	581.3		1,143.5	I	1,506.8	753.9	1,489.8	1,488.8	13
11	1,290.5	645.8		1,272.5	E	1,393.7	697.4	1,376.7	1,375.7	12
12	1,389.6	695.3		1,371.6	V	1,264.7	632.8	1,247.6	1,246.7	11
13	1,552.7	776.8		1,534.7	Y	1,165.6	583.3	1,148.6	1,147.6	10
14	1,680.7	840.9	1,663.7	1,662.7	Q	1,002.5	501.8	985.5	984.5	9
15	1,809.8	905.4	1,792.7	1,791.8	E	874.5	437.7	857.4	856.5	8
16	1,937.8	969.4	1,920.8	1,919.8	Q	745.4	373.2	728.4	727.4	7
17	2,038.9	1,019.9	2,021.9	2,020.9	T	617.4	309.2	600.3	599.4	6
18	2,095.9	1,048.5	2,078.9	2,077.9	G	516.3	258.7	499.3		5
19	2,152.9	1,077.0	2,135.9	2,134.9	G	459.3	230.2	442.3		4
20	2,252.0	1,126.5	2,235.0	2,234.0	V	402.3	201.6	385.3		3
21	2,380.1	1,190.5	2,363.1	2,362.1	K	303.2	152.1	286.2		2
22	2,554.2	1,277.6	2,537.2	2,536.2	R	175.1		158.1		1

Sequence	Mascot Score	Modification	Observed (m/z)	Actual Mass	Charge	Error (ppm)	Position
(-)ELGmEEEDVIEVYQEQTGG-NVKK(-)	114.38	Oxidation (M)	1320.6198	2639.2249	2	-1.00	K171

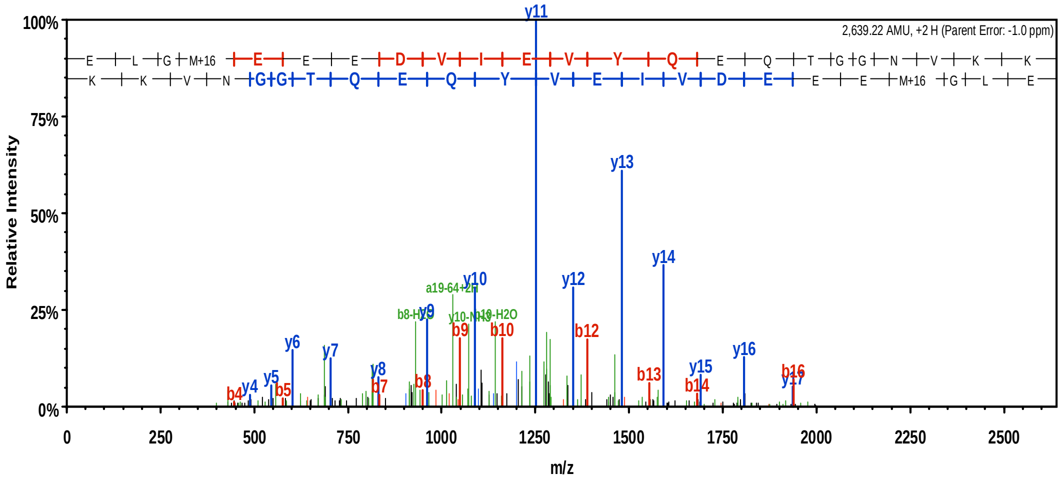

B	B Ions	B+2H	B-NH3	B-H2O	AA	Y Ions	Y+2H	Y-NH3	Y-H2O	Y
1	130.0			112.0	E	2,640.2	1,320.6	2,623.2	2,622.2	23
2	243.1			225.1	L	2,511.2	1,256.1	2,494.2	2,493.2	22
3	300.2			282.1	G	2,398.1	1,199.6	2,381.1	2,380.1	21
4	447.2			429.2	M+16	2,341.1	1,171.0	2,324.1	2,323.1	20
5	576.2			558.2	E	2,194.1	1,097.5	2,177.0	2,176.0	19
6	705.3	353.1		687.3	E	2,065.0	1,033.0	2,048.0	2,047.0	18
7	834.3	417.7		816.3	E	1,936.0	968.5	1,918.9	1,918.0	17
8	949.3	475.2		931.3	D	1,806.9	904.0	1,789.9	1,788.9	16
9	1,048.4	524.7		1,030.4	V	1,691.9	846.5	1,674.9	1,673.9	15
10	1,161.5	581.3		1,143.5	I	1,592.8	796.9	1,575.8	1,574.8	14
11	1,290.5	645.8		1,272.5	E	1,479.7	740.4	1,462.7	1,461.7	13
12	1,389.6	695.3		1,371.6	V	1,350.7	675.9	1,333.7	1,332.7	12
13	1,552.7	776.8		1,534.7	Y	1,251.6	626.3	1,234.6	1,233.6	11
14	1,680.7	840.9	1,663.7	1,662.7	Q	1,088.6	544.8	1,071.5	1,070.6	10
15	1,809.8	905.4	1,792.7	1,791.8	E	960.5	480.8	943.5	942.5	9
16	1,937.8	969.4	1,920.8	1,919.8	Q	831.5	416.2	814.4	813.5	8
17	2,038.9	1,019.9	2,021.9	2,020.9	T	703.4	352.2	686.4	685.4	7
18	2,095.9	1,048.5	2,078.9	2,077.9	G	602.4	301.7	585.3		6
19	2,152.9	1,077.0	2,135.9	2,134.9	G	545.3	273.2	528.3		5
20	2,267.0	1,134.0	2,249.9	2,249.0	N	488.3	244.7	471.3		4
21	2,366.0	1,183.5	2,349.0	2,348.0	V	374.3	187.6	357.2		3
22	2,494.1	1,247.6	2,477.1	2,476.1	K	275.2	138.1	258.2		2
23	2,640.2	1,320.6	2,623.2	2,622.2	K	147.1		130.1		1

Sequence	Mascot Score	Modification	Observed (m/z)	Actual Mass	Charge	Error (ppm)	Position
(-)ELGmEEEDVIEVYQEQTGG-KAPcPTGTVQILVHAGPPAIK(-)	95.68	Oxidation (M), Carbamidomethyl (C)	1436.379	4306.115	3	0.15	K172

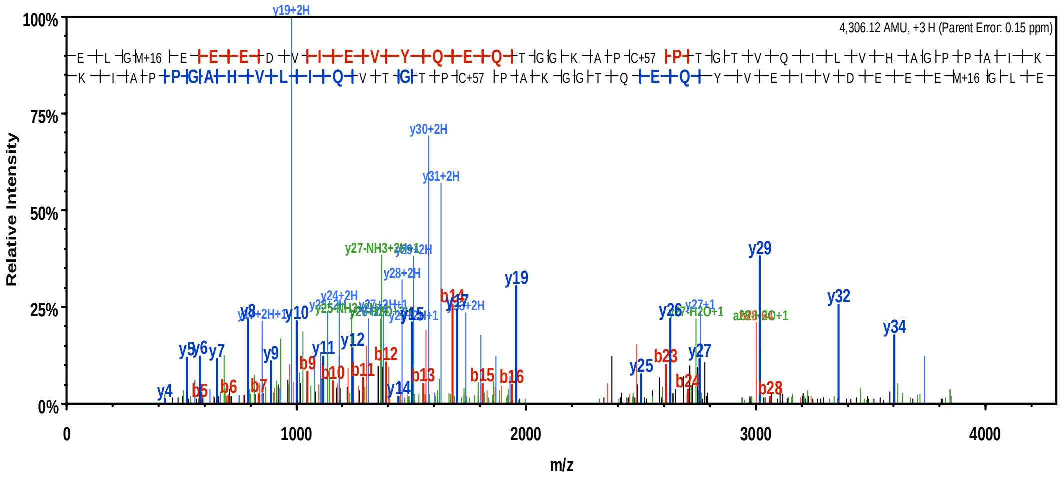

B	B Ions	B+2H	B-NH3	B-H2O	AA	Y Ions	Y+2H	Y-NH3	Y-H2O	Y
1	130.0			112.0	E	4,307.1	2,154.1	4,290.1	4,289.1	40
2	243.1			225.1	L	4,178.1	2,089.5	4,161.1	4,160.1	39
3	300.2			282.1	G	4,065.0	2,033.0	4,048.0	4,047.0	38
4	447.2			429.2	M+16	4,008.0	2,004.5	3,990.9	3,990.0	37
5	576.2			558.2	E	3,860.9	1,931.0	3,843.9	3,842.9	36
6	705.3	353.1		687.3	E	3,731.9	1,866.5	3,714.9	3,713.9	35
7	834.3	417.7		816.3	E	3,602.9	1,801.9	3,585.8	3,584.8	34
8	949.3	475.2		931.3	D	3,473.8	1,737.4	3,456.8	3,455.8	33
9	1,048.4	524.7		1,030.4	V	3,358.8	1,679.9	3,341.8	3,340.8	32
10	1,161.5	581.3		1,143.5	I	3,259.7	1,630.4	3,242.7	3,241.7	31
11	1,290.5	645.8		1,272.5	E	3,146.6	1,573.8	3,129.6	3,128.6	30
12	1,389.6	695.3		1,371.6	V	3,017.6	1,509.3	3,000.6	2,999.6	29
13	1,552.7	776.8		1,534.7	Y	2,918.5	1,459.8	2,901.5	2,900.5	28
14	1,680.7	840.9	1,663.7	1,662.7	Q	2,755.5	1,378.2	2,738.4	2,737.4	27
15	1,809.8	905.4	1,792.7	1,791.8	E	2,627.4	1,314.2	2,610.4	2,609.4	26
16	1,937.8	969.4	1,920.8	1,919.8	Q	2,498.4	1,249.7	2,481.3	2,480.3	25
17	2,038.9	1,019.9	2,021.9	2,020.9	T	2,370.3	1,185.7	2,353.3	2,352.3	24
18	2,095.9	1,048.5	2,078.9	2,077.9	G	2,269.2	1,135.1	2,252.2	2,251.2	23
19	2,152.9	1,077.0	2,135.9	2,134.9	G	2,212.2	1,106.6	2,195.2	2,194.2	22
20	2,281.0	1,141.0	2,264.0	2,263.0	K	2,155.2	1,078.1	2,138.2	2,137.2	21
21	2,352.1	1,176.5	2,335.0	2,334.0	A	2,027.1	1,014.1	2,010.1	2,009.1	20
22	2,449.1	1,225.1	2,432.1	2,431.1	P	1,956.1	978.5	1,939.0	1,938.1	19
23	2,609.1	1,305.1	2,592.1	2,591.1	C+57	1,859.0	930.0	1,842.0	1,841.0	18
24	2,706.2	1,353.6	2,689.2	2,688.2	P	1,699.0	850.0	1,682.0	1,681.0	17
25	2,807.2	1,404.1	2,790.2	2,789.2	T	1,601.9	801.5	1,584.9	1,583.9	16
26	2,864.3	1,432.6	2,847.2	2,846.2	G	1,500.9	750.9	1,483.9	1,482.9	15
27	2,965.3	1,483.2	2,948.3	2,947.3	T	1,443.9	722.4	1,426.8	1,425.9	14
28	3,064.4	1,532.7	3,047.3	3,046.4	V	1,342.8	671.9	1,325.8		13
29	3,192.4	1,596.7	3,175.4	3,174.4	Q	1,243.8	622.4	1,226.7		12
30	3,305.5	1,653.3	3,288.5	3,287.5	I	1,115.7	558.4	1,098.7		11
31	3,418.6	1,709.8	3,401.6	3,400.6	L	1,002.6	501.8	985.6		10
32	3,517.7	1,759.3	3,500.6	3,499.7	V	889.5	445.3	872.5		9
33	3,654.7	1,827.9	3,637.7	3,636.7	H	790.5	395.7	773.4		8
34	3,725.8	1,863.4	3,708.7	3,707.8	A	653.4	327.2	636.4		7
35	3,782.8	1,891.9	3,765.8	3,764.8	G	582.4	291.7	565.3		6
36	3,879.8	1,940.4	3,862.8	3,861.8	P	525.3		508.3		5
37	3,976.9	1,989.0	3,959.9	3,958.9	P	428.3		411.3		4
38	4,047.9	2,024.5	4,030.9	4,029.9	A	331.2		314.2		3
39	4,161.0	2,081.0	4,144.0	4,143.0	I	260.2		243.2		2
40	4,307.1	2,154.1	4,290.1	4,289.1	K	147.1		130.1		1

Sequence	Mascot Score	Modification	Observed (m/z)	Actual Mass	Charge	Error (ppm)	Position
(-)ELGmEEEDVIEVYQEQTGG-VSFHGVKNmR(-)	74.35	2 Oxidation (M)	1114.8519	3341.534	3	4.65	K215

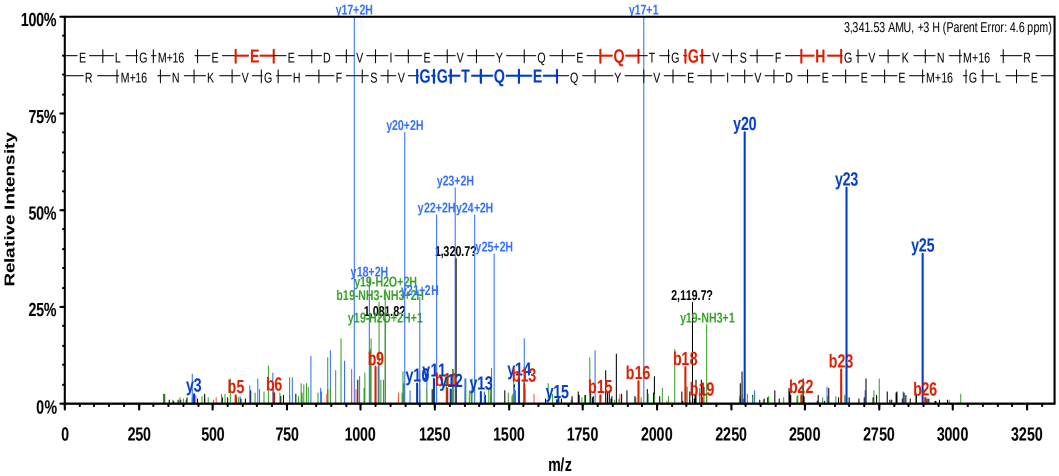

B	B Ions	B+2H	B-NH3	B-H2O	AA	Y Ions	Y+2H	Y-NH3	Y-H2O	Y
1	130.0			112.0	E	3,342.5	1,671.8	3,325.5	3,324.5	29
2	243.1			225.1	L	3,213.5	1,607.2	3,196.5	3,195.5	28
3	300.2			282.1	G	3,100.4	1,550.7	3,083.4	3,082.4	27
4	447.2			429.2	M+16	3,043.4	1,522.2	3,026.4	3,025.4	26
5	576.2			558.2	E	2,896.3	1,448.7	2,879.3	2,878.3	25
6	705.3	353.1		687.3	E	2,767.3	1,384.2	2,750.3	2,749.3	24
7	834.3	417.7		816.3	E	2,638.3	1,319.6	2,621.2	2,620.2	23
8	949.3	475.2		931.3	D	2,509.2	1,255.1	2,492.2	2,491.2	22
9	1,048.4	524.7		1,030.4	V	2,394.2	1,197.6	2,377.2	2,376.2	21
10	1,161.5	581.3		1,143.5	I	2,295.1	1,148.1	2,278.1	2,277.1	20
11	1,290.5	645.8		1,272.5	E	2,182.0	1,091.5	2,165.0	2,164.0	19
12	1,389.6	695.3		1,371.6	V	2,053.0	1,027.0	2,036.0	2,035.0	18
13	1,552.7	776.8		1,534.7	Y	1,953.9	977.5	1,936.9	1,935.9	17
14	1,680.7	840.9	1,663.7	1,662.7	Q	1,790.9	895.9	1,773.8	1,772.8	16
15	1,809.8	905.4	1,792.7	1,791.8	E	1,662.8	831.9	1,645.8	1,644.8	15
16	1,937.8	969.4	1,920.8	1,919.8	Q	1,533.8	767.4	1,516.7	1,515.7	14
17	2,038.9	1,019.9	2,021.9	2,020.9	T	1,405.7	703.4	1,388.7	1,387.7	13
18	2,095.9	1,048.5	2,078.9	2,077.9	G	1,304.7	652.8	1,287.6	1,286.6	12
19	2,152.9	1,077.0	2,135.9	2,134.9	G	1,247.6	624.3	1,230.6	1,229.6	11
20	2,252.0	1,126.5	2,235.0	2,234.0	V	1,190.6	595.8	1,173.6	1,172.6	10
21	2,339.0	1,170.0	2,322.0	2,321.0	S	1,091.5	546.3	1,074.5	1,073.5	9
22	2,486.1	1,243.5	2,469.1	2,468.1	F	1,004.5	502.8	987.5		8
23	2,623.2	1,312.1	2,606.1	2,605.1	H	857.4	429.2	840.4		7
24	2,680.2	1,340.6	2,663.1	2,662.2	G	720.4	360.7	703.4		6
25	2,779.2	1,390.1	2,762.2	2,761.2	V	663.4	332.2	646.3		5
26	2,907.3	1,454.2	2,890.3	2,889.3	K	564.3	282.6	547.3		4
27	3,021.4	1,511.2	3,004.4	3,003.4	N	436.2		419.2		3
28	3,168.4	1,584.7	3,151.4	3,150.4	M+16	322.2		305.1		2
29	3,342.5	1,671.8	3,325.5	3,324.5	R	175.1		158.1		1

Sequence	Mascot Score	Modification	Observed (m/z)	Actual Mass	Charge	Error (ppm)	Position
(-)ELGmEEEDVIEVYQEQTGG-GDPFDKNYVGNSGK(-)	79.21	Oxidation (M)	1217.2089	3648.6047	3	-0.24	K280

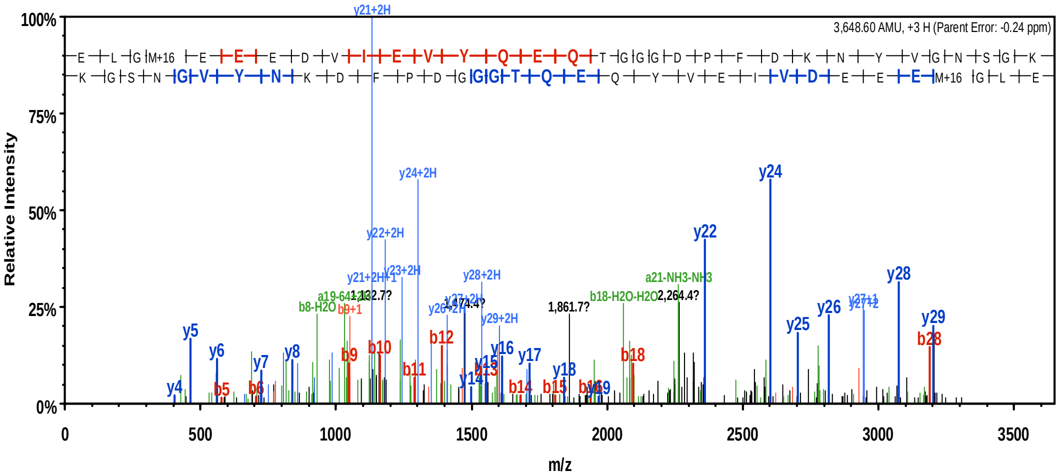

B	B Ions	B+2H	B-NH3	B-H2O	AA	Y Ions	Y+2H	Y-NH3	Y-H2O	Y
1	130.0			112.0	E	3,649.6	1,825.3	3,632.6	3,631.6	33
2	243.1			225.1	L	3,520.6	1,760.8	3,503.5	3,502.6	32
3	300.2			282.1	G	3,407.5	1,704.2	3,390.5	3,389.5	31
4	447.2			429.2	M+16	3,350.5	1,675.7	3,333.4	3,332.5	30
5	576.2			558.2	E	3,203.4	1,602.2	3,186.4	3,185.4	29
6	705.3	353.1		687.3	E	3,074.4	1,537.7	3,057.4	3,056.4	28
7	834.3	417.7		816.3	E	2,945.3	1,473.2	2,928.3	2,927.3	27
8	949.3	475.2		931.3	D	2,816.3	1,408.7	2,799.3	2,798.3	26
9	1,048.4	524.7		1,030.4	V	2,701.3	1,351.1	2,684.2	2,683.3	25
10	1,161.5	581.3		1,143.5	I	2,602.2	1,301.6	2,585.2	2,584.2	24
11	1,290.5	645.8		1,272.5	E	2,489.1	1,245.1	2,472.1	2,471.1	23
12	1,389.6	695.3		1,371.6	V	2,360.1	1,180.5	2,343.1	2,342.1	22
13	1,552.7	776.8		1,534.7	Y	2,261.0	1,131.0	2,244.0	2,243.0	21
14	1,680.7	840.9	1,663.7	1,662.7	Q	2,097.9	1,049.5	2,080.9	2,079.9	20
15	1,809.8	905.4	1,792.7	1,791.8	E	1,969.9	985.4	1,952.9	1,951.9	19
16	1,937.8	969.4	1,920.8	1,919.8	Q	1,840.8	920.9	1,823.8	1,822.8	18
17	2,038.9	1,019.9	2,021.9	2,020.9	T	1,712.8	856.9	1,695.8	1,694.8	17
18	2,095.9	1,048.5	2,078.9	2,077.9	G	1,611.7	806.4	1,594.7	1,593.7	16
19	2,152.9	1,077.0	2,135.9	2,134.9	G	1,554.7	777.9	1,537.7	1,536.7	15
20	2,209.9	1,105.5	2,192.9	2,191.9	G	1,497.7	749.4	1,480.7	1,479.7	14
21	2,325.0	1,163.0	2,307.9	2,307.0	D	1,440.7	720.8	1,423.6	1,422.7	13
22	2,422.0	1,211.5	2,405.0	2,404.0	P	1,325.6	663.3	1,308.6	1,307.6	12
23	2,569.1	1,285.0	2,552.1	2,551.1	F	1,228.6	614.8	1,211.6	1,210.6	11
24	2,684.1	1,342.6	2,667.1	2,666.1	D	1,081.5	541.3	1,064.5	1,063.5	10
25	2,812.2	1,406.6	2,795.2	2,794.2	K	966.5	483.8	949.5	948.5	9
26	2,926.3	1,463.6	2,909.2	2,908.2	N	838.4	419.7	821.4	820.4	8
27	3,089.3	1,545.2	3,072.3	3,071.3	Y	724.4	362.7	707.3	706.4	7
28	3,188.4	1,594.7	3,171.4	3,170.4	V	561.3	281.2	544.3	543.3	6
29	3,245.4	1,623.2	3,228.4	3,227.4	G	462.2		445.2	444.2	5
30	3,359.5	1,680.2	3,342.4	3,341.4	N	405.2		388.2	387.2	4
31	3,446.5	1,723.7	3,429.5	3,428.5	S	291.2		274.1	273.2	3
32	3,503.5	1,752.3	3,486.5	3,485.5	G	204.1		187.1		2
33	3,649.6	1,825.3	3,632.6	3,631.6	K	147.1		130.1		1

Sequence	Mascot Score	Modification	Observed (m/z)	Actual Mass	Charge	Error (ppm)	Position
(-)ELGmEEEDVIEVYQEQTGG-NYVGNSGKSR(-)	73.01	Oxidation (M)	1078.4899	3232.4478	3	0.30	K288

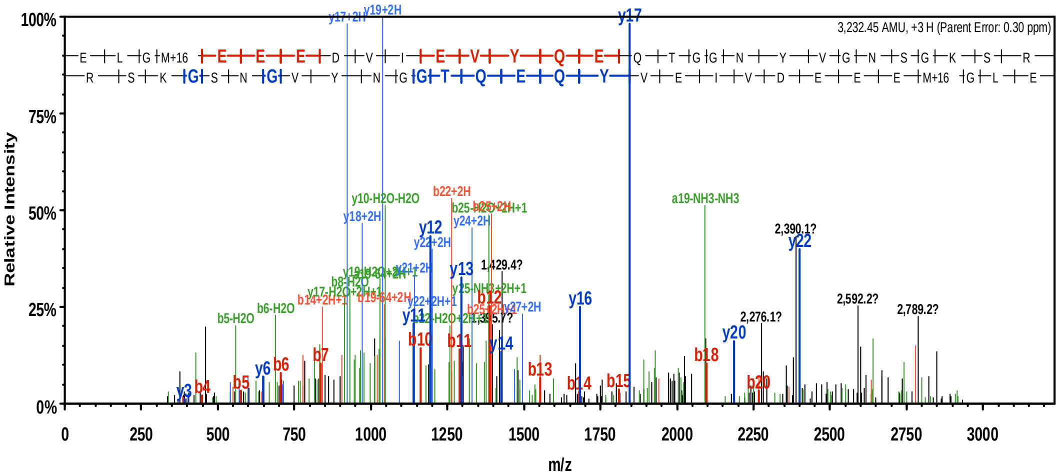

B	B Ions	B+2H	B-NH3	B-H2O	AA	Y Ions	Y+2H	Y-NH3	Y-H2O	Y
1	130.0			112.0	E	3,233.5	1,617.2	3,216.4	3,215.4	29
2	243.1			225.1	L	3,104.4	1,552.7	3,087.4	3,086.4	28
3	300.2			282.1	G	2,991.3	1,496.2	2,974.3	2,973.3	27
4	447.2			429.2	M+16	2,934.3	1,467.7	2,917.3	2,916.3	26
5	576.2			558.2	E	2,787.3	1,394.1	2,770.2	2,769.3	25
6	705.3	353.1		687.3	E	2,658.2	1,329.6	2,641.2	2,640.2	24
7	834.3	417.7		816.3	E	2,529.2	1,265.1	2,512.2	2,511.2	23
8	949.3	475.2		931.3	D	2,400.1	1,200.6	2,383.1	2,382.1	22
9	1,048.4	524.7		1,030.4	V	2,285.1	1,143.1	2,268.1	2,267.1	21
10	1,161.5	581.3		1,143.5	I	2,186.0	1,093.5	2,169.0	2,168.0	20
11	1,290.5	645.8		1,272.5	E	2,073.0	1,037.0	2,055.9	2,055.0	19
12	1,389.6	695.3		1,371.6	V	1,943.9	972.5	1,926.9	1,925.9	18
13	1,552.7	776.8		1,534.7	Y	1,844.9	922.9	1,827.8	1,826.8	17
14	1,680.7	840.9	1,663.7	1,662.7	Q	1,681.8	841.4	1,664.8	1,663.8	16
15	1,809.8	905.4	1,792.7	1,791.8	E	1,553.7	777.4	1,536.7	1,535.7	15
16	1,937.8	969.4	1,920.8	1,919.8	Q	1,424.7	712.8	1,407.7	1,406.7	14
17	2,038.9	1,019.9	2,021.9	2,020.9	T	1,296.6	648.8	1,279.6	1,278.6	13
18	2,095.9	1,048.5	2,078.9	2,077.9	G	1,195.6	598.3	1,178.6	1,177.6	12
19	2,152.9	1,077.0	2,135.9	2,134.9	G	1,138.6	569.8	1,121.5	1,120.5	11
20	2,267.0	1,134.0	2,249.9	2,249.0	N	1,081.5	541.3	1,064.5	1,063.5	10
21	2,430.0	1,215.5	2,413.0	2,412.0	Y	967.5	484.3	950.5	949.5	9
22	2,529.1	1,265.1	2,512.1	2,511.1	V	804.4	402.7	787.4	786.4	8
23	2,586.1	1,293.6	2,569.1	2,568.1	G	705.4	353.2	688.3	687.4	7
24	2,700.2	1,350.6	2,683.1	2,682.2	N	648.3	324.7	631.3	630.3	6
25	2,787.2	1,394.1	2,770.2	2,769.2	S	534.3	267.7	517.3	516.3	5
26	2,844.2	1,422.6	2,827.2	2,826.2	G	447.3	224.1	430.2	429.3	4
27	2,972.3	1,486.7	2,955.3	2,954.3	K	390.2	195.6	373.2	372.2	3
28	3,059.3	1,530.2	3,042.3	3,041.3	S	262.2		245.1	244.1	2
29	3,233.5	1,617.2	3,216.4	3,215.4	R	175.1		158.1		1

Sequence	Mascot Score	Modification	Observed (m/z)	Actual Mass	Charge	Error (ppm)	Position
(-)ELGmEEEDVIEVYQEQTGG-**K**HDR(-)	111.75	Oxidation (M)	1354.1093	2706.204	2	-1.58	K339

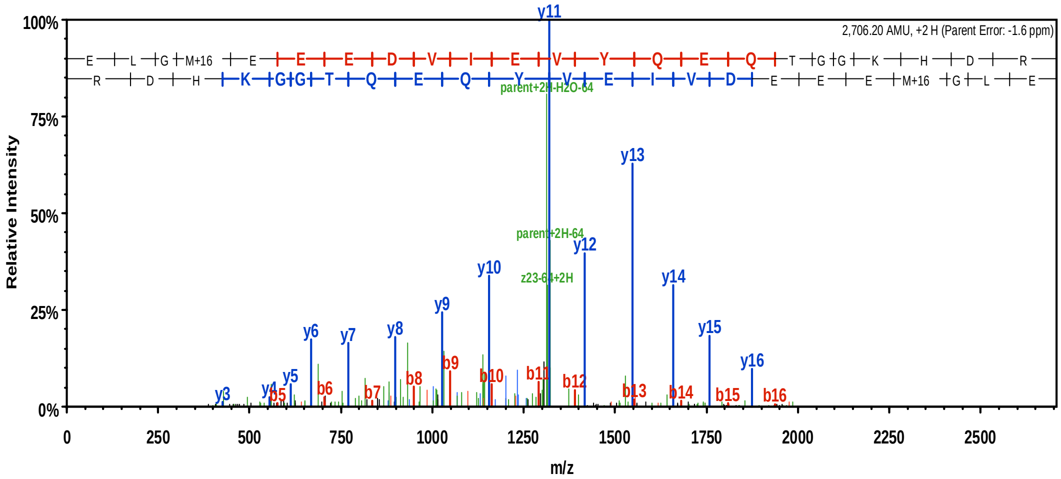

B	B Ions	B+2H	B-NH3	B-H2O	AA	Y Ions	Y+2H	Y-NH3	Y-H2O	Y
1	130.0			112.0	E	2,707.2	1,354.1	2,690.2	2,689.2	23
2	243.1			225.1	L	2,578.2	1,289.6	2,561.1	2,560.2	22
3	300.2			282.1	G	2,465.1	1,233.0	2,448.1	2,447.1	21
4	447.2			429.2	M+16	2,408.1	1,204.5	2,391.0	2,390.1	20
5	576.2			558.2	E	2,261.0	1,131.0	2,244.0	2,243.0	19
6	705.3	353.1		687.3	E	2,132.0	1,066.5	2,115.0	2,114.0	18
7	834.3	417.7		816.3	E	2,002.9	1,002.0	1,985.9	1,984.9	17
8	949.3	475.2		931.3	D	1,873.9	937.5	1,856.9	1,855.9	16
9	1,048.4	524.7		1,030.4	V	1,758.9	879.9	1,741.9	1,740.9	15
10	1,161.5	581.3		1,143.5	I	1,659.8	830.4	1,642.8	1,641.8	14
11	1,290.5	645.8		1,272.5	E	1,546.7	773.9	1,529.7	1,528.7	13
12	1,389.6	695.3		1,371.6	V	1,417.7	709.3	1,400.7	1,399.7	12
13	1,552.7	776.8		1,534.7	Y	1,318.6	659.8	1,301.6	1,300.6	11
14	1,680.7	840.9	1,663.7	1,662.7	Q	1,155.6	578.3	1,138.5	1,137.5	10
15	1,809.8	905.4	1,792.7	1,791.8	E	1,027.5	514.2	1,010.5	1,009.5	9
16	1,937.8	969.4	1,920.8	1,919.8	Q	898.4	449.7	881.4	880.4	8
17	2,038.9	1,019.9	2,021.9	2,020.9	T	770.4	385.7	753.4	752.4	7
18	2,095.9	1,048.5	2,078.9	2,077.9	G	669.3	335.2	652.3	651.3	6
19	2,152.9	1,077.0	2,135.9	2,134.9	G	612.3	306.7	595.3	594.3	5
20	2,281.0	1,141.0	2,264.0	2,263.0	K	555.3	278.2	538.3	537.3	4
21	2,418.1	1,209.5	2,401.1	2,400.1	H	427.2	214.1	410.2	409.2	3
22	2,533.1	1,267.1	2,516.1	2,515.1	D	290.1		273.1	272.1	2
23	2,707.2	1,354.1	2,690.2	2,689.2	R	175.1		158.1		1

Sequence	Mascot Score	Modification	Observed (m/z)	Actual Mass	Charge	Error (ppm)	Position
(-)ELGMEEEDVIEVYQEQTGG-KMSSGGGGGDHDHGLSSK(-)	61.49		1283.8971	3848.6697	3	-1.35	K352

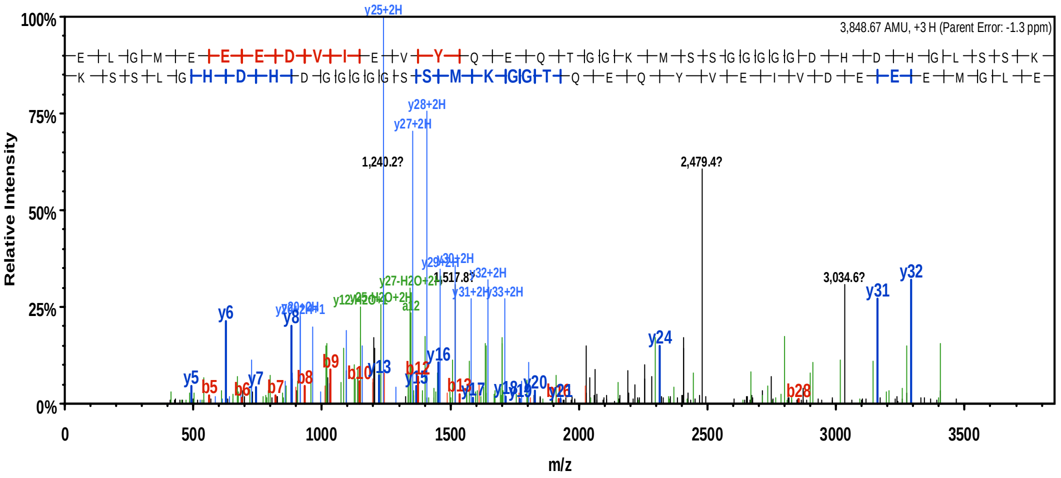

B	B Ions	B+2H	B-NH3	B-H2O	AA	Y Ions	Y+2H	Y-NH3	Y-H2O	Y
1	130.0			112.0	E	3,849.7	1,925.3	3,832.7	3,831.7	37
2	243.1			225.1	L	3,720.6	1,860.8	3,703.6	3,702.6	36
3	300.2			282.1	G	3,607.6	1,804.3	3,590.5	3,589.5	35
4	431.2			413.2	M	3,550.5	1,775.8	3,533.5	3,532.5	34
5	560.2			542.2	E	3,419.5	1,710.3	3,402.5	3,401.5	33
6	689.3	345.1		671.3	E	3,290.5	1,645.7	3,273.4	3,272.4	32
7	818.3	409.7		800.3	E	3,161.4	1,581.2	3,144.4	3,143.4	31
8	933.4	467.2		915.3	D	3,032.4	1,516.7	3,015.3	3,014.4	30
9	1,032.4	516.7		1,014.4	V	2,917.3	1,459.2	2,900.3	2,899.3	29
10	1,145.5	573.3		1,127.5	I	2,818.3	1,409.6	2,801.2	2,800.3	28
11	1,274.5	637.8		1,256.5	E	2,705.2	1,353.1	2,688.2	2,687.2	27
12	1,373.6	687.3		1,355.6	V	2,576.1	1,288.6	2,559.1	2,558.1	26
13	1,536.7	768.8		1,518.7	Y	2,477.1	1,239.0	2,460.0	2,459.1	25
14	1,664.7	832.9	1,647.7	1,646.7	Q	2,314.0	1,157.5	2,297.0	2,296.0	24
15	1,793.8	897.4	1,776.8	1,775.8	E	2,186.0	1,093.5	2,168.9	2,167.9	23
16	1,921.8	961.4	1,904.8	1,903.8	Q	2,056.9	1,029.0	2,039.9	2,038.9	22
17	2,022.9	1,011.9	2,005.9	2,004.9	T	1,928.9	964.9	1,911.8	1,910.8	21
18	2,079.9	1,040.5	2,062.9	2,061.9	G	1,827.8	914.4	1,810.8	1,809.8	20
19	2,136.9	1,069.0	2,119.9	2,118.9	G	1,770.8	885.9	1,753.8	1,752.8	19
20	2,265.0	1,133.0	2,248.0	2,247.0	K	1,713.8	857.4	1,696.7	1,695.8	18
21	2,396.1	1,198.5	2,379.0	2,378.1	M	1,585.7	793.3	1,568.6	1,567.7	17
22	2,483.1	1,242.1	2,466.1	2,465.1	S	1,454.6	727.8	1,437.6	1,436.6	16
23	2,570.1	1,285.6	2,553.1	2,552.1	S	1,367.6	684.3	1,350.6	1,349.6	15
24	2,627.1	1,314.1	2,610.1	2,609.1	G	1,280.6	640.8	1,263.5	1,262.6	14
25	2,684.2	1,342.6	2,667.1	2,666.2	G	1,223.5	612.3	1,206.5	1,205.5	13
26	2,741.2	1,371.1	2,724.2	2,723.2	G	1,166.5	583.8	1,149.5	1,148.5	12
27	2,798.2	1,399.6	2,781.2	2,780.2	G	1,109.5	555.3	1,092.5	1,091.5	11
28	2,855.2	1,428.1	2,838.2	2,837.2	G	1,052.5	526.7	1,035.4	1,034.5	10
29	2,970.3	1,485.6	2,953.2	2,952.3	D	995.5	498.2	978.4	977.4	9
30	3,107.3	1,554.2	3,090.3	3,089.3	H	880.4	440.7	863.4	862.4	8
31	3,222.3	1,611.7	3,205.3	3,204.3	D	743.4	372.2	726.3	725.4	7
32	3,359.4	1,680.2	3,342.4	3,341.4	H	628.3	314.7	611.3	610.3	6
33	3,416.4	1,708.7	3,399.4	3,398.4	G	491.3		474.3	473.3	5
34	3,529.5	1,765.3	3,512.5	3,511.5	L	434.3		417.2	416.3	4
35	3,616.5	1,808.8	3,599.5	3,598.5	S	321.2		304.2	303.2	3
36	3,703.6	1,852.3	3,686.5	3,685.6	S	234.1		217.1	216.1	2
37	3,849.7	1,925.3	3,832.7	3,831.7	K	147.1		130.1		1

Sequence	Mascot Score	Modification	Observed (m/z)	Actual Mass	Charge	Error (ppm)	Position
(-)ELGMEEEDVIEVYQEQTGG-E**K**YEQHK(-)	52.16		1033.1385	3096.3938	3	2.1	K371

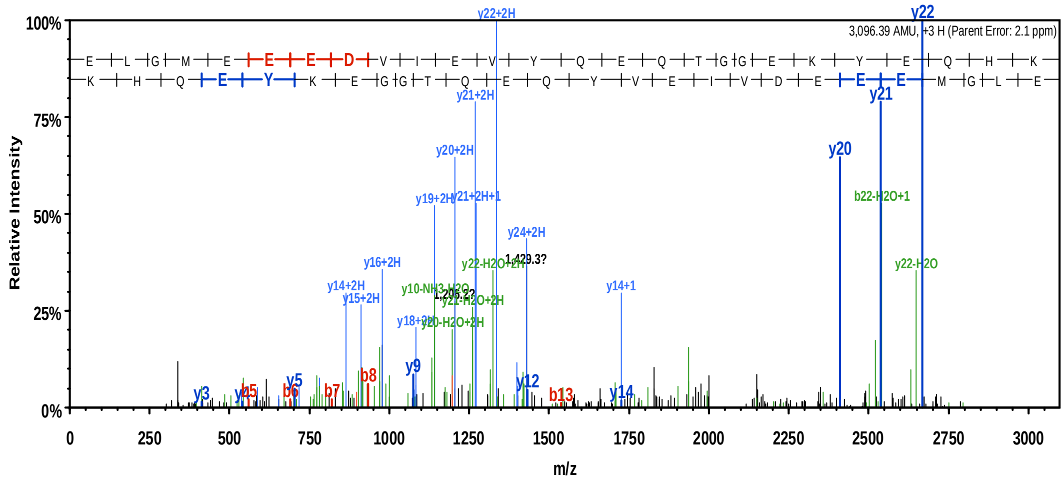

B	B Ions	B+2H	B-NH3	B-H2O	AA	Y Ions	Y+2H	Y-NH3	Y-H2O	Y
1	130.0			112.0	E	3,097.4	1,549.2	3,080.4	3,079.4	26
2	243.1			225.1	L	2,968.4	1,484.7	2,951.3	2,950.3	25
3	300.2			282.1	G	2,855.3	1,428.1	2,838.2	2,837.3	24
4	431.2			413.2	M	2,798.2	1,399.6	2,781.2	2,780.2	23
5	560.2			542.2	E	2,667.2	1,334.1	2,650.2	2,649.2	22
6	689.3	345.1		671.3	E	2,538.2	1,269.6	2,521.1	2,520.2	21
7	818.3	409.7		800.3	E	2,409.1	1,205.1	2,392.1	2,391.1	20
8	933.4	467.2		915.3	D	2,280.1	1,140.5	2,263.1	2,262.1	19
9	1,032.4	516.7		1,014.4	V	2,165.1	1,083.0	2,148.0	2,147.0	18
10	1,145.5	573.3		1,127.5	I	2,066.0	1,033.5	2,049.0	2,048.0	17
11	1,274.5	637.8		1,256.5	E	1,952.9	977.0	1,935.9	1,934.9	16
12	1,373.6	687.3		1,355.6	V	1,823.9	912.4	1,806.8	1,805.8	15
13	1,536.7	768.8		1,518.7	Y	1,724.8	862.9	1,707.8	1,706.8	14
14	1,664.7	832.9	1,647.7	1,646.7	Q	1,561.7	781.4	1,544.7	1,543.7	13
15	1,793.8	897.4	1,776.8	1,775.8	E	1,433.7	717.3	1,416.6	1,415.7	12
16	1,921.8	961.4	1,904.8	1,903.8	Q	1,304.6	652.8	1,287.6	1,286.6	11
17	2,022.9	1,011.9	2,005.9	2,004.9	T	1,176.6	588.8	1,159.5	1,158.6	10
18	2,079.9	1,040.5	2,062.9	2,061.9	G	1,075.5	538.3	1,058.5	1,057.5	9
19	2,136.9	1,069.0	2,119.9	2,118.9	G	1,018.5	509.8	1,001.5	1,000.5	8
20	2,266.0	1,133.5	2,248.9	2,248.0	E	961.5	481.2	944.4	943.5	7
21	2,394.1	1,197.5	2,377.0	2,376.1	K	832.4	416.7	815.4	814.4	6
22	2,557.1	1,279.1	2,540.1	2,539.1	Y	704.3	352.7	687.3	686.3	5
23	2,686.2	1,343.6	2,669.1	2,668.2	E	541.3	271.1	524.2	523.3	4
24	2,814.2	1,407.6	2,797.2	2,796.2	Q	412.2	206.6	395.2		3
25	2,951.3	1,476.1	2,934.3	2,933.3	H	284.2	142.6	267.1		2
26	3,097.4	1,549.2	3,080.4	3,079.4	K	147.1		130.1		1

Sequence	Mascot Score	Modification	Observed (m/z)	Actual Mass	Charge	Error (ppm)	Position
(-)ELGmEEEDVIEVYQEQTGG-YEQHKITSYLTSK(-)	56.68	Oxidation (M)	1250.5829	3748.7268	3	-1.00	K376

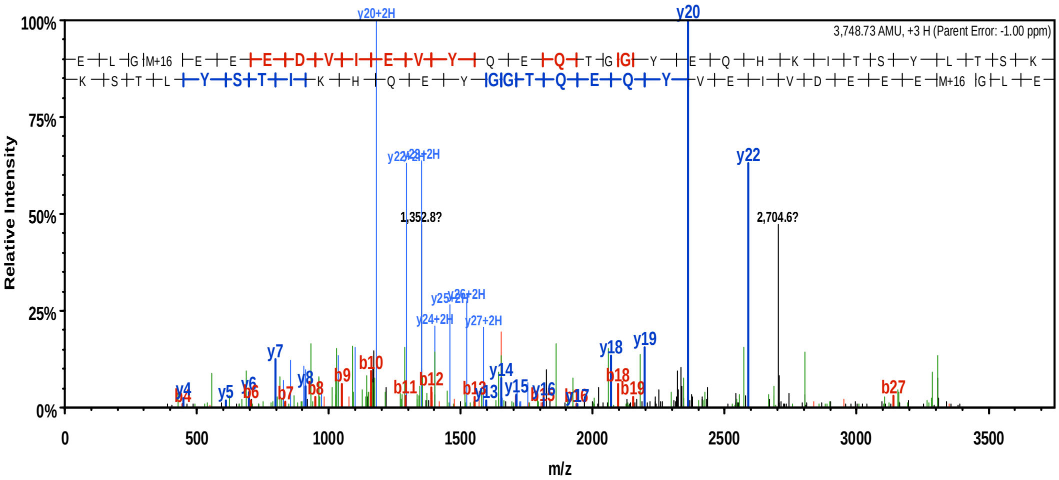

B	B Ions	B+2H	B-NH3	B-H2O	AA	Y Ions	Y+2H	Y-NH3	Y-H2O	Y
1	130.0			112.0	E	3,749.7	1,875.4	3,732.7	3,731.7	32
2	243.1			225.1	L	3,620.7	1,810.9	3,603.7	3,602.7	31
3	300.2			282.1	G	3,507.6	1,754.3	3,490.6	3,489.6	30
4	447.2			429.2	M+16	3,450.6	1,725.8	3,433.6	3,432.6	29
5	576.2			558.2	E	3,303.6	1,652.3	3,286.5	3,285.5	28
6	705.3	353.1		687.3	E	3,174.5	1,587.8	3,157.5	3,156.5	27
7	834.3	417.7		816.3	E	3,045.5	1,523.2	3,028.4	3,027.5	26
8	949.3	475.2		931.3	D	2,916.4	1,458.7	2,899.4	2,898.4	25
9	1,048.4	524.7		1,030.4	V	2,801.4	1,401.2	2,784.4	2,783.4	24
10	1,161.5	581.3		1,143.5	I	2,702.3	1,351.7	2,685.3	2,684.3	23
11	1,290.5	645.8		1,272.5	E	2,589.2	1,295.1	2,572.2	2,571.2	22
12	1,389.6	695.3		1,371.6	V	2,460.2	1,230.6	2,443.2	2,442.2	21
13	1,552.7	776.8		1,534.7	Y	2,361.1	1,181.1	2,344.1	2,343.1	20
14	1,680.7	840.9	1,663.7	1,662.7	Q	2,198.1	1,099.5	2,181.0	2,180.1	19
15	1,809.8	905.4	1,792.7	1,791.8	E	2,070.0	1,035.5	2,053.0	2,052.0	18
16	1,937.8	969.4	1,920.8	1,919.8	Q	1,941.0	971.0	1,923.9	1,923.0	17
17	2,038.9	1,019.9	2,021.9	2,020.9	T	1,812.9	907.0	1,795.9	1,794.9	16
18	2,095.9	1,048.5	2,078.9	2,077.9	G	1,711.9	856.4	1,694.8	1,693.9	15
19	2,152.9	1,077.0	2,135.9	2,134.9	G	1,654.8	827.9	1,637.8	1,636.8	14
20	2,316.0	1,158.5	2,299.0	2,298.0	Y	1,597.8	799.4	1,580.8	1,579.8	13
21	2,445.0	1,223.0	2,428.0	2,427.0	E	1,434.8	717.9	1,417.7	1,416.7	12
22	2,573.1	1,287.0	2,556.1	2,555.1	Q	1,305.7	653.4	1,288.7	1,287.7	11
23	2,710.1	1,355.6	2,693.1	2,692.1	H	1,177.7	589.3	1,160.6	1,159.6	10
24	2,838.2	1,419.6	2,821.2	2,820.2	K	1,040.6	520.8	1,023.6	1,022.6	9
25	2,951.3	1,476.2	2,934.3	2,933.3	I	912.5	456.8	895.5	894.5	8
26	3,052.4	1,526.7	3,035.3	3,034.4	T	799.4	400.2	782.4	781.4	7
27	3,139.4	1,570.2	3,122.4	3,121.4	S	698.4	349.7	681.3	680.4	6
28	3,302.5	1,651.7	3,285.4	3,284.5	Y	611.3		594.3	593.3	5
29	3,415.6	1,708.3	3,398.5	3,397.5	L	448.3		431.3	430.3	4
30	3,516.6	1,758.8	3,499.6	3,498.6	T	335.2		318.2	317.2	3
31	3,603.6	1,802.3	3,586.6	3,585.6	S	234.1		217.1	216.1	2
32	3,749.7	1,875.4	3,732.7	3,731.7	K	147.1		130.1		1

Sequence	Mascot Score	Modification	Observed (m/z)	Actual Mass	Charge	Error (ppm)	Position
(-)ELGmEEEDVIEVYQEQTGG-ITSYLTSKGGSGGGGGGGGGGLDR(-)	64.86	Oxidation (M)	1407.3035	4218.8887	3	-6.11	K384

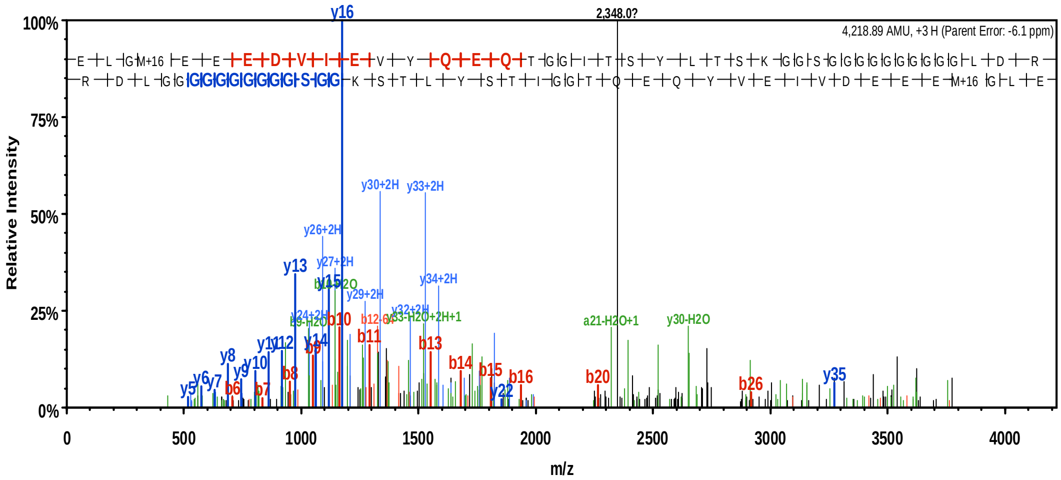

B	B Ions	B+2H	B-NH3	B-H2O	AA	Y Ions	Y+2H	Y-NH3	Y-H2O	Y
1	130.0			112.0	E	4,219.9	2,110.5	4,202.9	4,201.9	43
2	243.1			225.1	L	4,090.9	2,045.9	4,073.9	4,072.9	42
3	300.2			282.1	G	3,977.8	1,989.4	3,960.8	3,959.8	41
4	447.2			429.2	M+16	3,920.8	1,960.9	3,903.7	3,902.8	40
5	576.2			558.2	E	3,773.7	1,887.4	3,756.7	3,755.7	39
6	705.3	353.1		687.3	E	3,644.7	1,822.9	3,627.7	3,626.7	38
7	834.3	417.7		816.3	E	3,515.7	1,758.3	3,498.6	3,497.6	37
8	949.3	475.2		931.3	D	3,386.6	1,693.8	3,369.6	3,368.6	36
9	1,048.4	524.7		1,030.4	V	3,271.6	1,636.3	3,254.6	3,253.6	35
10	1,161.5	581.3		1,143.5	I	3,172.5	1,586.8	3,155.5	3,154.5	34
11	1,290.5	645.8		1,272.5	E	3,059.4	1,530.2	3,042.4	3,041.4	33
12	1,389.6	695.3		1,371.6	V	2,930.4	1,465.7	2,913.4	2,912.4	32
13	1,552.7	776.8		1,534.7	Y	2,831.3	1,416.2	2,814.3	2,813.3	31
14	1,680.7	840.9	1,663.7	1,662.7	Q	2,668.3	1,334.6	2,651.2	2,650.2	30
15	1,809.8	905.4	1,792.7	1,791.8	E	2,540.2	1,270.6	2,523.2	2,522.2	29
16	1,937.8	969.4	1,920.8	1,919.8	Q	2,411.2	1,206.1	2,394.1	2,393.1	28
17	2,038.9	1,019.9	2,021.9	2,020.9	T	2,283.1	1,142.1	2,266.1	2,265.1	27
18	2,095.9	1,048.5	2,078.9	2,077.9	G	2,182.0	1,091.5	2,165.0	2,164.0	26
19	2,152.9	1,077.0	2,135.9	2,134.9	G	2,125.0	1,063.0	2,108.0	2,107.0	25
20	2,266.0	1,133.5	2,249.0	2,248.0	I	2,068.0	1,034.5	2,051.0	2,050.0	24
21	2,367.1	1,184.0	2,350.0	2,349.0	T	1,954.9	978.0	1,937.9	1,936.9	23
22	2,454.1	1,227.5	2,437.1	2,436.1	S	1,853.9	927.4	1,836.8	1,835.9	22
23	2,617.1	1,309.1	2,600.1	2,599.1	Y	1,766.8	883.9	1,749.8	1,748.8	21
24	2,730.2	1,365.6	2,713.2	2,712.2	L	1,603.8	802.4	1,586.8	1,585.8	20
25	2,831.3	1,416.1	2,814.3	2,813.3	T	1,490.7	745.9	1,473.7	1,472.7	19
26	2,918.3	1,459.7	2,901.3	2,900.3	S	1,389.6	695.3	1,372.6	1,371.6	18
27	3,046.4	1,523.7	3,029.4	3,028.4	K	1,302.6	651.8	1,285.6	1,284.6	17
28	3,103.4	1,552.2	3,086.4	3,085.4	G	1,174.5	587.8	1,157.5	1,156.5	16
29	3,160.5	1,580.7	3,143.4	3,142.4	G	1,117.5	559.3	1,100.5	1,099.5	15
30	3,247.5	1,624.2	3,230.5	3,229.5	S	1,060.5	530.7	1,043.5	1,042.5	14
31	3,304.5	1,652.8	3,287.5	3,286.5	G	973.4	487.2	956.4	955.4	13
32	3,361.5	1,681.3	3,344.5	3,343.5	G	916.4	458.7	899.4	898.4	12
33	3,418.5	1,709.8	3,401.5	3,400.5	G	859.4	430.2	842.4	841.4	11
34	3,475.6	1,738.3	3,458.5	3,457.6	G	802.4	401.7	785.4	784.4	10
35	3,532.6	1,766.8	3,515.6	3,514.6	G	745.4	373.2	728.3	727.3	9
36	3,589.6	1,795.3	3,572.6	3,571.6	G	688.3	344.7	671.3	670.3	8
37	3,646.6	1,823.8	3,629.6	3,628.6	G	631.3	316.2	614.3	613.3	7
38	3,703.7	1,852.3	3,686.6	3,685.6	G	574.3	287.7	557.3	556.3	6
39	3,760.7	1,880.8	3,743.6	3,742.7	G	517.3		500.2	499.3	5
40	3,817.7	1,909.4	3,800.7	3,799.7	G	460.3		443.2	442.2	4
41	3,930.8	1,965.9	3,913.8	3,912.8	L	403.2		386.2	385.2	3
42	4,045.8	2,023.4	4,028.8	4,027.8	D	290.1		273.1	272.1	2
43	4,219.9	2,110.5	4,202.9	4,201.9	R	175.1		158.1		1

Sequence	Mascot Score	Observed (m/z)	Actual Mass	Charge	Error (ppm)	Position
(-)IADNHTPKELGMEEEDVIEVYQEQTGG-NSGNYFNDAKEESDSEDSVTFEFVPNTKK(-)	40.74	1578.4583	6309.804	4	-3.31	K410

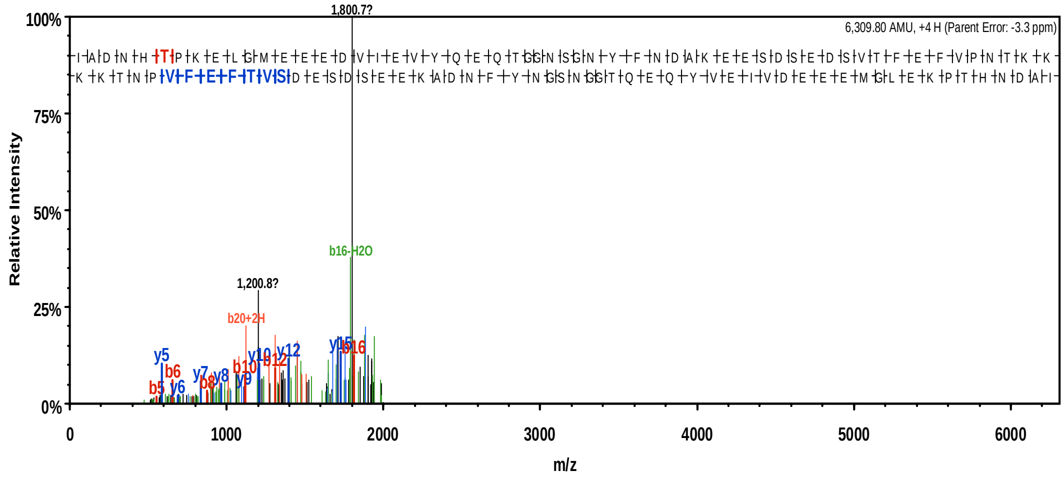

B	B Ions	B+2H	B-NH3	B-H2O	AA	Y Ions	Y+2H	Y-NH3	Y-H2O	Y
1	114.1				I	6,310.8	3,155.9	6,293.8	6,292.8	56
2	185.1				A	6,197.7	3,099.4	6,180.7	6,179.7	55
3	300.2			282.1	D	6,126.7	3,063.9	6,109.7	6,108.7	54
4	414.2		397.2	396.2	N	6,011.7	3,006.3	5,994.7	5,993.7	53
5	551.3	276.1	534.2	533.2	H	5,897.6	2,949.3	5,880.6	5,879.6	52
6	652.3	326.7	635.3	634.3	T	5,760.6	2,880.8	5,743.6	5,742.6	51
7	749.4	375.2	732.3	731.3	P	5,659.5	2,830.3	5,642.5	5,641.5	50
8	877.5	439.2	860.4	859.4	K	5,562.5	2,781.7	5,545.5	5,544.5	49
9	1,006.5	503.8	989.5	988.5	E	5,434.4	2,717.7	5,417.4	5,416.4	48
10	1,119.6	560.3	1,102.6	1,101.6	L	5,305.3	2,653.2	5,288.3	5,287.3	47
11	1,176.6	588.8	1,159.6	1,158.6	G	5,192.3	2,596.6	5,175.2	5,174.2	46
12	1,307.6	654.3	1,290.6	1,289.6	M	5,135.2	2,568.1	5,118.2	5,117.2	45
13	1,436.7	718.8	1,419.7	1,418.7	E	5,004.2	2,502.6	4,987.2	4,986.2	44
14	1,565.7	783.4	1,548.7	1,547.7	E	4,875.2	2,438.1	4,858.1	4,857.1	43
15	1,694.8	847.9	1,677.7	1,676.8	E	4,746.1	2,373.6	4,729.1	4,728.1	42
16	1,809.8	905.4	1,792.8	1,791.8	D	4,617.1	2,309.0	4,600.0	4,599.1	41
17	1,908.9	954.9	1,891.8	1,890.9	V	4,502.0	2,251.5	4,485.0	4,484.0	40
18	2,021.9	1,011.5	2,004.9	2,003.9	I	4,403.0	2,202.0	4,385.9	4,385.0	39
19	2,151.0	1,076.0	2,134.0	2,133.0	E	4,289.9	2,145.4	4,272.9	4,271.9	38
20	2,250.1	1,125.5	2,233.0	2,232.0	V	4,160.8	2,080.9	4,143.8	4,142.8	37
21	2,413.1	1,207.1	2,396.1	2,395.1	Y	4,061.8	2,031.4	4,044.8	4,043.8	36
22	2,541.2	1,271.1	2,524.2	2,523.2	Q	3,898.7	1,949.9	3,881.7	3,880.7	35
23	2,670.2	1,335.6	2,653.2	2,652.2	E	3,770.7	1,885.8	3,753.6	3,752.6	34
24	2,798.3	1,399.6	2,781.3	2,780.3	Q	3,641.6	1,821.3	3,624.6	3,623.6	33
25	2,899.3	1,450.2	2,882.3	2,881.3	T	3,513.6	1,757.3	3,496.5	3,495.5	32
26	2,956.4	1,478.7	2,939.3	2,938.3	G	3,412.5	1,706.8	3,395.5	3,394.5	31
27	3,013.4	1,507.2	2,996.3	2,995.4	G	3,355.5	1,678.2	3,338.5	3,337.5	30
28	3,127.4	1,564.2	3,110.4	3,109.4	N	3,298.5	1,649.7	3,281.4	3,280.5	29
29	3,214.4	1,607.7	3,197.4	3,196.4	S	3,184.4	1,592.7	3,167.4	3,166.4	28
30	3,271.5	1,636.2	3,254.4	3,253.5	G	3,097.4	1,549.2	3,080.4	3,079.4	27
31	3,385.5	1,693.3	3,368.5	3,367.5	N	3,040.4	1,520.7	3,023.3	3,022.4	26
32	3,548.6	1,774.8	3,531.5	3,530.6	Y	2,926.3	1,463.7	2,909.3	2,908.3	25
33	3,695.6	1,848.3	3,678.6	3,677.6	F	2,763.3	1,382.1	2,746.2	2,745.3	24
34	3,809.7	1,905.3	3,792.7	3,791.7	N	2,616.2	1,308.6	2,599.2	2,598.2	23
35	3,924.7	1,962.9	3,907.7	3,906.7	D	2,502.2	1,251.6	2,485.1	2,484.1	22
36	3,995.8	1,998.4	3,978.7	3,977.7	A	2,387.1	1,194.1	2,370.1	2,369.1	21
37	4,123.8	2,062.4	4,106.8	4,105.8	K	2,316.1	1,158.5	2,299.1	2,298.1	20
38	4,252.9	2,126.9	4,235.9	4,234.9	E	2,188.0	1,094.5	2,171.0	2,170.0	19
39	4,381.9	2,191.5	4,364.9	4,363.9	E	2,059.0	1,030.0	2,041.9	2,040.9	18
40	4,469.0	2,235.0	4,451.9	4,451.0	S	1,929.9	965.5	1,912.9	1,911.9	17
41	4,584.0	2,292.5	4,567.0	4,566.0	D	1,842.9	921.9	1,825.8	1,824.9	16
42	4,671.0	2,336.0	4,654.0	4,653.0	S	1,727.8	864.4	1,710.8	1,709.8	15
43	4,800.1	2,400.5	4,783.0	4,782.1	E	1,640.8	820.9	1,623.8	1,622.8	14
44	4,915.1	2,458.0	4,898.1	4,897.1	D	1,511.8	756.4	1,494.7	1,493.8	13
45	5,002.1	2,501.6	4,985.1	4,984.1	S	1,396.7	698.9	1,379.7	1,378.7	12
46	5,101.2	2,551.1	5,084.2	5,083.2	V	1,309.7	655.4	1,292.7	1,291.7	11
47	5,202.2	2,601.6	5,185.2	5,184.2	T	1,210.6	605.8	1,193.6	1,192.6	10
48	5,349.3	2,675.2	5,332.3	5,331.3	F	1,109.6	555.3	1,092.6	1,091.6	9
49	5,478.4	2,739.7	5,461.3	5,460.3	E	962.5	481.8	945.5	944.5	8
50	5,625.4	2,813.2	5,608.4	5,607.4	F	833.5	417.2	816.5	815.5	7
51	5,724.5	2,862.7	5,707.5	5,706.5	V	686.4	343.7	669.4	668.4	6
52	5,821.5	2,911.3	5,804.5	5,803.5	P	587.4	294.2	570.3	569.3	5
53	5,935.6	2,968.3	5,918.6	5,917.6	N	490.3	245.7	473.3	472.3	4
54	6,036.6	3,018.8	6,019.6	6,018.6	T	376.3	188.6	359.2	358.2	3
55	6,164.7	3,082.9	6,147.7	6,146.7	K	275.2	138.1	258.2		2
56	6,310.8	3,155.9	6,293.8	6,292.8	K	147.1		130.1		1

Sequence	Mascot Score	Modification	Observed (m/z)	Actual Mass	Charge	Error (ppm)	Position
(-)ELGmEEEDVIEVYQEQTGG-KQK(-)	106.9	Oxidation (M)	1278.0902	2554.1658	2	-3.56	K429

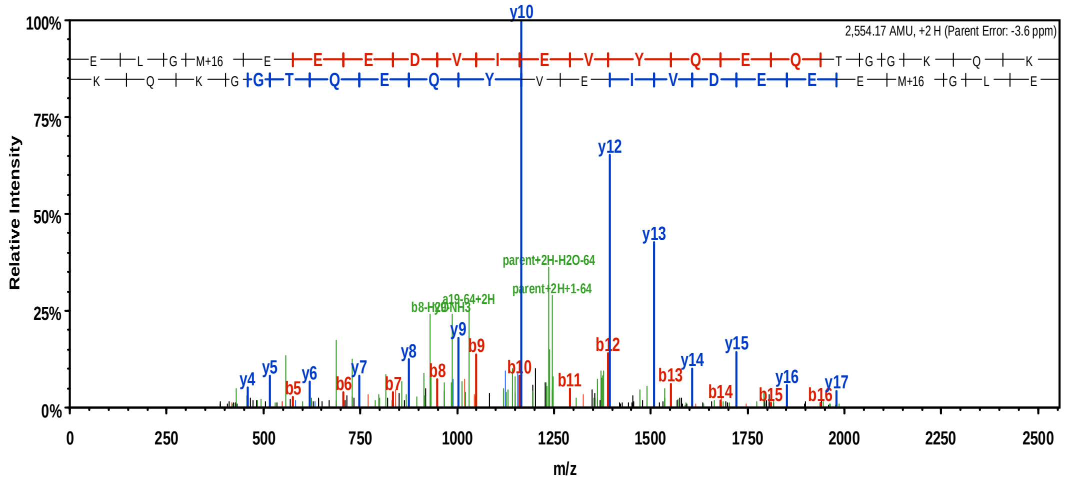

B	B Ions	B+2H	B-NH3	B-H2O	AA	Y Ions	Y+2H	Y-NH3	Y-H2O	Y
1	130.0			112.0	E	2,555.2	1,278.1	2,538.2	2,537.2	22
2	243.1			225.1	L	2,426.1	1,213.6	2,409.1	2,408.1	21
3	300.2			282.1	G	2,313.1	1,157.0	2,296.0	2,295.0	20
4	447.2			429.2	M+16	2,256.0	1,128.5	2,239.0	2,238.0	19
5	576.2			558.2	E	2,109.0	1,055.0	2,092.0	2,091.0	18
6	705.3	353.1		687.3	E	1,980.0	990.5	1,962.9	1,961.9	17
7	834.3	417.7		816.3	E	1,850.9	926.0	1,833.9	1,832.9	16
8	949.3	475.2		931.3	D	1,721.9	861.4	1,704.8	1,703.9	15
9	1,048.4	524.7		1,030.4	V	1,606.8	803.9	1,589.8	1,588.8	14
10	1,161.5	581.3		1,143.5	I	1,507.8	754.4	1,490.7	1,489.8	13
11	1,290.5	645.8		1,272.5	E	1,394.7	697.8	1,377.7	1,376.7	12
12	1,389.6	695.3		1,371.6	V	1,265.6	633.3	1,248.6	1,247.6	11
13	1,552.7	776.8		1,534.7	Y	1,166.6	583.8	1,149.6	1,148.6	10
14	1,680.7	840.9	1,663.7	1,662.7	Q	1,003.5	502.3	986.5	985.5	9
15	1,809.8	905.4	1,792.7	1,791.8	E	875.5	438.2	858.4	857.4	8
16	1,937.8	969.4	1,920.8	1,919.8	Q	746.4	373.7	729.4	728.4	7
17	2,038.9	1,019.9	2,021.9	2,020.9	T	618.4	309.7	601.3	600.3	6
18	2,095.9	1,048.5	2,078.9	2,077.9	G	517.3	259.2	500.3		5
19	2,152.9	1,077.0	2,135.9	2,134.9	G	460.3	230.6	443.3		4
20	2,281.0	1,141.0	2,264.0	2,263.0	K	403.3	202.1	386.2		3
21	2,409.1	1,205.0	2,392.0	2,391.1	Q	275.2		258.1		2
22	2,555.2	1,278.1	2,538.2	2,537.2	K	147.1		130.1		1

Sequence	Mascot Score	Modification	Observed (m/z)	Actual Mass	Charge	Error (ppm)	Position
(-)ELGmEEEDVIEVYQEQTGG-KQKcGTEEF(-)	74.38	Oxidation (M), Carbamidomethyl (C)	1093.4843	3277.431	3	0.78	K431

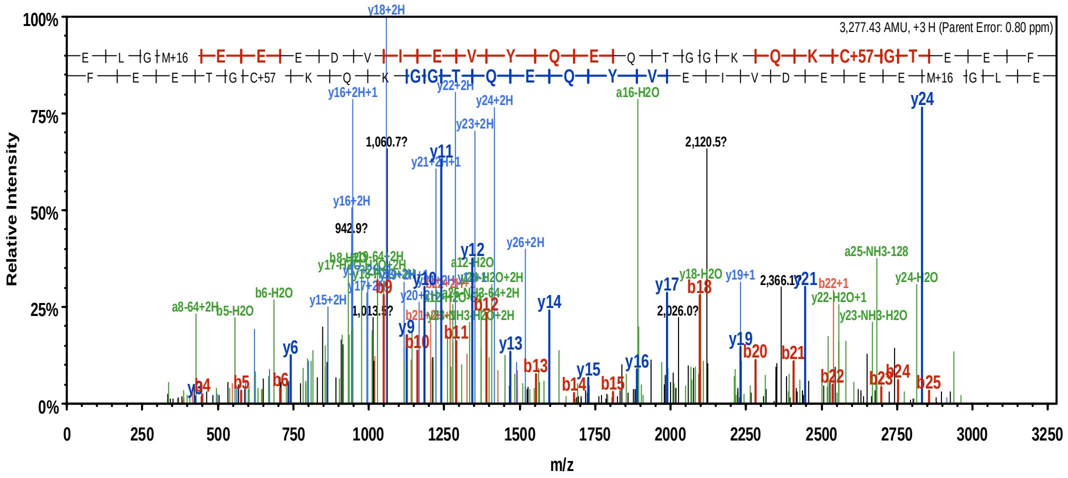

B	B Ions	B+2H	B-NH3	B-H2O	AA	Y Ions	Y+2H	Y-NH3	Y-H2O	Y
1	130.0			112.0	E	3,278.4	1,639.7	3,261.4	3,260.4	28
2	243.1			225.1	L	3,149.4	1,575.2	3,132.4	3,131.4	27
3	300.2			282.1	G	3,036.3	1,518.7	3,019.3	3,018.3	26
4	447.2			429.2	M+16	2,979.3	1,490.1	2,962.3	2,961.3	25
5	576.2			558.2	E	2,832.3	1,416.6	2,815.2	2,814.2	24
6	705.3	353.1		687.3	E	2,703.2	1,352.1	2,686.2	2,685.2	23
7	834.3	417.7		816.3	E	2,574.2	1,287.6	2,557.1	2,556.2	22
8	949.3	475.2		931.3	D	2,445.1	1,223.1	2,428.1	2,427.1	21
9	1,048.4	524.7		1,030.4	V	2,330.1	1,165.6	2,313.1	2,312.1	20
10	1,161.5	581.3		1,143.5	I	2,231.0	1,116.0	2,214.0	2,213.0	19
11	1,290.5	645.8		1,272.5	E	2,117.9	1,059.5	2,100.9	2,099.9	18
12	1,389.6	695.3		1,371.6	V	1,988.9	995.0	1,971.9	1,970.9	17
13	1,552.7	776.8		1,534.7	Y	1,889.8	945.4	1,872.8	1,871.8	16
14	1,680.7	840.9	1,663.7	1,662.7	Q	1,726.8	863.9	1,709.7	1,708.8	15
15	1,809.8	905.4	1,792.7	1,791.8	E	1,598.7	799.9	1,581.7	1,580.7	14
16	1,937.8	969.4	1,920.8	1,919.8	Q	1,469.7	735.3	1,452.6	1,451.7	13
17	2,038.9	1,019.9	2,021.9	2,020.9	T	1,341.6	671.3	1,324.6	1,323.6	12
18	2,095.9	1,048.5	2,078.9	2,077.9	G	1,240.6	620.8	1,223.5	1,222.6	11
19	2,152.9	1,077.0	2,135.9	2,134.9	G	1,183.5	592.3	1,166.5	1,165.5	10
20	2,281.0	1,141.0	2,264.0	2,263.0	K	1,126.5	563.8	1,109.5	1,108.5	9
21	2,409.1	1,205.0	2,392.0	2,391.1	Q	998.4	499.7	981.4	980.4	8
22	2,537.2	1,269.1	2,520.1	2,519.2	K	870.4	435.7	853.3	852.4	7
23	2,697.2	1,349.1	2,680.2	2,679.2	C+57	742.3			724.3	6
24	2,754.2	1,377.6	2,737.2	2,736.2	G	582.2			564.2	5
25	2,855.3	1,428.1	2,838.2	2,837.3	T	525.2			507.2	4
26	2,984.3	1,492.7	2,967.3	2,966.3	E	424.2			406.2	3
27	3,113.4	1,557.2	3,096.3	3,095.3	E	295.1			277.1	2
28	3,278.4	1,639.7	3,261.4	3,260.4	F	166.1				1
