# Supplementary material for: The Human Cytomegalovirus DNA Polymerase Processivity Factor UL44 Is Modified by SUMO in a DNA-Dependent Manner
Source: PLoS One. 2012 Nov 15;7(11):e49630. doi: 10.1371/journal.pone.0049630 (PMC3499415; doi:10.1371/journal.pone.0049630)
Supplement: Table S1 — Oligonucleotides used in this work for cloning and mutagenesis. (DOC) [file pone.0049630.s009.doc]

**Table S1.** Oligonucleotides used in this work for cloning and mutagenesis.

| **Oligonucleotide** | **Nucleotide sequence** |
| --- | --- |
| pCR-Kan/for | 5’-CAATATGACGTCGGGCGGTTTTATGGACAGC-3’ |
| pCR-Kan/rev | 5’-ATATGCCGGAAGGGCCCGCGCACATTTCCCCG-3’ |
| UL44FL/FOR1 | 5’-CACGATGGATCCTTATGGATCGCAAGACGC-3’ |
| UL44FL/REV1 | 5’-TTCCACCTGCAGCTAGCCGCACTTTTGC-3’ |
| UL54FL/FOR2 | 5’-CACGATCCATGGTTATGTTTTTCAACCCG-3 |
| UL54FL/REV2 | 5’-TTCCACGAATTCTCAACAGCATTCGTGCGC-3’ |
| UBC9PGBT9FOR | 5’-CCTGATGAATTCATGTCGGGGATCGCCCTC-3’ |
| UBC9PGBT9REV | 5’-GATGATGTCGACTTATGAGGGCGCAAACTTC-3’ |
| UBC9PRSETAFOR | 5’-CATAATGGATCCATGTCGGGGATCGCCCTCAG-3’ |
| UBC9PRSETAREV | 5’-CTCCACGAATTCTTATGAGGGCGCAAACTTC-3’ |
| UL44 1-100 rev | 5’-AATAATCTGCAGCTAGGTCAGGTCCTTGCTGG-3’ |
| UL44 1-200 rev | 5’-AATAATCTGCAGCTACTCGCTGCCGTTGGTCAG-3’ |
| UL44 1-300 rev | 5’-AATAATCTGCAGCTAAGAGAGGCTGCCGCCACC-3’ |
| UL44 1-350 rev | 5’-AATAATCTGCAGCTACGTACCGGAACCACCGCC-3’ |
| UL44 1-390 rev | 5’-AATAATCTGCAGCTAGCCGCCGCCCGATCCACC-3’ |
| UL44 1-420 rev | 5’-AATTATCTGCAGCTACGTTACAGAATCCTCGC-3’ |
| UL44K73R for | 5’-GCCTCTACATCACTGACCGGTCGTTTCAGCCCAAGACC-3’ |
| UL44K73R rev | 5’-GGTCTTGGGCTGAAACGACCGGTCAGTGATGTAGAGGC-3’ |
| UL44K167R for | 5’-GGGCCGCATACCCGCGTCCGGCGTAACGTTAAAAAAGCG-3’ |
| UL44K167R rev | 5’-CGCTTTTTTAACGTTACGCCGGACGCGGGTATGCGGCCC-3’ |
| UL44K172R for | 5’-GTCAAGCGTAACGTTAAACGAGCGCCCTGCCCTACGGGC-3’ |
| UL44K172R rev | 5’-GCCCGTAGGGCAGGGCGCTCGTTTAACGTTACGCTTGAC-3’ |
| UL44K224R for | 5’-CGTATCAACGTGCAGCTGCGGAACTTCTACCAGACGCTG-3’ |
| UL44K224R rev | 5’-CAGCGTCTGGTAGAAGTTCCGCAGCTGCACGTTGATACG-3’ |
| UL44K339R for | 5’-GGCGGCGGTGGCGGCAAGCGGCACGACCGCGGTGGCGGC-3’ |
| UL44K339R rev | 5’-GCCGCCACCGCGGTCGTGCCGCTTGCCGCCACCGCCGCC-3’ |
| UL44K371R for | 5’-CGGTCTTTCCTCCAAGGAACGATACGAGCAGCACAAGATC-3’ |
| UL44K371R rev | 5’-GATCTTGTGCTGCTCGTATCGTTCCTTGGAGGAAAGACCG-3’ |
| UL44K410R for | 5’-GGCAATTACTTCAACGACGCGCGGGAGGAGAGCGACAGC-3’ |
| UL44K410R rev | 5’-GCTGTCGCTCTCCTCCCGCGCGTCGTTGAAGTAATTGCC-3’ |
| UL44K431R for | 5’-GTCCCTAACACCAAGAAGCAACGGTGCGGCTAG-3’ |
| UL44K431R rev | 5’-CTAGCCGCACCGTTGCTTCTTGGTGTTAGGGAC-3’ |
| L86A-L87A/for | 5’-AATTCCACGCCGGCGGCGGGTAATTTCATGTACCTGACTTCC-3’ |
| L86A-L87A/rev | 5’-GGAAGTCAGGTACATGAAATTACCCGCCGCCGGCGTGGAATT-3’ |
| Ubc9C93S/F | 5’-GGGACAGTGTCCCTGTCCATCTTAGAGGAGGA-3’ |
| Ubc9C93S/R | 5’-GGACAGGGACACTGTCCCCGAAGGGTACACAT-3’ |
| UL44attB1(1) | 5´-GGGGACAAGTTTGTACAAAAAAGCAGGCTTCGATCGCAAGACGCGCCT  CTCGGAGC-3’ |
| UL44attB1(313) | 5´-GGGGACAAGTTTGTACAAAAAAGCAGGCTTCCCGGGTCTGGATAACGA  TCTCATG-3’ |
| UL44attB2(300) | 5´-GGGGACCACTTTGTACAAGAAAGCTGGGTCTAAGAGAGGCTGCCGCCA  CCACCGCCG-3’ |
| UL44attB2(433) | 5’-GGGGACCACTTTGTACAAGAAAGCTGGGTCTAGCCGCACTTTTGCTTC  TTGG-3’ |
