# Supplementary material for: The Human Cytomegalovirus DNA Polymerase Processivity Factor UL44 Is Modified by SUMO in a DNA-Dependent Manner
Source: PLoS One. 2012 Nov 15;7(11):e49630. doi: 10.1371/journal.pone.0049630 (PMC3499415; doi:10.1371/journal.pone.0049630)
Supplement: Text S1 — Supplementary Material and Methods and Supplementary References. (DOC) [file pone.0049630.s010.doc]

## Text S1

## Supplementary Material and Methods

## Plasmid construction and mutagenesis

## To generate the pBTMK plasmid, ampicillin resistance gene was cleaved from pBTM116 [1] with AatII and BglI enzymes and substituted with kanamycin resistance gene. Kanamycin resistance gene was amplified from plasmid pCR BluntII TOPO (Invitrogen) with primers pCR-Kan/for e pCR-Kan/rev and the resulting PCR product was cloned in the AatII/BglI sites of pBTM116. The bait plasmid pBTMK-UL44, which expresses the LexA-UL44 fusion protein used for the yeast two-hybrid (Y2H) screenings, was constructed by PCR amplification of UL44 coding sequence from pRSET44 (a gift from P. F. Ertl, GlaxoSmithKline, Stevenage, United Kingdom) with primers UL44FL/FOR1 and UL44FL/REV1 and cloned into the BamHI/PstI sites of pBTMK. To construct pBTMK-Ubc9, Ubc9 coding sequence was amplified from pACT2-Ubc9 plasmid with primers UBC9PGBT9FOR and UBC9PGBT9REV, and cloned into the EcoRI/SalI sites of pBTMK. To generate pACT-UL54 plasmid, which encodes a GAD-UL54 fusion protein, UL54 coding sequence was amplified from pRSET-Pol (a gift from P. F. Ertl) by PCR with primers UL54FL/FOR2 and UL54FL/REV2 and cloned into the NcoI/EcoRI sites of pACT2 (Clontech). To obtain plasmid pRSET-Ubc9, Ubc9 coding sequence was amplified from pACT-Ubc9 plasmid with primers UBC9PRSETAFOR and UBC9PRSETAREV, and cloned into the BamHI/EcoRI sites of pRSETA (Invitrogen).

For mapping UL44 region(s) involved in the interaction with Ubc9, the deletion mutants LexA-UL44_1-100_, LexA-UL44_1-200_, LexA-UL44_1-300_, LexA-UL44_1-350_, LexA-UL44_1-390_, and LexA-UL44_1-420_ were generated by PCR amplification using plasmid pBTMK-UL44 as a template, UL44FL/FOR1 as a forward primer for all constructs, and UL44 1-100 rev, UL44 1-200 rev, UL44 1-300 rev, UL44 1-350 rev, UL44 1-390 rev, and UL44 1-420 rev, respectively, as a reverse primer. The resulting PCR products were cloned in the *Bam*HI/*Pst*I sites of the pBTMK vector. The LexA-UL44_114-433_, LexA-UL44_201-433_, and LexA-UL44_313-433_ constructs were generated by deleting part of the *UL44* coding sequence from the pBTMK-UL44 plasmid with *Bam*HI/*Bgl*II, *Eco*RI, or *SmaI*, respectively. Plasmids pDNR207-UL44(1-300) and pDNR207(313-433) were generated by PCR amplification of pDNR207-UL44 [2] with appropriate oligo pairs containing the attB1 and attB2 recombination sites (see Supplementary Table S1). These constructs were then used to perform LR recombination reactions with the Gateway system compatible expression plasmid pDESTnFLAG [3] to generate mammalian expression vectors encoding FLAG-tagged fusion proteins. All other UL44 mutants were obtained by using the QuikChange mutagenesis kit (Stratagene), amplifying the pRSET44 or the pCDNA3.1-UL44-FLAG plasmid with primer pairs containing appropriate nucleotide change(s) (see Supplementary Table S1). The pDsRed2-Ubc9C93S plasmid, which expresses a mutant of Ubc9, was obtained by using the QuikChange mutagenesis kit and amplifying the pDsRed2-Ubc9 plasmid with oligonucleotides Ubc9C93S/F and Ubc9C93S/R.

Y2H screenings

The LexA-UL44 bait was used to screen two different cellular cDNA libraries. The first screen was performed with a cDNA library derived from B lymphocytes and fused to *S. cerevisiae* GAL4 activation domain (GAD) ([4]; a gift from S. J. Elledge, Harvard University Medical School, Boston, MA, USA). Primary transformants were selected for growth on -His-Leu-Trp dropout plates. Among 4 × 10^6^ transformants, 497 clones were identified that activated *HIS3* reporter gene expression in the presence of LexA-UL44; of these, 167 clones were also positive for β-galactosidase expression. Interactor library plasmids from clones positives in both assays were rescued by transformation of competent *E. coli* DH5α cells with total yeast DNA under ampicillin selection. The specificity of the interaction was confirmed by retransforming the plasmids expressing putative UL44-interacting partners into yeast strain L40 together with pBTMK or pBTMK-UL44, expressing LexA alone or LexA-UL44, respectively, and then assaying the transformants by filter lift experiments. Only 28 library plasmids demonstrated a requirement of LexA-UL44 for activation of both reporter genes and were thus sequenced and analyzed with BLAST (www.ncbi/blastn).

In the second screen, Lex-UL44-expressing yeasts were transformed with a cDNA library from HL-60 cells ([5]; a gift from S. P. Goff, Columbia University, New York, NY, USA). Approximately 5.7 × 10^6^ transformants were obtained, and 572 clones that activated *HIS3* gene expression were identified; 85 colonies were ultimately obtained that were also positive in β-galactosidase assays. As above, plasmids encoding putative interactors of UL44 were isolated from double-positive clones and retransformed into yeasts expressing LexA-UL44 in order to confirm the interaction. The 13 positive clones after this retransformation were sequenced and analyzed as above.

## Protein purification

## GST and GST-UL44 were purified as previously described [6]. Wild-type and mutant 6His-UL44 fusion proteins were purified from E. coli BL21(DE3)/pLysS harboring the appropriate pRSET44 plasmid. Typically, cells were grown in LB medium containing 100 µg/ml ampicillin until the OD_600_ was 0.8 and then induced by the addition of 0.3 mM IPTG for 3 h at room temperature (RT). Cells were pelleted, resuspended in lysis buffer (50 mM NaH_2_PO_4_, 500 mM NaCl, 10% glycerol, 10 mM imidazole, 1 mg/ml lysozyme, and Complete protease inhibitors), and then lysed by two freeze/thaw cycles followed by sonication. The lysate was centrifuged at 13,000 rpm for 45 min, applied to a 0.5-ml Ni-NTA agarose resin column (Qiagen) that had been equilibrated in lysis buffer, and then washed with wash buffer 1 (50 mM NaH_2_PO_4_, 500 mM NaCl, 10% glycerol, and 20 mM imidazole) and subsequently with wash buffer 2 (50 mM NaH_2_PO_4_, 500 mM NaCl, 10% glycerol, and 50 mM imidazole). Finally, proteins were eluted with elution buffer (50 mM NaH_2_PO_4_, 500 mM NaCl, 10% glycerol, and 250 mM imidazole). Purified proteins were dialyzed against 20 mM Tris-HCl pH 7.5, 150 mM NaCl, 30% glycerol, 0.1 mM EDTA, 2 mM DTT and stored at -80°C.

## GST-pulldown assays

## For the analysis of the binding of UL44 to UL54, purified GST or GST-UL44 protein (75 µg) was incubated with 25 µl of in vitro-translated UL54 for 2 h on ice in binding buffer 1 (50 mM Tris-HCl pH 7.5, 150 mM NaCl, 10% glycerol, 0.1 mM EDTA, 2 mM DTT) containing 2.5 µl of RNAce-It RNase Cocktail (Stratagene) and 25 U of Benzonase (Sigma). UL54 was translated in vitro from pRSET-Pol plasmid by using the TNT T7 coupled reticulocyte lysate system (Promega) according to the manufacturer’s suggestion and labeled with [^35^S]methionine (Amersham Pharmacia Biotech). The binding reactions were then loaded onto 0.2-ml glutathione columns. The columns were washed with 5 ml of wash buffer 1 (50 mM Tris-HCl pH 7.5, 500 mM NaCl, 10% glycerol, 0.1 mM EDTA, 2 mM DTT, 0.5% NP-40, and 0.5% Triton X-100). Bound proteins were then eluted with wash buffer 1 containing 15 mM glutathione.

For the analysis of the binding of UL44 to Ubc9 or influenza A virus PB1 protein, the same protocol was used, with minor modifications. Briefly, GST or GST-UL44 (75 µg) was incubated with 25 µl of *in vitro*-translated, [^35^S]-labeled Ubc9 (from pRSETA-Ubc9 plasmid) or PB1 (from pCDNA-PB1 plasmid [7]) for 1 h at RT in binding buffer 2 (50 mM Tris-HCl pH 7.5, 150 mM NaCl, 10% glycerol, 0.1 mM EDTA, 2 mM DTT, 0.5% NP-40) and the columns were washed with 5 ml of binding buffer 2. Finally, bound proteins were eluted with binding buffer 2 supplemented with 15 mM glutathione. The proteins were visualized by sodium dodecyl sulfate-polyacrylamide gel electrophoresis (SDS-PAGE) and autoradiography.

*E. coli* SUMO expression/modification system

The pTE1E2S1 plasmid (kindly provided by H. Saitoh, Kusamoto University, Japan), which contains a linear fusion of genes for E1 and E2 enzymes and SUMO-1 under the control of an IPTG-inducible promoter [8], was introduced into the *E. coli* BL21(DE3) strain together with the pRSET44, which encodes a 6His-UL44 fusion protein. Expression and SUMO modification of UL44 was accomplished as described previously [8]. Purification of sumoylated 6His-UL44 from these cultures was carried out as described above.

Mass spectrometry

*E. coli*-expressed*,* SUMO-conjugated UL44 (prepared as described above) was excised from gel, reduced with 50 mM DTT, alkylated with 100 mM iodoacetic acid and in-gel digested with modified trypsin (Promega) overnight, all at 37°C. Tryptic peptides were dissolved in 2 μl of 50% acetonitrile with 0.1 % formic acid, 18 μl 0.1 % formic acid for further MS analysis. Mass spectrometric analysis was performed by Orbitrap-Velos mass spectrometer (Thermo Fisher Scientific) equipped with a nanoelectrospray ion source and coupled to an Agilent 1100 HPLC system (Agilent Technologies), fitted with a home-made C18 column. Tryptic peptides were first loaded at a flow rate of 10 μl/min onto a C18 trap column (1.5 cm, 360 μm o.d., 150 mm i.d., Reprosil-Pur 120Å, 5 μm, C18-AQ, Dr. Maisch GmbH, Germany). Retained peptides were eluted and separated on an analytical C18 capillary column (15 cm, 360 μm o.d., 75 μm i.d., Reprosil-Pur 120Å, 5μm, C18-AQ, Dr. Maisch GmbH, Germany) at a flow rate of 300 nl/min, with a gradient from 7.5 to 37.5 % ACN in 0.1 % formic acid for 60 min. Typical mass spectrometric conditions were: spray voltage, 1.6 kV; heated capillary temperature, 270°C; normalized CID collision energy 37.5 % for MS/MS in LTQ. An activation q = 0.25 and activation time of 30 ms were used. The mass spectrometer was operated in the data dependent mode to automatically switch between MS and MS/MS acquisition. Survey full scan MS spectra (from m/z 350-2000) were acquired in the orbitrap with resolution R = 30,000 at m/z 400 (after accumulation to a ‘target value’ of 1,000,000 in the orbitrap). The fifteen most intense ions were sequentially isolated and fragmented in the linear ion trap using collision induced dissociation (CID) at a target value of 100,000. For all measurements with the orbitrap detector, a lock-mass ion from ambient air (m/z 445.120025) was used for internal calibration.

Identification of sumoylated lysine residues was performed as described in [9]. We used the “ChopNSpice” software ([www.chopnspice.gwdg.de](http://www.chopnspice.gwdg.de/)) to generate a concatenated protein sequence in order to identify the actual SUMO-sites with MASCOT a search engine. For this, the FASTA sequence of UL44 was chopped into tryptic fragments allowing 0, 1, 2, 3 missed cleavages. The tryptic peptide sequence of SUMO-1 that is putatively attached to any lysine residue within UL44 is attached to the N-terminus of each tryptic peptide of UL44 that contains a lysine residue or a missed cleavage site. To avoid the generation of non-natural peptides, a virtual amino acid “J” is attached to the C-terminus of each tryptic fragment derived from UL44 and SUMO-1. The modified tryptic fragments are concatenated to yield a novel large FASTA sequence that is submitted into database search. Upon database search with the search engine MASCOT, cleavage with an artificial endoproteinase was allowed that specifically recognizes N- and C-terminal “J” and an user defined number of missed cleavages. The search engine then compares the *in silico* generated and concatenated tryptic peptides derived from UL44 attached to a tryptic peptide derived from SUMO-1 with the experimentally obtained fragment spectra by LC-MSMS. The following parameters were used in “ChopNSpice” software: spice species was *H. sapiens*; spice sequence was SUMO-1; spice site was KX; spice mode was once per fragment; include unmodified fragments in output; enzyme was trypsin; allow up to three protein miscleavages; allow up to one miscleavage in the spice sequence; output formatting was FASTA (single protein sequence); mark all cleaved sites J; retain comments in FASTA format without line breaks in FASTA output. For sumoylated site identification with MASCOT, all MSMS spectra were searched against a new FASTA file that was created by ChopNSpice with the following parameters: mass tolerance of 10 ppm in MS mode and 0.8 Da in MSMS mode; allow zero missed cleavages; consider methionine oxidation and cysteine carboxyamidomethylation as variable modifications; enzyme cleaved at J at N- and C-termini for MASCOT.

Antibodies for western blotting and immunoprecipitation

For western blotting the following primary antibodies were used: anti-UL44 mouse monoclonal antibody (Fitzgerald Industries International, 1:1,000, overnight at 4°C), anti-LexA antibody (Invitrogen, 1:5,000, for 2 h at RT), anti-p53 antibody (Active Motif, 1:5,000, overnight at 4°C), anti-FLAG M2 monoclonal antibody (Sigma, 1:2,500, for 1 h at 4°C), anti-Ubc9 rabbit antibody (Santa Cruz Biotechnology, 1:500, overnight at 4°C), anti-HA mouse antibody (produced at the IFOM-IEO-Campus, Milan, Italy; 1 µg/ml, for 2 h at RT), anti-GMP1 mouse antibody (SUMO-1) (Zymed, 1:500, overnight at 4°C) or anti-SUMO-1 and anti-SUMO-2/-3 rabbit polyclonal antibody (Enzo Life Science, both 1:1,000, for 1 h at RT), anti-α-tubulin mouse antibody (Sigma, 1:1,000, for 2 h at RT), anti-GAPDH mouse antibody (Abcam, 1:10,000, for 1 h at RT), anti-vinculin mouse antibody (produced at the IFOM-IEO-Campus, Milan, Italy; 1:10,000 for 1 h at RT), anti-V5 mouse antibody (Serotec, 1:1,000, for 2 h at RT), anti-Cyclin D1 rabbit polyclonal antibody (Santa Cruz Biotechnology, 1:1,000, overnight at 4°C), and anti-UL57 mouse monoclonal antibody (Virusys, 1:100, for 1 h at 37°C). After washes, the blots were incubated with horseradish peroxidase-conjugated anti-mouse (Santa Cruz Biotechnology; 1:2000) or anti-rabbit (Pierce; 1:500) secondary antibodies for 1 h at RT. Protein bands were visualized using LiteAblot Extend Long Lasting Chemiluminescent Substrate kit (EuroClone) or ECL kit (Amersham Pharmacia Biotech) according to the manufacturers’ protocol.

For immunoprecipitations the following antibodies were used: anti-FLAG rabbit polyclonal antibody (Abcam, 3 µg/mg of protein lysate, overnight at 4°C) and anti-UL44 mouse monoclonal antibody (Fitzgerald Industries International, 7 µg/mg of protein lysate, overnight at 4°C).

**Supplementary References**

1. Moretti P, Freeman K, Coodly L, Shore D (1994) Evidence that a complex of SIR proteins interacts with the silencer and telomere-binding protein RAP1. Genes Dev 8: 2257-2269.

2. Alvisi G, Ripalti A, Ngankeu A, Giannandrea M, Caraffi SG, et al. (2006) Human cytomegalovirus DNA polymerase catalytic subunit pUL54 possesses independently acting nuclear localization and ppUL44 binding motifs. Traffic 7: 1322-1332.

3. Panza E, Marini M, Pecci A, Giacopelli F, Bozzi V, et al. (2008) Transfection of the mutant MYH9 cDNA reproduces the most typical cellular phenotype of MYH9-related disease in different cell lines. Pathogenetics 1: 5.

4. Durfee T, Becherer K, Chen PL, Yeh SH, Yang Y, et al. (1993) The retinoblastoma protein associates with the protein phosphatase type 1 catalytic subunit. Genes Dev 7: 555-569.

5. Kalpana GV, Marmon S, Wang W, Crabtree GR, Goff SP (1994) Binding and stimulation of HIV-1 integrase by a human homolog of yeast transcription factor SNF5. Science 266: 2002-2006.

6. Loregian A, Appleton BA, Hogle JM, Coen DM (2004) Residues of human cytomegalovirus DNA polymerase catalytic subunit UL54 that are necessary and sufficient for interaction with the accessory protein UL44. J Virol 78: 158-167.

7. Muratore G, Goracci L, Mercorelli B, Foeglein A, Digard P, et al. (2012) Small molecule inhibitors of influenza A and B viruses that act by disrupting subunit interactions of the viral polymerase. Proc Natl Acad Sci U S A 109: 6247-6252.

8. Uchimura Y, Nakamura M, Sugasawa K, Nakao M, Saitoh H (2004) Overproduction of eukaryotic SUMO-1- and SUMO-2-conjugated proteins in Escherichia coli. Anal Biochem 331: 204-206.

9. Hsiao HH, Meulmeester E, Frank BT, Melchior F, Urlaub H (2009) "ChopNSpice," a mass spectrometric approach that allows identification of endogenous small ubiquitin-like modifier-conjugated peptides. Mol Cell Proteomics 8: 2664-2675.
